# Estimating the subnational prevalence of antimicrobial resistant *Salmonella enterica* serovars Typhi and Paratyphi A infections in 75 endemic countries, 1990–2019: a modelling study

**DOI:** 10.1016/S2214-109X(23)00585-5

**Published:** 2024-02-14

**Authors:** GRAM Typhoid Collaborators, Annie J Browne, Michael G Chipeta, Frederick J Fell, Georgina Haines-Woodhouse, Bahar H Kashef Hamadani, Emmanuelle A P Kumaran, Gisela Robles Aguilar, Barney McManigal, Jason R Andrews, Elizabeth A Ashley, Allan Audi, Stephen Baker, Happy C Banda, Buddha Basnyat, Godfrey Bigogo, Chanpheaktra Ngoun, Vilada Chansamouth, Angeziwa Chunga, John D Clemens, Viengmon Davong, Gordon Dougan, Susanna J Dunachie, Nicholas A Feasey, Denise O Garrett, Melita A Gordon, Rumina Hasan, Andrea H Haselbeck, Nathaniel J Henry, Robert S Heyderman, Marianne Holm, Hyon Jin Jeon, Abhilasha Karkey, Farhana Khanam, Stephen P Luby, Faisal Riaz Malik, Florian Marks, Mayfong Mayxay, James E Meiring, Catrin E Moore, Patrick K Munywoki, Patrick Musicha, Paul N Newton, Gideok Pak, Koukeo Phommasone, Sunil Pokharel, Andrew J Pollard, Firdausi Qadri, Farah N Qamar, Sayaphet Rattanavong, Bobby Reiner, Tamalee Roberts, Samir Saha, Senjuti Saha, Sadia Shakoor, Mila Shakya, Andrew J Simpson, Jeff Stanaway, Claudia Turner, Paul Turner, Jennifer R Verani, Manivanh Vongsouvath, Nicholas P J Day, Mohsen Naghavi, Simon I Hay, Benn Sartorius, Christiane Dolecek

## Abstract

**Background:**

Enteric fever, a systemic infection caused by *Salmonella enterica* serovars Typhi and Paratyphi A, remains a major cause of morbidity and mortality in low-income and middle-income countries. Enteric fever is preventable through the provision of clean water and adequate sanitation and can be successfully treated with antibiotics. However, high levels of antimicrobial resistance (AMR) compromise the effectiveness of treatment. We provide estimates of the prevalence of AMR *S* Typhi and *S* Paratyphi A in 75 endemic countries, including 30 locations without data.

**Methods:**

We used a Bayesian spatiotemporal modelling framework to estimate the percentage of multidrug resistance (MDR), fluoroquinolone non-susceptibility (FQNS), and third-generation cephalosporin resistance in *S* Typhi and *S* Paratyphi A infections for 1403 administrative level one districts in 75 endemic countries from 1990 to 2019. We incorporated data from a comprehensive systematic review, public health surveillance networks, and large multicountry studies on enteric fever. Estimates of the prevalence of AMR and the number of AMR infections (based on enteric fever incidence estimates by the Global Burden of Diseases study) were produced at the country, super-region, and total endemic area level for each year of the study.

**Findings:**

We collated data from 601 sources, comprising 184 225 isolates of *S* Typhi and *S* Paratyphi A, covering 45 countries over 30 years. We identified a decline of MDR *S* Typhi in south Asia and southeast Asia, whereas in sub-Saharan Africa, the overall prevalence increased from 6·0% (95% uncertainty interval 4·3–8·0) in 1990 to 72·7% (67·7–77·3) in 2019. Starting from low levels in 1990, the prevalence of FQNS *S* Typhi increased rapidly, reaching 95·2% (91·4–97·7) in south Asia in 2019. This corresponded to 2·5 million (1·5–3·8) MDR *S* Typhi infections and 7·4 million (4·7–11·3) FQNS *S* Typhi infections in endemic countries in 2019. The prevalence of third-generation cephalosporin-resistant *S* Typhi remained low across the whole endemic area over the study period, except for Pakistan where prevalence of third-generation cephalosporin resistance in *S* Typhi reached 61·0% (58·0–63·8) in 2019. For *S* Paratyphi A, we estimated low prevalence of MDR and third-generation cephalosporin resistance in all endemic countries, but a drastic increase of FQNS, which reached 95·0% (93·7–96·1; 3·5 million [2·2–5·6] infections) in 2019.

**Interpretation:**

This study provides a comprehensive and detailed analysis of the prevalence of MDR, FQNS, and third-generation cephalosporin resistance in *S* Typhi and *S* Paratyphi A infections in endemic countries, spanning the last 30 years. Our analysis highlights the increasing levels of AMR in this preventable infection and serves as a resource to guide urgently needed public health interventions, such as improvements in water, sanitation, and hygiene and typhoid fever vaccination campaigns.

**Funding:**

Fleming Fund, UK Department of Health and Social Care; Wellcome Trust; and Bill and Melinda Gates Foundation.

## Introduction

*Salmonella enterica* serovars Typhi and Paratyphi A are human-restricted Gram-negative bacterial pathogens that cause serious bloodstream infections, collectively termed enteric fever.^1^, ^2^ Enteric fever is transmitted through contaminated water or food and is primarily a disease of low-income and middle-income countries (LMICs).^1^ It is a major cause of febrile illness in south Asia, southeast Asia, and sub-Saharan Africa. In 2017, global data indicated 14·3 million enteric fever infections and 135 000 related deaths.^3^

Enteric fever can be successfully treated with antibiotics; ampicillin or amoxicillin, chloramphenicol, trimethoprim–sulfamethoxazole, fluoroquinolones (ciprofloxacin or ofloxacin), third-generation cephalosporins (ceftriaxone or cefixime), or azithromycin are recommended treatment options.^4^ Antimicrobial resistance (AMR) in *S* Typhi and *S* Paratyphi A is now a major concern, especially in parts of the world with little access to alternative antibiotics. Multidrug resistance (MDR; concurrent resistance to ampicillin or amoxicillin, chloramphenicol, and trimethoprim–sulfamethoxazole) and fluoroquinolone non-susceptibility (FQNS) are both frequently reported in enteric fever.^5^ Since 2016, extensively drug-resistant (defined as MDR, FQNS, and third-generation cephalosporin resistance) *S* Typhi isolates have been reported in Pakistan,^6^ and azithromycin-resistant isolates in Nepal, India, and Bangladesh.^7^, ^8^, ^9^ Emergence of AMR can lead to treatment failure, with a higher rate of complications and death, along with an increased risk of onwards transmission.^10^ Consequently, WHO listed *S* Typhi as a high-priority pathogen for the development of new antimicrobial therapies.^11^

**Research in context**

**Evidence before this study**

The widespread emergence of antimicrobial-resistant (AMR) *Salmonella enterica* serovars Typhi and Paratyphi A poses a significant challenge to the clinical management of this preventable infection. Over the past three decades, numerous hospital-based reports, multicountry population-based surveillance studies, and systematic reviews have highlighted the continuous rise of AMR enteric fever in low-income and middle-income countries (LMICs); however, there are gaps in our knowledge due to the scarcity of robust AMR surveillance in many LMICs. We aimed to collate the available data and develop a spatiotemporal model to estimate the prevalence of AMR in LMICs, including locations with no data. We searched PubMed for existing modelling studies that determined the prevalence of AMR, using the keywords (“*Salmonella* Typhi” OR “*Salmonella* Paratyphi A” OR “typhoid fever” OR “paratyphoid fever” OR “enteric fever”) AND (“antimicrobial resistance” OR “antibiotic resistance” OR “drug resistance”) AND (“prevalence of resistance”) AND “model” in the abstract, for all studies published before Sep 13, 2023, without language restrictions. We identified one study that estimated the effect of vaccination on both the burden of typhoid fever and the reduction of drug-resistant typhoid fever cases in 73 countries eligible for support from GAVI, the Vaccine Alliance. The study used the systematic review on drug-resistant enteric fever by Browne and colleagues as the basis for their predictions, which also served as a foundation of this study. We did not identify a modelling study that made predictions for the prevalence of AMR enteric fever in LMICs.

**Added value of this study**

This study advances existing research by providing a comprehensive assessment of the prevalence of AMR enteric fever in 75 LMICs where enteric fever is endemic. We provide the first detailed estimates of the proportion of multidrug resistant (MDR), fluoroquinolone non-susceptibility (FQNS), and third-generation cephalosporin-resistant *S* Typhi and *S* Paratyphi A infections at the administrative level one district, country, super-region, and total endemic area level, covering 30 years from 1990 to 2019. In addition, we estimated the numbers of AMR infections, based on enteric fever incidence estimates by the Global Burden of Diseases study, at the country, super-region, and total endemic area level, from 1990 to 2019.

**Implications of all the available evidence**

Our analysis highlights the increasing magnitude and geographical spread of AMR enteric fever over the past 30 years. In 2019, issues of particular concern were the high prevalence of MDR *S* Typhi (72·7% [95% uncertainty interval 67·7–77·3]) and FQNS *S* Typhi (19·7% [10·9–30·6]) in sub-Saharan Africa, the high prevalence of FQNS *S* Typhi (95·2% [91·4–97·7]) in south Asia, and the high proportions (61·0% [58·0–63·8]) of third-generation cephalosporin-resistant *S* Typhi in Pakistan. For *S* Paratyphi A, the predominant issue was FQNS, which reached 95·0% [93·7–96·1] in the endemic region in 2019. Our results emphasise the importance and urgent need for investments into public health infrastructure globally to provide universal access to clean water, adequate sanitation, and basic hygiene (WASH) as set out in the Sustainable Development Goal 6. Improvements in WASH will also prevent other waterborne diseases and contribute to social justice and gender equity. As part of an integrated approach to control AMR *S* Typhi and *S* Paratyphi A, typhoid fever vaccination campaigns, development of licensed paratyphoid fever vaccines, universal health care, and antibiotic stewardship are needed.

Countries where enteric fever is endemic frequently do not have the laboratory capacity required to accurately identify bacterial isolates and conduct antimicrobial susceptibility testing (AST).^12^, ^13^ There is little understanding of the prevalence of AMR in *S* Typhi and *S* Paratyphi A at both a national and subnational scale, with few surveillance networks reporting on AMR in enteric fever.^14^, ^15^, ^16^, ^17^ Data are primarily from individual hospitals and institutions with scarce spatial and temporal comparability. Knowledge of the magnitude and trends of AMR enteric fever could inform targeted public health interventions, such as vaccination campaigns and improvements in water, sanitation, and hygiene (WASH).

Research has used whole-genome sequencing to explore emergence of AMR and geographical spread of *S* Typhi in Asia and globally.^18^, ^19^ However, a comprehensive and detailed analysis of phenotypic resistance in both *S* Typhi and Paratyphi A to discern AMR trends over multiple decades across the endemic region is unavailable.

Spatiotemporal modelling is an effective tool for estimating the burden of infectious diseases and health indicators in locations where data are scarce.^20^, ^21^, ^22^, ^23^ In this study, we took an extensive dataset of geolocated studies on the prevalence of AMR in *S* Typhi and *S* Paratyphi A and use spatiotemporal modelling techniques to estimate the prevalence of MDR, FQNS, and third-generation cephalosporin resistance in *S* Typhi and *S* Paratyphi A for each administrative level one district in countries with endemic disease for each year, from 1990 to 2019. These estimates are then applied to the Global Burden of Disease (GBD) estimates of disease incidence,^24^ to calculate the overall numbers of drug-resistant and drug-susceptible enteric fever infections per year.

Here, we expand on our previous prevalence of resistance estimates for *S* Typhi and *S* Paratyphi A that were produced by the authors as part of the global burden of bacterial AMR in 2019,^23^ by incorporating additional data and using a subnational spatiotemporal modelling framework to produce detailed estimates of the prevalence of AMR in *S* Typhi and *S* Paratyphi A spanning 30 years.

## Methods

### Study design

In our modelling study, we used a two-stage spatiotemporal modelling framework to estimate the prevalence of MDR, FQNS, and third-generation cephalosporin resistance in *S* Typhi and *S* Paratyphi A infections for 1403 administrative level one districts (typically corresponding to state, province, region, or the equivalent depending on each country's nomenclature) in 75 LMICs endemic for typhoid or paratyphoid fever, from 1990 to 2019. Endemic countries were classified as those with an incidence rate of at least ten cases per 100 000 population per year, based on GBD estimates (appendix 1 p 5).^3^ Island nations with populations under two million people were excluded from the analysis due to a scarcity of data and geographic contiguity to inform spatial models. *S* Typhi was classified as endemic in the south Asia super-region, southeast Asia, east Asia, and Oceania super-region, north Africa and the Middle East super-region, and sub-Saharan Africa super-region (75 countries), but *S* Paratyphi A was only classified as endemic in the south Asia super-region and southeast Asia, east Asia, and Oceania super-region (18 countries), reflecting the different geographic distribution of the two serotypes.

MDR was defined as concurrent resistance to ampicillin, chloramphenicol, and trimethoprim–sulfamethoxazole. When AMR profiles were missing for one of the three antimicrobials that define MDR, we inferred the MDR prevalence on the basis of the two available antimicrobials (appendix 1 p 3). FQNS was defined as isolates with a ciprofloxacin minimum inhibitory concentration (MIC) of 0·125 μg/mL or higher (or corresponding disc zone diameter of ≤30 mm), or nalidixic acid resistance if ciprofloxacin testing results were not available (nalidixic acid resistance was commonly used as a proxy marker for FQNS).^5^, ^10^ FQNS is primarily acquired by point mutations in the quinolone resistance determining region of the bacterial chromosome of *S* Typhi and *S* Paratyphi A.^1^ Third-generation cephalosporin resistance was defined as ceftriaxone resistance.

### Procedures

We used a geolocated dataset of the prevalence of AMR in *S* Typhi and *S* Paratyphi A from a systematic review^5^ (according to PRISMA guidelines^25^ and registered with PROSPERO, CRD42018029432). This systematic review was expanded to include additional studies reporting the prevalence of AMR (appendix 1 pp 1–2), and supplemented with data from public health surveillance systems, large multicentre studies on enteric fever (TSAP,^17^ SEAP^26^, STRATAA^15^) and additional datasets from Kenya, Malawi, and Pakistan (appendix 1 p 71). All data were matched to the administrative level one, in which the study was done. Here, this administrative unit has been referred to as district. Data were disaggregated by location and year where possible.

We modelled the prevalence of MDR, FQNS, and third-generation cephalosporin resistance in *S* Typhi and *S* Paratyphi A separately, implementing the same modelling strategy for all. Insufficient data on the co-occurrence of MDR and third-generation cephalosporin resistance precluded analysis of extensively drug-resistant *S* Typhi. Spatially and temporally explicit covariates to inform the models were identified from several sources (appendix 1 p 4) and lasso penalised regression models were used to select the most influential covariates for each pathogen-drug combination. Due to high heterogeneity in the input data, outliers were identified and removed from the datasets before modelling. Outliers were identified as datapoints, which lay over two times the median absolute deviation of the data from a crude generalised linear mixed effects model fit between the data and selected covariates, or those which scored in the 95th percentile for multiple outlier algorithms in the DDOutlier package in R version 4.2.1. Overall, 4·6% of data were considered outliers (appendix 1 p 3).

Stacked ensemble models were fitted between the input data and selected covariates. The drivers of AMR have been shown to be very complex,^27^, ^28^ and this modelling strategy aids in covariate selection, allows for varied multidirectional covariate effects, incorporates interactions between covariates and has been shown to have a superior predictive performance compared to alternative modelling strategies.^29^

### Statistical analysis

The stacked ensemble model results were then used as covariates in a spatial temporal conditional autoregressive (STCAR) model, fit using Integrated Nested Laplace Approximation version 22.12.16,^30^ in R version 4.2.1. We employed a hierarchical binomial model to estimate the prevalence of resistance including structured spatial (intrinsic Gaussian conditionally autoregressive based on adjacency matrix of boundary neighbours) and an unstructured spatial-temporal interaction term and a random intercept on data source. The model borrows strength from data in space and time to produce estimates of the prevalence of AMR, with accompanying uncertainty intervals (UIs), for each district and year of the study. The full models (stacked ensemble plus STCAR) were validated using 5-fold cross-validation (appendix 1 p 7).

Finally, the national estimates of the prevalence of resistance were calculated as the population weighted mean for each country-year. The total number of drug resistant and drug susceptible infections were calculated by applying these proportions to the national incidence estimates of *S* Typhi and *S* Paratyphi A from the GBD 2019^24^ for each pathogen-antibiotic combination. Estimates of the prevalence and number of AMR infections were produced at the country, super-region, and total endemic area level for each year of the study. Uncertainty was propagated throughout the modelling process and all estimates are presented with 95% UIs. Full details of the input data and the modelling strategy are included in appendix 1. This research was exempted from ethics approval as all analyses were performed on de-identified data. The information was either publicly accessible or provided to us by GRAM collaborators.

### Role of the funding source

The funders of the study had no role in study design, data collection, data analysis, data interpretation, or writing of the report. The corresponding author had full access to all the data in the study and had final responsibility for the decision to submit for publication.

## Results

Data were included from 601 sources and 184 225 isolates of *S* Typhi and *S* Paratyphi A, covering 45 countries over 30 years. This equated to 3008 location-years of data over the six pathogen–drug combinations, with MDR *S* Typhi having the most data and FQNS *S* Paratyphi A the least (appendix 1 pp 1–2). Overall, the distribution of data reflected the burden of enteric fever with 2137 (69·5%) of 3076 of data originating from the South Asia super-region; 395 (12·8%) of 3076 from the South East Asia, East Asia, and Oceania super-region; 366 (11·9%) of 3095 from the sub-Saharan Africa super-region; and 178 (5·8%) 3095) from the North Africa and Middle East super-region. Due to the aggregated nature of the input data and the implemented modelling strategy, each *Salmonella* serovar–antibacterial combination was estimated independently, the results are therefore presented by pathogen–drug combination; no estimates were produced for co-occurrence of resistance (eg, FQNS and MDR combined).

We used a subnational resolution model as our core approach to allow us to fit the model with spatially refined covariates more closely aligned to the subnational administrative unit containing the outcome data; however, there was little subnational variation in the prevalence of antibiotic resistance identified by this study. We calculated the mean relative deviation in the prevalence of MDR and FQNS in districts, compared with the country mean for each country, year, and *Salmonella* serotype. The mean relative deviation did not exceed 4% for any country-year and was under 1% for most country-years (appendix 2). Due to the scarcity of subnational variation, results are reported on the national and super-region resolution.

**Figure 1:**
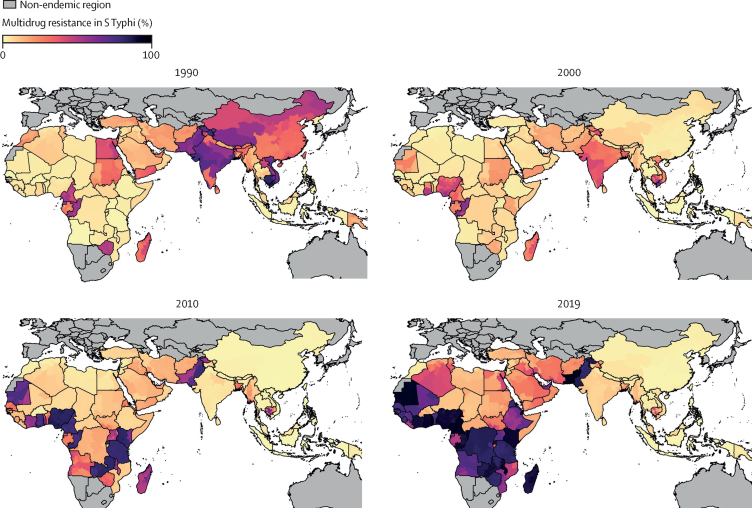
Model estimates of the prevalence of multidrug resistance in *S* Typhi isolates in endemic countries for 1990, 2000, 2010, and 2019 at the administrative division level one resolution Results represent the mean of 1000 draws of the stacked ensemble plus the spatial temporal conditional autoregressive model. Estimates are presented for 75 endemic countries and were not produced for countries deemed non-endemic (shown in grey). Multidrug resistance was defined as concurrent resistance to ampicillin, chloramphenicol, and trimethoprim-sulfamethoxazole.

**Figure 2:**
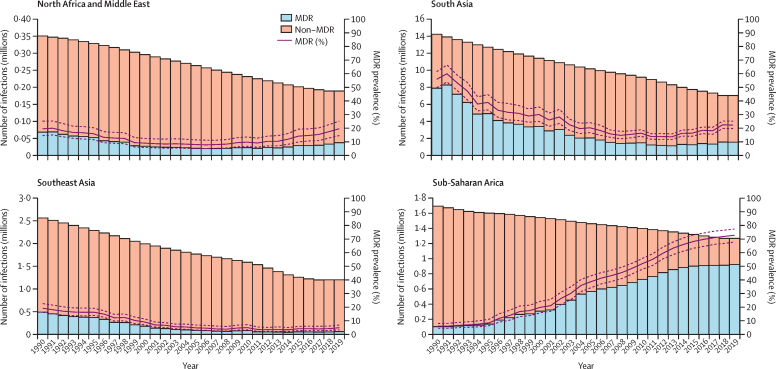
Model estimates of the number of MDR and non-MDR *Salmonella enterica* Typhi infections, and the prevalence of MDR *S* Typhi isolates by GBD super-region and year Stacked bar plots show the number of *S* Typhi infections susceptible to first-line antibiotics (non-MDR; orange) and the number of MDR *S* Typhi infections (blue) for each year, 1990–2019. The prevalence of MDR *S* Typhi infections is shown by the solid red line, with 95% uncertainty intervals shown by the red dashed line. Estimates are shown for modelled disease-endemic countries within each GBD super-region. MDR=multidrug resistance. GBD=Global Burden of Diseases.

The prevalence of MDR *S* Typhi varied greatly both over time and by location (figure 1). Using data from the early 1990s, we estimated a high prevalence of MDR *S* Typhi isolates in south Asia, with 55·4% (95% UI 49·3–61·0) in 1990, equating to an estimated 7·8 million (95% UI 4·9–12·6) MDR infections (appendix 3) when applying to the GBD estimates of *S* Typhi incidence.^3^ This prevalence decreased steadily over time to 26·4% (24·2–28·7) in 2019, corresponding to 2·5 million (1·6–3·8) MDR *S* Typhi infections. Similar trends were observed in the southeast Asia, east Asia, and Oceania super-region, where the prevalence of MDR peaked at 19·0% (16·1–22·4) in 1990, corresponding to 485 102 infections (312 259–728 403), before decreasing to 4·7% (3·2–6·7) by 2019, corresponding to 56 866 (31 990–95 564) infections. In North Africa and the Middle East, prevalence of MDR *S* Typhi remained relatively constant between 1990 and 2019, starting with 19·4% (14·4–25·1; corresponding to 68 016 [40 140–109 703] infections) in 1990. Contrasting trends were identified for sub-Saharan Africa, with the overall prevalence of MDR *S* Typhi increasing from 6·0% (4·3–8·0; 100 903 [57 291–169 368] infections) in 1990 to 72·7% (67·7–77·3; an estimated 922 302 [583 673–1 379 446] infections) in 2019 (figure 2; table).

**Table:** Prevalence and total number of drug-resistant *Salmonella enterica* Typhi and Paratyphi A infections by GBD super-region, 1990 and 2019

	**Prevalence of resistance, 1990**	**Number of drug resistant infections, 1990**	**Prevalence of resistance, 2019**	**Number of drug resistant infections, 2019**
**MDR *S* Typhi**				
North Africa and Middle East	19·4 (14·4–25·1)	68 016 (40 140–109 703)	19·5 (14·5–25·1)	36 817 (21 892–59 214)
South Asia	55·4 (49·3–61·0)	7 817 806 (4 861 263–12 593 675)	21·9 (19·5–24·9)	1 522 393 (935 142–2 321 547)
Southeast Asia, east Asia, and Oceania	19 (16·1–22·4)	485 102 (312 259–728 403)	4·7 (3·2–6·7)	56 866 (31 990–95 564)
Sub-Saharan Africa	6·0 (4·3–8·0)	100 903 (57 291–169 368)	72·7 (67·7–77·3)	922 302 (583 673–1 379 446)
Total (endemic area)	45·2 (40·4–49·6]	8 471 828 (5 289 599–13 542 673]	26·4 (24·4–28·8)	2 538 377 (1 574 044–3 822 395)
**FQNS *S* Typhi**				
North Africa and Middle East	1·1 (0·3–2·6)	3923 (1049–10 096)	35·0 (22·0–48·6)	65 954 (35 678–108 716)
South Asia	0·9 (0·3–2·0)	130 215 (41 712–315 435)	95·2 (91·4–97·7)	6 621 091 (4 162 818–10 210 827)
Southeast Asia, east Asia, and Oceania	1·9 (0·7–3·8)	49 421 (17 211–108 206)	36·4 (28·7–44·8)	434 937 (278 341–661 753)
Sub-Saharan Africa	1·3 (0·4–2·8)	21 762 (6495–52 313)	19·7 (10·9–30·6)	249 612 (116 502–437 343)
Total (endemic area)	1·1 (0·5–2·2)	205 322 (71 483–452 201)	76·6 (71·9–80·8)	7 371 594 (4 696 598–11 304 962)
**Third-generation cephalosporin-resistant *S* Typhi**				
North Africa and Middle East	0·7 (0·3–1·4)	2383 (881–5 341)	1·6 (0·8–3·1)	3078 (1249–6537)
South Asia	2·0 (1·3–3·0)	284 292 (146 848–535 314)	17·9 (15·7–20·7)	1 247 042 (782 227–1 918 397)
Southeast Asia, east Asia, and Oceania	0·7 (0·3–1·4)	16 714 (6 247–40 367)	1·5 (0·7–3)	17 970 (7 348–40 443)
Sub-Saharan Africa	1·6 (0·7–3·1)	26 762 (10 562–62 792)	3·6 (1·9–6·2)	45 378 (21 060–95 222)
Total (endemic area)	1·8 (1·1–2·6)	330 150 (173 586–604 776)	13·7 (11·9–15·7)	1 313 468 (820 108–2 048 670)
**MDR *S* Paratyphi A**				
South Asia	9·4 (2·4–25·2)	592 809 (126 516–1 717 365)	0·2 (0·0–0·4)	5893 (1 318–15 777)
Southeast Asia, east Asia, and Oceania	4·2 (1·0–13·1)	10 469 (1957–29 551)	0·1 (0·0–0·3)	229 (47–659)
Total (endemic area)	9·2 (2·4–24·7)	603 278 (128 385–1 741 281)	0·2 (0·0–0·4)	6122 (1364–16 429)
**FQNS *S* Paratyphi A**				
South Asia	0·6 (0·2–1·4)	37 944 (10 692–95 977)	96·9 (95·6–97·9)	3 354 181 (2 078 575–5 416 423)
Southeast Asia, east Asia, and Oceania	0·9 (0·4–2)	2307 (698–5331)	64·4 (59·5–70·1)	136 820 (88 268–211 771)
Total (endemic area)	0·6 (0·2–1·4)	40 250 (11 462–100 742)	95·0 (93·7–96·1)	3 491 001 (2 167 546–5 618 482)
**Third-generation cephalosporin-resistant *S* Paratyphi A**				
South Asia	0·2 (0·1–0·4)	15 356 (7237–29 430)	0·5 (0·3–0·9)	18 346 (7550–38 759)
Southeast Asia, east Asia, and Oceania	0·3 (0·2–0·4)	658 (305–1240)	3·0 (0·4–11·6)	6477 (738–24 603)
Total (endemic area)	0·2 (0·1–0·4)	16 014 (7553–30 609)	0·7 (0·3–1·5)	24 823 (8578–59 315)

Data are % (95% UI) or n (95% UI). Modelled estimates are shown with 95% UIs for each pathogen-drug combination for each super-region, and for the total endemic area modelled. The number of infections is based on the modelled *S* Typhi and *S* Paratyphi A estimates from the GBD 2019 study.^24^ GBD=Global Burden of Diseases. UI=uncertainty interval. MDR=multidrug resistance. FQNS=fluoroquinolone non-susceptibility.

In 1990, the prevalence of MDR *S* Typhi was estimated to be highest in Cambodia (81·9% [95% UI 68·9–90·8]), Viet Nam (75·6% [66·1–84·2]), Pakistan (63·9% [52·5–74·3]), and India (58·1% [50·6–64·9]). In southeast Asia, east Asia, and Oceania, despite the extremely high prevalence of MDR in Cambodia and Viet Nam, only very low levels of MDR *S* Typhi were estimated in Indonesia and the Philippines, with the prevalence remaining under 3% for all years (figure 1). In 2019, the prevalence of MDR decreased to 9·0% (6·3–12·5) in India; however, MDR *S* Typhi remained high at 83·2% (77·9–87·6) in Pakistan.

In sub-Saharan Africa, there were large increases in the prevalence of MDR *S* Typhi between 1990 and 2019. In 1990, less than 10% of infections were associated with MDR in most countries (32 [84%] of 38), but by 2005, a significant rise of MDR was seen in countries, including Nigeria (74·8% [95% UI 63·2–85·1]), Kenya (68·4% [52·7–80·4]), and Ghana (65·1% [46·2–80·7]). MDR *S* Typhi then spread throughout the region and by 2019, 37 (97%) of 38 countries had over 10% MDR, and 33 (87%) of 38 countries had over 20% MDR. The highest prevalences of MDR *S* Typhi in 2019 were estimated to be in Togo (95·1% [85·7–98·9]) and Cameroon (93·4% [78·1–98·6]; figure 1).

**Figure 3:**
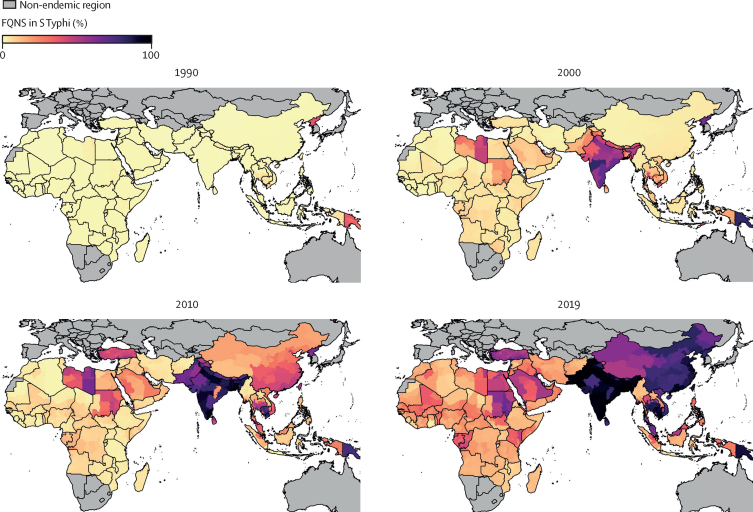
Model estimates of the prevalence of fluoroquinolone non-susceptibility in *Salmonella enterica* Typhi in all endemic countries for 1990, 2000, 2010, and 2019 at the administrative division level one resolution Results represent the mean of 1000 draws of the stacked ensemble plus the spatial temporal conditional autoregressive model. Estimates are presented for 75 endemic countries and were not produced for countries deemed non-endemic (shown in grey). Fluoroquinolone non-susceptibility was defined as ciprofloxacin minimum inhibitory concentration of ≥0·125 μg/mL or nalidixic acid resistance.

**Figure 4:**
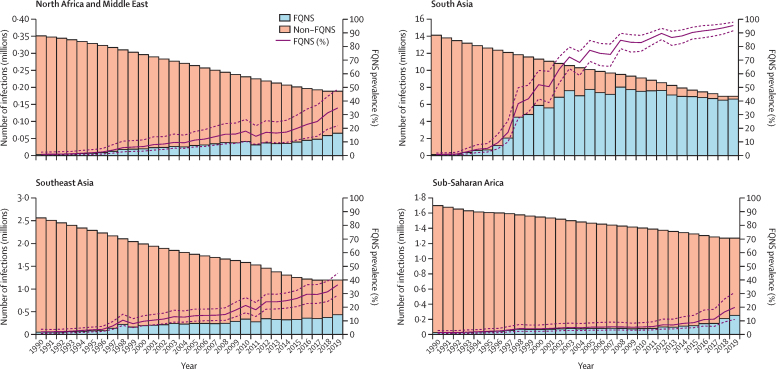
Model estimates of the number of FQNS and fluoroquinolone susceptible *Salmonella enterica* Typhi infections, and the prevalence of FQNS *S* Typhi isolates by GBD super-region and year Stacked bar plots show the number of *S* Typhi infections susceptible to fluoroquinolones (non-FQNS; orange) and the number of FQNS *S* Typhi infections (blue) for each year, 1990–2019. The prevalence of FQNS *S* Typhi infections is shown by the solid purple line, with uncertainty intervals shown by the purple dashed line. Estimates are shown for modelled endemic countries within each GBD super-region. FQNS=fluoroquinolone non-susceptibility. GBD=Global Burden of Diseases.

We estimate that virtually all *S* Typhi infections across the endemic area were susceptible to fluoroquinolones in 1990 with just 1·1% (95% UI 0·5–2·2; corresponding to 205 322 [71 483–452 201] infections) being FQNS (figure 3; table). However, the prevalence of FQNS increased rapidly in all regions throughout our study period, reaching 95·2% (91·4–97·7; 6·6 million [4·1–10·2] infections) in south Asia, 36·4% (28·7–44·8; 434 937 [278 341–661 753] infections) in southeast Asia, east Asia, and Oceania, 19·7% (10·9–30·6%; 249 612 [116 502–437 343] infections) in sub-Saharan Africa, and 35·0% (22·0–48·6; 65 954 [35 678–108 716]) in North Africa and the Middle East in 2019 (figure 4).

The prevalence of FQNS *S* Typhi started to increase in south Asia and southeast Asia in 1995. By 2019, the highest levels of FQNS *S* Typhi globally were estimated in Pakistan (99·1% [95% UI 98·1–99·7]), Bangladesh (97·9% [94·5–99·3]), India (94·1% [89·4–97·3]), Cambodia (91·7% [82·4–96·9]), and Nepal (89·8% [80·3–95·7]). There was high spatial variation in this region because FQNS remained lower in Indonesia (10·4% [3·4–22·8]) and Laos (19·6% [6·5–39·2]) in 2019. However, the uncertainty intervals were very wide for these estimates due to a paucity of input data (figure 3; appendix 1 p 12).

In sub-Saharan Africa, the increase in FQNS *S* Typhi prevalence occurred later than in Asia. FQNS *S* Typhi started to increase around 2000 and by 2019 had reached 43·7% (95% UI 15·3–74·9) in Equatorial Guinea, 41·6% (17·4–69·2) in the Republic of the Congo, 38·0% (15·6–63·1) in Benin, and 36·3% (18·1–58·8) in Kenya. Overall, resistance was lower than in Asia, but 24 (63%) of 38 countries in sub-Saharan Africa were still estimated to have over 15% FQNS *S* Typhi in 2019 (figure 3). The prevalence of third-generation cephalosporin-resistant *S* Typhi remained low across the whole endemic area over the entire study period, except for Pakistan, where our analysis shows the rapid emergence of third-generation cephalosporin-resistant *S* Typhi, which reached a prevalence of 61·0% (58·0–63·8) in 2019 (appendix 1 p 8; table).

**Figure 5:**
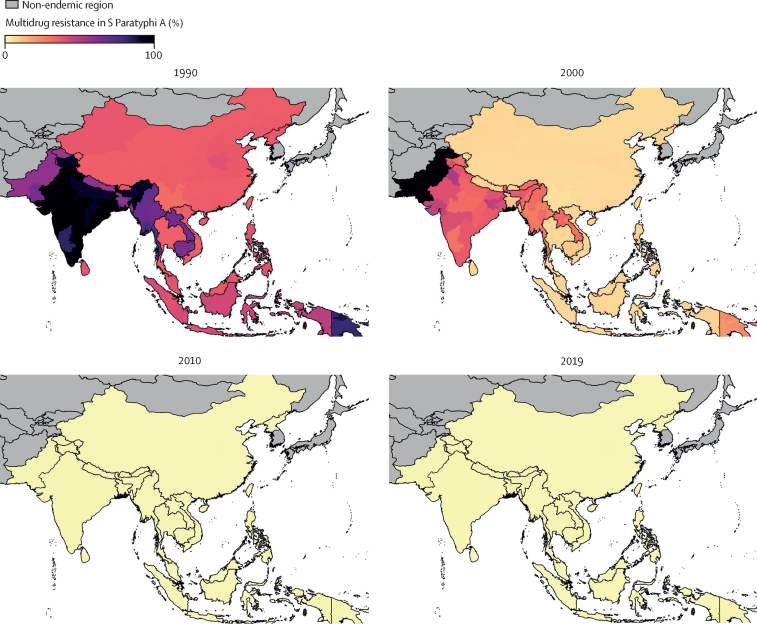
Model estimates of the prevalence of multidrug resistance in *S* Paratyphi A in all endemic countries for 1990, 2000, 2010, and 2019 at the administrative division level one resolution. Results represent the mean of 1000 draws of the stacked ensemble plus STCAR model. Estimates are presented for 18 endemic countries and were not produced for countries deemed non-endemic (shown in grey). Multidrug resistance was defined as concurrent resistance to ampicillin, chloramphenicol, and trimethoprim–sulfamethoxazole.

For *S* Paratyphi A, we estimated that the prevalence of MDR remained low in all countries across the study period, with slightly higher levels in the earlier years of the study. MDR was estimated at 9·2% (95% UI 2·4–24·7%; 603 278 [128 385–1·7 million]) in 1990 and 0·2% (0·0–0·4; 6122 [1364–16 429]) in 2019. MDR *S* Paratyphi A reached a high of 31·7% (15·6–51·8%) in Pakistan in 2002. However, this did not expand throughout the region and levels decreased in the following years (figure 5; appendix 1 p 8).

The prevalence of FQNS in *S* Paratyphi A was low in all endemic countries in 1990, with only 0·6% (95% UI 0·2–1·4; 40 250 [11 462–100 742]) *S* Paratyphi A infections estimated to be FQNS. The prevalence of FQNS *S* Paratyphi A varied greatly between countries; in 2019, the highest prevalences of FQNS *S* Paratyphi A were estimated in Pakistan (97·25% [96·1–98·5]) and Nepal (97·7% [96·6–98·6]), whereas estimates in Papua New Guinea remained low throughout the study period (2019: 1·4% [0·4–3·7]; appendix 1 p 9). Third-generation cephalosporin-resistant *S* Paratyphi A remained very low in all locations throughout the study period (appendix 1 p 10).

## Discussion

Our study provides the first detailed subnational estimates of the prevalence of antimicrobial resistant *S* Typhi and *S* Paratyphi A infections for 75 countries where enteric fever is endemic, from 1990 to 2019. Incorporating more than 600 different data sources, including a systematic review of the published literature, national surveillance reports, and individual patient microbiology datasets from population-based surveillance,^15^, ^16^, ^17^ this study is, to our knowledge, the largest and most comprehensive collation and analysis of phenotypic antimicrobial resistance data and trends in enteric fever, spanning three decades. The use of a spatiotemporal modelling framework, a relatively new approach in AMR research,^31^ allowed the examination of spatiotemporal trends and the prediction of AMR prevalence for locations without data. These results expand on earlier AMR enteric fever prevalence estimates produced for GBD 2019,^23^ refining the methodology and providing estimates for key AMR phenotypes over the past 30 years at higher spatial resolution.

The reduction in typhoid fever incidence rates^3^, combined with the development and global spread of AMR in *S* Typhi have been well described,^2^, ^3^, ^5^, ^32^ along with the emergence of *S* Paratyphi A as an important pathogen in Asia.^33^ Our study identified several trends in south Asia and southeast Asia, such as the rapid emergence of FQNS *S* Typhi isolates in the mid-1990s, and subsequent dominance which was driven by selective pressure exerted by the necessary shift to fluoroquinolone antibiotics,^4^, ^34^ and the consecutive decline of MDR *S* Typhi in this region, except in Pakistan where MDR remained high.

In sub-Saharan Africa, MDR *S* Typhi was virtually non-existent in 1990, but increased greatly from 2000 onwards, accounting for more than 50% of all *S* Typhi infections in 2019. In addition, from 2010, the prevalence of FQNS *S* Typhi showed steep increases in sub-Saharan Africa. Generally, the proportion of third-generation cephalosporin-resistant *S* Typhi remained low across the endemic area, with the worrying exception of extensively drug-resistant *S* Typhi in Pakistan, which emerged around 2016^18^ and reached more than 60% of all *S* Typhi infections nationally in 2019. Our findings are consistent with two genomic epidemiology studies, that examined the spread and international dissemination of AMR *S* Typhi genotypes.^18^, ^19^ For *S* Paratyphi A, the initially low levels of FQNS increased drastically (to >90% in 2019), mirroring the increased use of fluoroquinolones in endemic regions in Asia,^34^ whereas MDR and third-generation cephalosporin resistance were estimated to be low throughout the study period.

Our analysis on drug resistant enteric fever reflects the infectious divide between high-income countries and LMICs.^42^ Typhoid fever has been eliminated in high-income countries through investments in clean water and adequate sanitation at the beginning of the 20th century,^35^ and the disease is typically seen in travellers returning from endemic areas.^36^, ^37^ In LMICs, enteric fever contributes to the high overall infectious disease burden. Over many years, effective treatment has been the only countermeasure against typhoid fever,^2^, ^4^ whereas public health investments into clean water and sanitation have been neglected in countries with the highest typhoid fever burden.^38^, ^39^ The high incidence of typhoid fever in south Asia and southeast Asia, in combination with excess and often unregulated antibiotic use,^34^ created conditions that allowed for the emergence and subsequent dissemination of a dominant MDR *S* Typhi lineage H58 (renamed clade 4.3.1, along with its derived genotypes) in the 1990s.^32^ For currently unknown reasons, *S* Typhi H58 is more susceptible than other *S* Typhi genotypes to acquiring mutations or mobile genetic elements encoding resistance determinants and spread quickly across Asia and has been introduced into Africa on multiple occasions, replacing susceptible clades.^32^ Originally, MDR in *S* Typhi in Asia was encoded by a large IncHI1 plasmid,^32^, ^40^ which incurred a fitness cost to isolates.^41^ With the switch to the fluoroquinolones, these IncHI1 plasmids were no longer being maintained in *S* Typhi populations,^42^ with a corresponding reduction in the prevalence of MDR, as reflected in our estimates. However, MDR determinants were incorporated into the chromosome of later H58 lineages, which are now being commonly seen in east Africa and Pakistan,^32^, ^40^ with these features likely to be maintained in future bacterial generations. In Pakistan, the MDR *S* Typhi H58 lineage has acquired additional plasmids, conferring ceftriaxone resistance and fluoroquinolone resistance,^6^ transforming to extensively drug-resistant *S* Typhi, leaving only azithromycin and carbapenems as treatment options. These extensively drug-resistant *S* Typhi isolates have also been identified in travellers returning from Pakistan^43^ and case reports from India, indicating intercontinental spread.^41^

Consistent with whole genome sequencing studies that revealed the introduction of clades H58 from Asia into eastern sub-Saharan Africa,^32^ and the microevolution and spread of endemic clade H56 (renamed genotype 3.1.1) in western sub-Saharan Africa,^44^, ^45^ our estimates show the emergence of MDR *S* Typhi in western sub-Saharan Africa around 2000 and in eastern sub-Saharan Africa around 2005, and subsequent dominance of this MDR phenotype.

Due to its comprehensiveness and global approach, our analysis serves as a stark reminder of the scarce treatment options available for enteric fever. Although *S* Typhi has regained susceptibility to the former first-line drugs (eg, chloramphenicol, ampicillin, and co-trimoxazole) in several countries in south Asia, these options are not as efficacious.^46^, ^47^ Due to the high prevalence of MDR *S* Typhi in conjunction with increasing levels of FQNS, patients in sub-Saharan Africa might be unable to obtain or afford effective alternative treatments, such as ceftriaxone or azithromycin. We also highlight the rise of third-generation cephalosporin-resistant *S* Typhi in south Asia. This is particularly noteworthy in the context of Pakistan, where the persisting high prevalence of MDR suggests the emergence of extensively drug-resistant *S* Typhi isolates, but this has not been explicitly modelled.

Our analysis and estimates can serve as a tool to inform and guide the implementation of policies and programmes to combat AMR enteric fever. In 2018, typhoid conjugate vaccines that are safe and highly immunogenic in children younger than 2 years became available.^48^ Large, randomised trials in children (aged 9 months to 16 years) showed high vaccine efficacies (>80%) in endemic settings in Nepal, Bangladesh, and Malawi after 1 year of follow-up.^49^, ^50^, ^51^ WHO recommends typhoid conjugate vaccine for use in all countries where typhoid is endemic, prioritising countries with the highest burden of typhoid disease or antimicrobial resistance,^52^ and GAVI, the Vaccine Alliance, has endorsed funding of vaccine roll-out in eligible countries. In Pakistan, typhoid conjugate vaccines have been successfully deployed in outbreak settings to avert extensively drug-resistant *S* Typhi^53^ and have been integrated into routine childhood vaccination. However, there are no long-term efficacy data on typhoid conjugate vaccines, nor do they offer cross protection against *S* Paratyphi infections, which showed a disproportionate increase in many countries in south and southeast Asia over the past decade,^33^ which might continue as vaccination drives down the incidence of *S* Typhi.

A 2022 modelling study^54^ estimated that routine immunisation with typhoid conjugate vaccine at age 9 months and a catch-up campaign up to age 15 years could avert up to 67 million cases of typhoid fever in the 73 LMICs eligible for GAVI support over 10 years, reflecting a reduction between 46% and 74% of cases in individual countries, depending on age structure and vaccination coverage. In addition, the relative prevalence of AMR typhoid fever was predicted to decline by 16%.^54^ The estimates for AMR prevalence were based on the systematic review by Browne and colleagues,^5^ which was also a foundation of this study.

Preventing typhoid fever would lead to a decrease in antibiotic consumption that is associated with this illness. Studies estimate that between three and 25 patients are treated with antibiotics for suspected typhoid fever for each true (ie, blood culture confirmed) case,^48^ a consequence of the unavailability of sensitive and specific rapid tests^55^ and the absence of universal health care in high-burden countries, that incentivises self-medication (non-prescription use) with antibiotics as the cheapest treatment option.^56^ Such inappropriate antimicrobial use promotes the resistance evolution in bystander organisms, creating an important AMR gene pool that can be transferred to other pathogens and contribute to an increased burden of AMR.^57^

As a faeco-orally transmitted disease, control of enteric fever will ultimately depend on improvements in WASH. These are important building blocks of an integrated approach to reduce the incidence of infections, as mandated by the WHO Global Action Plan on Antimicrobial Resistance.^58^ The World Bank considers investments in WASH as AMR-sensitive development priorities and emphasises the interdependency between the Sustainable Development Goals (SDG) 6 (achieve universal and equitable access to safe and affordable drinking water and adequate sanitation and hygiene for all) and AMR.^59^ In keeping with our findings, the coverage for basic sanitation services was less than 50% in sub-Saharan Africa, India, Bangladesh, and Nepal in 2015. In sub-Saharan Africa, only 24% of the population (data only available for 6 countries) used safely managed drinking water (defined as on site, available when needed, and free from contamination), whereas insufficient data were available from India.^39^ The current pace of WASH progress is projected to be too slow to achieve the SDG targets set for 2030,^60^ which will not only negatively affect diarrhoeal diseases and AMR, but also gender equality and social justice.^61^

The main limitations of this study surround the paucity, heterogeneity, and timeliness of the input data. Data were particularly sparse in Oceania, parts of sub-Saharan Africa, and the Middle East; this is reflected by the large uncertainty intervals around our estimates in these regions. The data informing estimates of high third-generation cephalosporin resistance prevalence in Pakistan was obtained from high-quality studies done by the SEAP^26^ Consortium and Aga Khan University Hospitals (personal communication), and are supported by whole genome sequencing studies.^6^ However, only a small number of third-generation cephalosporin resistance datapoints were available, generating large uncertainty intervals. A potential limitation of our study is that in instances where AST results for one of the three defining antimicrobials of MDR were unavailable, we deduced the prevalence of MDR from the remaining two antimicrobials. This approach might have led to an overestimation of MDR. Another limitation was the sparsity of data from the later years of the study due to a dependency on published reports. The inherent delay between concluding research and its eventual publication contributed to this scarcity. However, studies such as STRAATA^15^, TSAP^17^ and SEAP^26^, and datasets from collaborating institutions, such as the Aga Khan University Hospitals in Pakistan, ensured that the data from these later years was of high-quality, limiting any potential negative effects on the models.

Our study identified relatively little subnational variation of AMR. Modelling at the administrative level one district spatial resolution allowed us to select suitable spatial covariates for the likely locations the infections identified in each hospital were acquired. However, the lack of data from diverse facilities within countries, together with the clonal nature of the pathogen, likely contributed to the limited subnational variation observed.

Although we harness a Bayesian spatial-temporal framework to help improve the estimation of the statistical uncertainty of model parameters and smooth estimates, and to help generate better predictions at locations without data, there is the possibility for this approach to under-smooth or over-smooth estimates or predictions. Thus, we cannot discount the possibility that estimates of prevalence in particular countries or districts without any data might have been overestimated when lending weight from observed high prevalence values in a neighbouring location, or vice versa when there are excessive low or zero prevalence values in a neighbouring country or district.

Another limitation is possible selection bias. AMR data were mostly collected through passive surveillance, which could lead to an over-representation of AMR.^5^, ^62^ In addition, when analysing resistance data over a 30-year period, we encountered the difficulty to deal with revised (ie, lowered) susceptibility breakpoints from the Clinical & Laboratory Standards Institute (CLSI). To allow the analysis of AMR trends from 1990 to 2019, FQNS *S* Typhi and *S* Paratyphi A was defined as ciprofloxacin non-susceptibility (based on CLSI 2019 breakpoints) or nalidixic acid resistance.^5^ However, this approach restricted the amount of data available as many studies did not specify the breakpoints used to interpret ciprofloxacin resistance.

Generally, the input data from the systematic review had insufficient information on the laboratory quality, microbiological methods used, sampling strategies implemented, and potential biases in the data.^5^ Our models aimed to decipher the spatial and temporal trends in AMR among noisy data; however, the effect of the high heterogeneity is reflected by the validation metrics of our models. The input data were primarily aggregate data and were scarce for alternative antibiotics. This meant that it was not possible to model the prevalence of concurrent MDR, FQNS, and third-generation cephalosporin resistance, or the emergence of azithromycin resistance.

Another caveat is the timeliness of data. Our analysis provides estimates from 1990 to 2019 in endemic countries, as there is very little up-to-date data of AMR *S* Typhi and *S* Paratyphi A available and sharable. Unfortunately, the most prominent global initiative for the surveillance of bacterial pathogens, the WHO Global Antimicrobial Surveillance System (GLASS),^63^, ^64^ which collates and publishes routinely collected AMR data annually (from 76 countries in 2021), reports only aggregated AMR data for *Salmonella species*. Therefore, we were not able to incorporate these data. It would be desirable for WHO GLASS to report AMR data for *S* Typhi and *S* Paratyphi A, as well as non-typhoidal *Salmonella* separately, to monitor progress.

This study provides comprehensive and detailed estimates of the prevalence of MDR, FQNS and third-generation cephalosporin-resistant *S* Typhi and *S* Paratyphi A in all 75 endemic countries, spanning the past 30 years. Our analysis highlights the worsening trends in AMR enteric fever, concerning the magnitude, geographical extent and scale of AMR, which includes the recent emergence of extensively drug-resistant *S* Typhi. The detailed spatial maps could also help countries to prioritise the introduction of typhoid conjugate vaccines into routine childhood immunisation schedules. A reduction of enteric fever incidence, or preferably elimination, would reduce the associated antibiotic use and have substantial health benefits. Lastly, this modelling framework and AMR benchmark has the potential to guide empirical treatment of enteric fever in settings, where microbiological facilities and AMR surveillance systems are scarce, and could be updated with real-time microbiology data if those became available.

Correspondence to: Dr Christiane Dolecek, Centre for Tropical Medicine and Global Health, Nuffield Department of Medicine, University of Oxford, Oxford OX3 7LG, UK **christiane.dolecek@ndm.ox.ac.uk**

## Data sharing

This study followed the Guidelines for Accurate and Transparent Health Estimates Reporting (GATHER). The source code for all analysis is available at: https://github.com/NDM-GRAM/typhi_paratyphi_model_prep_code and https://github.com/NDM-GRAM/typhi_paratyphi_modelling_code. Data visualisations, all input data available to share, and all modelled estimates are available at our institutional website: ndm.ac/salmonella-tpt.

## Declaration of interests

Competing interests of GRAM Typhoid Collaborators are listed in appendix 1 (p 84).
