# Supplementary material for: Estimating the subnational prevalence of antimicrobial resistant Salmonella enterica serovars Typhi and Paratyphi A infections in 75 endemic countries, 1990–2019: a modelling study
Source: Lancet Glob Health. 2024 Feb 14;12(3):e406–18. doi: 10.1016/S2214-109X(23)00585-5 (PMC10882211; doi:10.1016/S2214-109X(23)00585-5)
Supplement: Supplementary appendix 1 [file mmc1.pdf]

THE LANCET
Global Health

Supplementary appendix 1

This appendix formed part of the original submission and has been peer reviewed. We post it as supplied by the authors.

Supplement to: GRAM Typhoid Collaborators. Estimating the subnational prevalence of antimicrobial resistant *Salmonella enterica* serovars Typhi and Paratyphi A infections in 75 endemic countries, 1990–2019: a modelling study. *Lancet Glob Health* 2024; **12:** e406–18.

Supplementary Materials

Data acquisition

The data source for this study was a recently published systematic review.[1] An expansion of the systematic review was performed to include studies reporting antimicrobial susceptibility testing (AST) results for five or more isolates, and for isolates from sites other than blood culture (Browne *et al*.[1] included studies reporting on 10 or more blood culture isolates). Data were extracted following the same methodology as the initial systematic review.

Additionally, national and international surveillance reports were incorporated into the study, as well as original datasets, contributed by collaborators in the field.

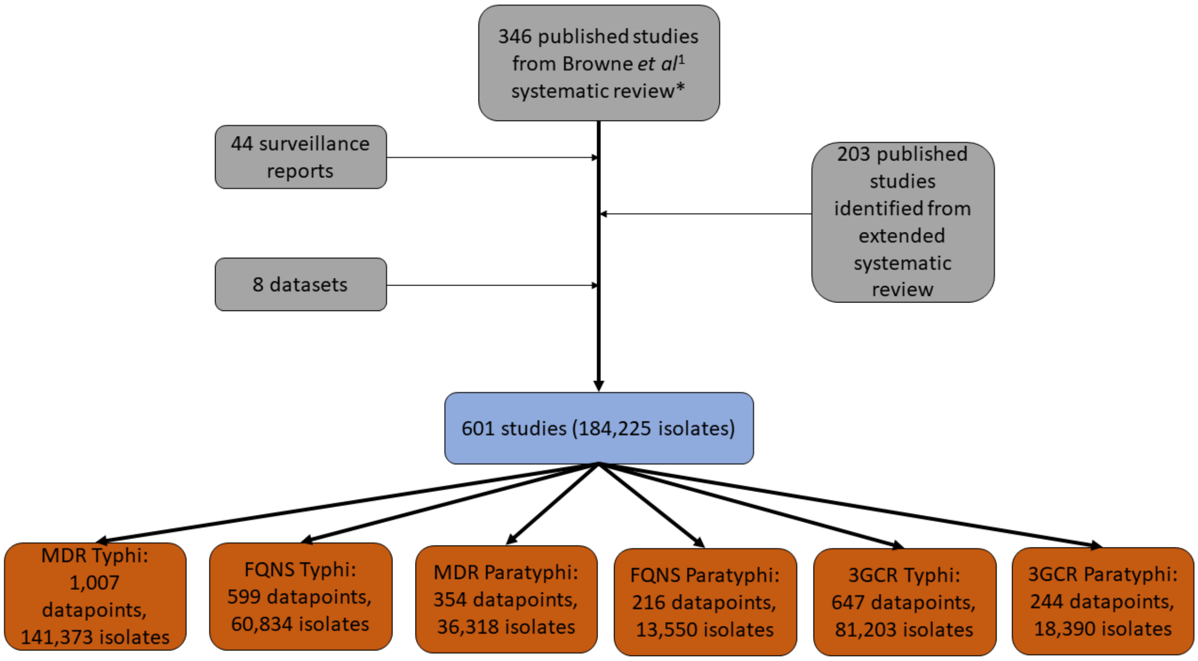

Figure S1. Flowchart indicating data sources. Flow chart showing the sources of the input data and the number of data points and isolates used for the models of multidrug resistance (MDR), fluoroquinolone non-susceptibility (FQNS) and third-generation cephalosporin resistance (3GCR) in *Salmonella* Typhi and Paratyphi A. *Two of the studies identified by the systematic review were replaced by line level datasets provided by the researchers, one study was deemed a duplicate, four were published in 1993 and did not provide study dates and were deemed to likely be from pre-1990, the remaining studies not included from the systematic review did not provide MDR or FQNS data for *S.* Typhi or *S.* Paratyphi A.[1]

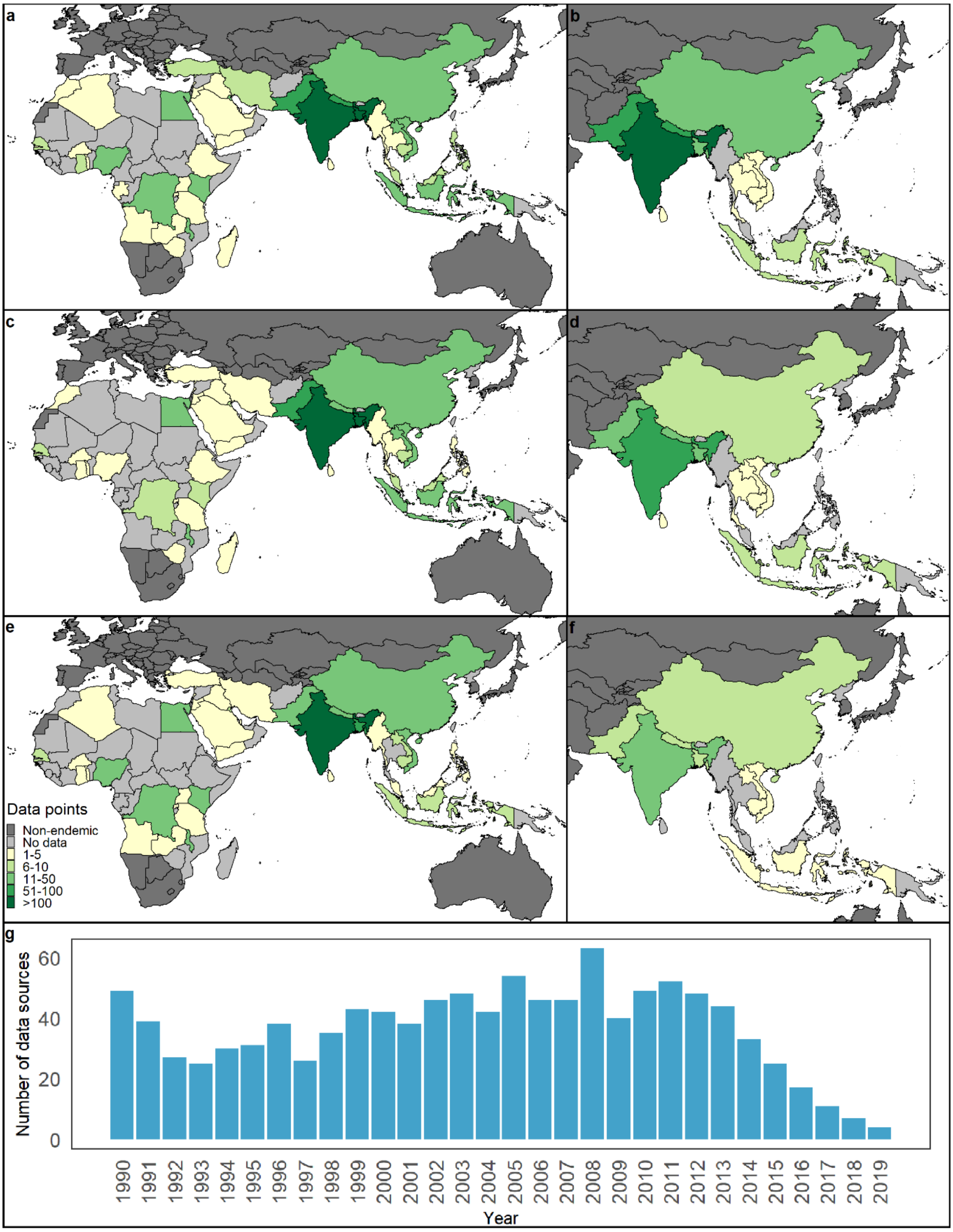

Figure S2. Model input data. Maps showing the number of input data points for each endemic country for all years 1990 to 2019, for a) MDR *S*. Typhi; b) MDR *S.* Paratyphi A; c) 3GCR *S.* Typhi; d) 3GCR *S.* Paratyphi A; e) FQNS *S.* Typhi; and f) FQNS *S.* Paratyphi A. Plot (g) shows the number of data sources per year of the study.

Inference of missing MDR values

As MDR is frequently encoded by a plasmid carrying resistance genes for ampicillin, chloramphenicol and co-trimoxazole (among others) it is was deemed reasonable to infer the prevalence of MDR based on the prevalence of resistance to the individual antibiotics if MDR was not reported. If the prevalence of MDR was not reported by a study but resistance to at least two of the three individual antibiotics was reported, than the lowest prevalence of resistance for ampicillin, chloramphenicol and co-trimoxazole was selected to be used as the MDR prevalence for that study. For example

- 20% chloramphenicol resistance, 18% ampicillin resistant = 18% MDR
- 90% ampicillin resistance, 92% co-trimoxazole resistance and 89% chloramphenicol resistance = 89% MDR
- 60% chloramphenicol resistance only = No MDR estimate inferred.

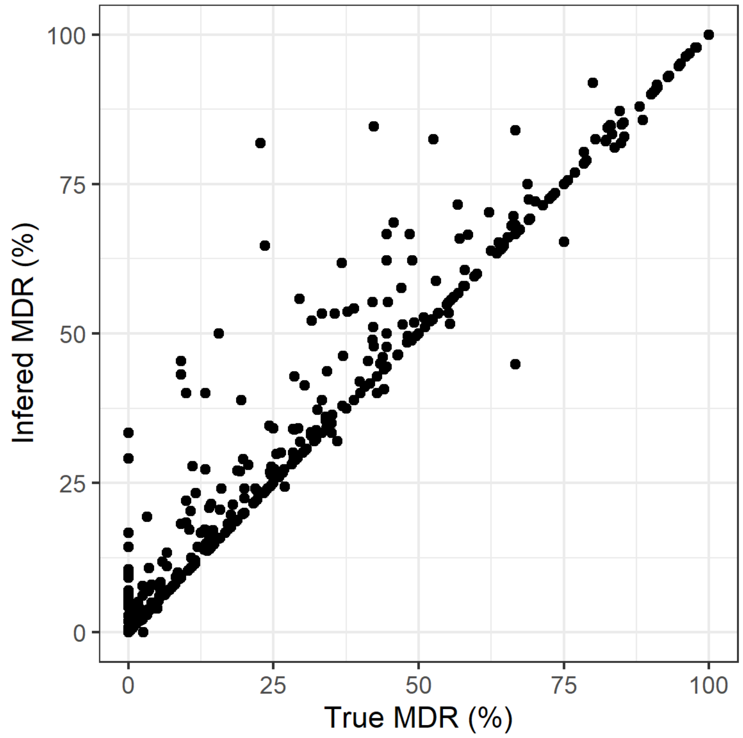

Figure S3. Comparison of observed versus inferred MDR. A scatter plot to compare the prevalence of MDR in studies reporting MDR and the prevalence which would be inferred using the described methods.

Outliering extreme values

Due to high heterogeneity in the input datasets we outliered datapoints found to have the most extreme values for the prevalence of resistance. To account for large spatial and temporal variations of the prevalence of resistance in the data two outliering methods were implemented. Firstly, we fit an initial generalised linear model (GLM) to the data and covariates to capture the crude spatial-temporal trends. Datapoints which lay outside of two times the median absolute deviation (MAD) of the input data from the modelled estimate for each country were assessed for classification as outliers. Secondly, 14 algorithms from the "DDoutlier" package in R were fit to the data using κ from a natural neighbour algorithm. Data points which were in the highest 95th percentile of scores from

two or more of these algorithms were assessed for classification as outliers. Outliers were then removed from the datasets prior to modelling.

Covariate selection

A range of spatial and temporally resolute covariates hypothesised to drive the models of MDR and FQNS in *S.* Typhi and *S.* Paratyphi A were selected from a variety of sources. Covariates available at the 5x5km resolution were aggregated to the admin 1 resolution, weighted by the population for each year from WorldPop. Covariates only available at the national level were considered using the same value for all admin 1 locations for that country-year.

Firstly, covariates were assessed for their linear association to the input data. Secondly a lasso penalised regression model was fit to the input data and covariates using the “glmnet” package in R. The covariates identified to be the most influential in the model, without increasing model complexity, were selected for consideration in the stacked ensemble model. Various covariates were considered for inclusion when fitting the stacked ensemble model and the final covariates selected were those which increased the predictive performance of the model, assessed using the five-fold cross validation statistics.

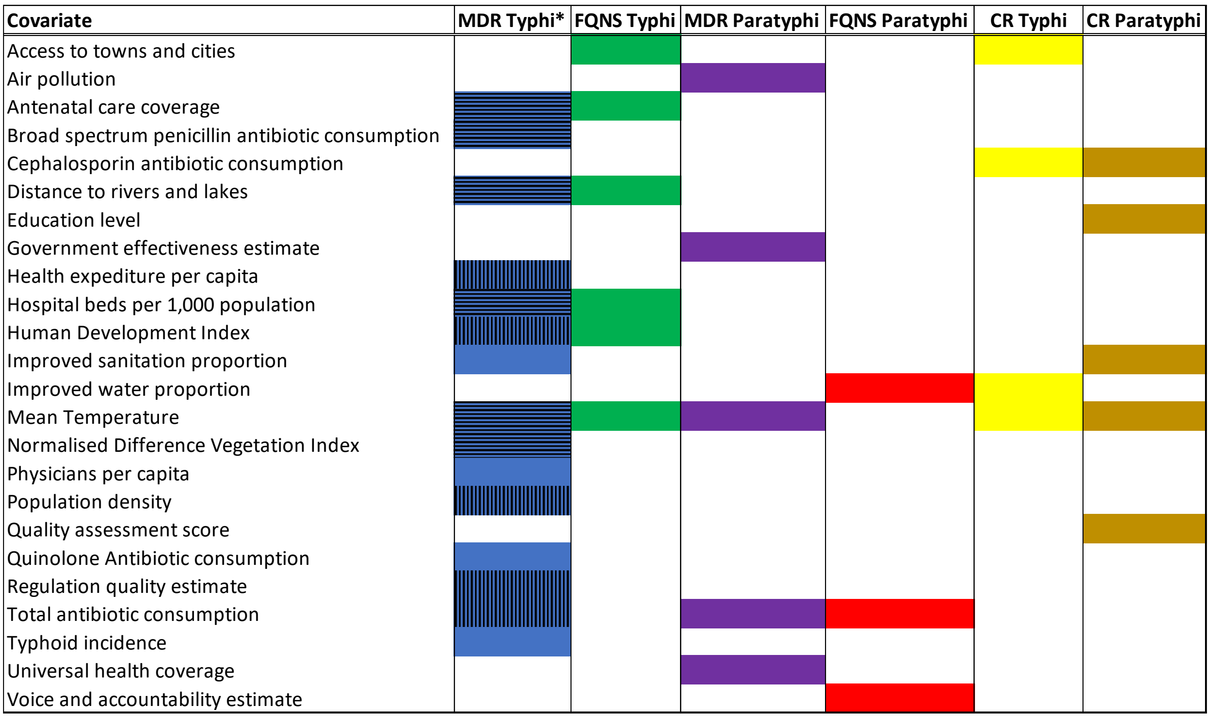

Figure S4. Covariate selection. For each model the covariates and child models used in the stacked ensemble model are shown by the coloured cells. *For the MDR *S.* Typhi the stacked ensemble model was fit separately for sub-Saharan Africa and all other regions; covariates and child models used in both models are shown in solid colour, those for just sub-Saharan Africa with vertical hatching and those for the other regions in horizontal hatching. For FQNS *S.* Typhi, GBD region was additionally included as a fixed effect.

Statistical model of antimicrobial resistance

For each of the models we considered the following child models for inclusion: boosted regression trees (BRT), generalised additive models (GAM), penalised regression models (elastic-net, ridge, lasso), random forest, cubist and neural-networks. Models were fit in R version 3·6·1, using the

packages 'CARET' version 6·085, 'mgcv' version 1·8·31 and 'glmnet' version 3·0·2. We fit the child models using five-fold cross validation for each indicator and selected the best performing, non-correlated child models based on the out-of-sample predictive performance (final covariates and child models for each indicator are shown in the Supplementary Materials). For MDR *S.* Typhi, models were fit separately for sub-Saharan Africa due to differing spatial trends and associations between covariates and data in these regions.

The out-of-sample predictions for each selected child model were taken and the weighted mean of these predictions, constraining the coefficients to sum to one, were then used to fit a Spatial-Temporal Conditional Autoregressive (ST-CAR) model.[2]

The ST-CAR model was fit using R-INLA[3], via the "R2Winbugs" package. An adjacency matrix was computed for the administrative units level 1 (admin 1) provinces based on the GAUL 2013 admin 1 shapefile from the FAO, with additional connections added for islands with limited neighbours. Random effects were included on the spatial and temporal terms, as well as a space-time interaction and a random effect on the source ID, and for the FQNS *S.* Typhi model on country. The models were fit with minimally informative priors on the hyperparameters. One thousand draws of the model posterior were taken and the mean and 95% uncertainty intervals were to assess the model estimates with uncertainty.

The admin 1 estimates were aggregated to the national level based on the population weighted mean prevalence of AMR. These were then aggregated to the GBD super region and global (consisting of all endemic countries analysed) level weighted by the *S.* Typhi or *S.* Paratyphi incidence.

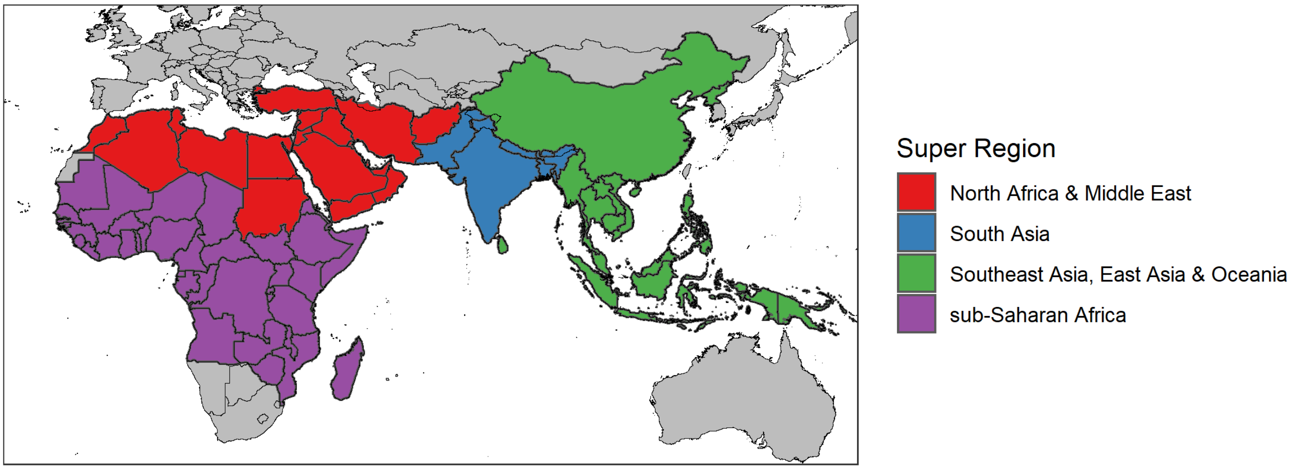

Figure S5. Countries modelled as endemic for *S.* Typhi and *S.* Paratyphi A. Endemicity was defined as locations with ≥10 cases per 100,000 population according to the Global Burden of Disease 2017 study estimates.[4] Island nations with no neighbours and populations less than two million were excluded from the analysis. *S.* Typhi was classified as endemic in all regions mapped, but *S.* Paratyphi A was only classified as endemic in the South Asia and Southeast Asia, East Asia & Oceania super-regions.

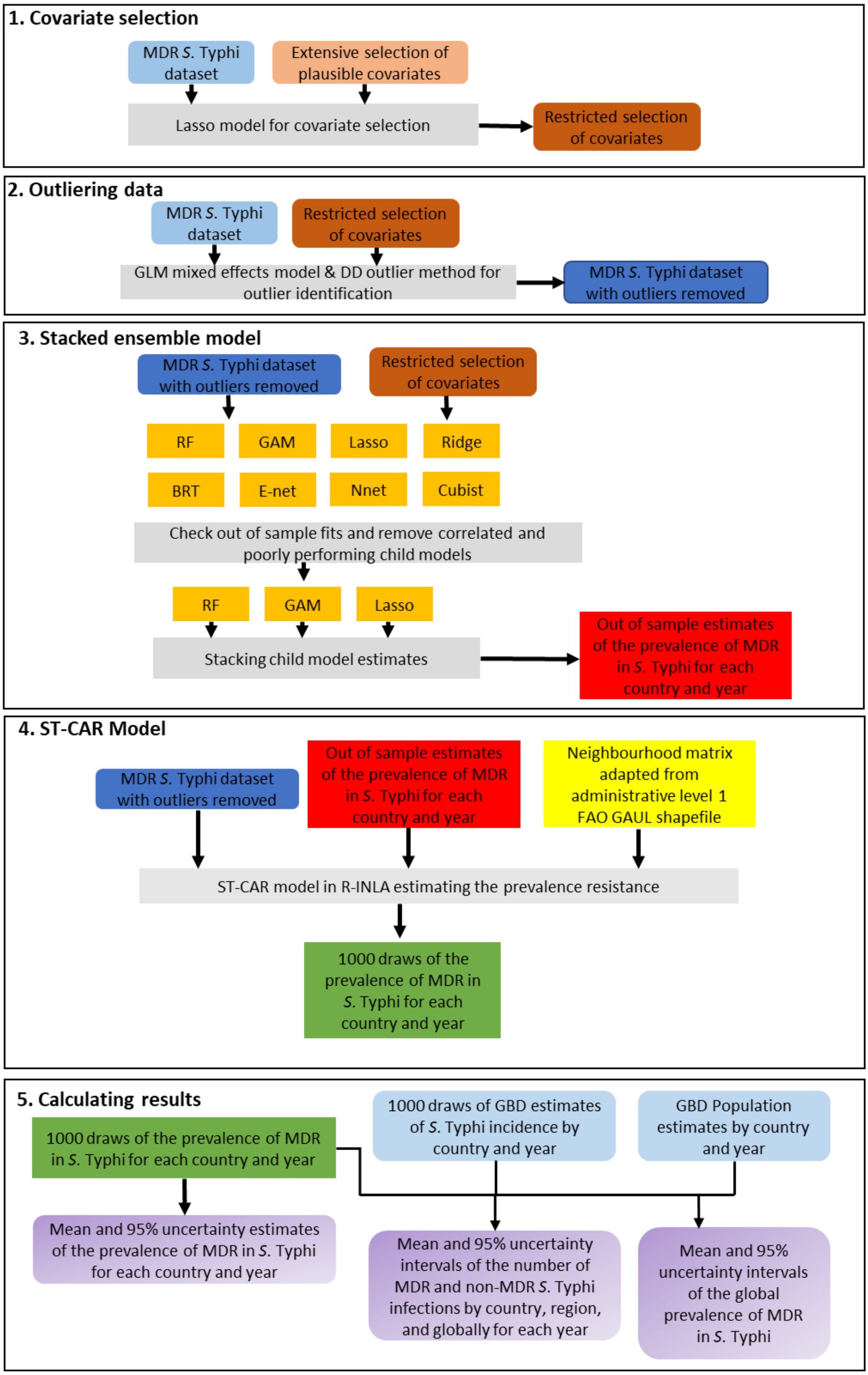

Figure S6: Schematic of the modelling strategy implemented for MDR and FQNS *S.* Typhi and *S.* Paratyphi A. This uses MDR *S.* Typhi as an example but the process was the same for all indicators.

Model validation

All models were validated using five-fold cross validation. Firstly, each data point was assigned to one of five random folds. The stacked ensemble model, followed by the ST-CAR model was fit holding out each fold of data, using the remaining data to estimate the prevalence of resistance for the held-out fold. The correlation (R^2) and root mean squared error (RMSE) between the input data and the out-of-sample estimates were calculated to assess the predictive validity of the model.

Global burden of bacterial antimicrobial resistance modelling

These estimates were produced by the authors as an extension to those used for estimating the global burden of antimicrobial resistance in Murray et al.[5] The authors have expanded on their previous work, refining the modelling framework to produce estimates for administrative level one locations in endemic countries for 30 years of the study.

The previous estimates used a Spatial-Temporal Gaussian Process Regression (ST-GPR) model to estimate national level prevalence of AMR in *S.* Typhi and *S.* Paratyphi A, utilising a location hierarchy to borrow strength from data points in countries deemed to be in similar epidemiological regions. Here, using an ST-CAR framework has enabled us to produce estimates at a higher spatial resolution, borrowing strength from data points in space based on a neighbourhood matrix of administrative units, opposed to imposing *a priori* assumptions of similar AMR patterns based a predefined location hierarchy. Whereas the Murray et al paper only details estimates of AMR for 2019, here we have produced estimates for 3 decades using an annual first-order random walk model to incorporate temporal patterns. Both models used a similar selection of covariates and used a stacked ensemble modelling framework in the first instance to incorporate covariates effects. Small differences in the covariates used have been implemented to fit models with the best predictive performance, and the models presented here use covariates at an administrative level one spatial resolution (where available), opposed to national level covariates used in the GBD models.

The expanded modelling framework has produced more detailed, robust estimates for a long period of time, greatly improving our understanding of the trends and patterns of AMR in *S.* Typhi and *S.* Paratyphi A for key antimicrobials. This enables the assessments of the impact of control and treatment strategies implemented for enteric fever and provides a benchmark from which to measure future change.

Supplementary Results

Supplementary Results - in the order as they appear in the manuscript

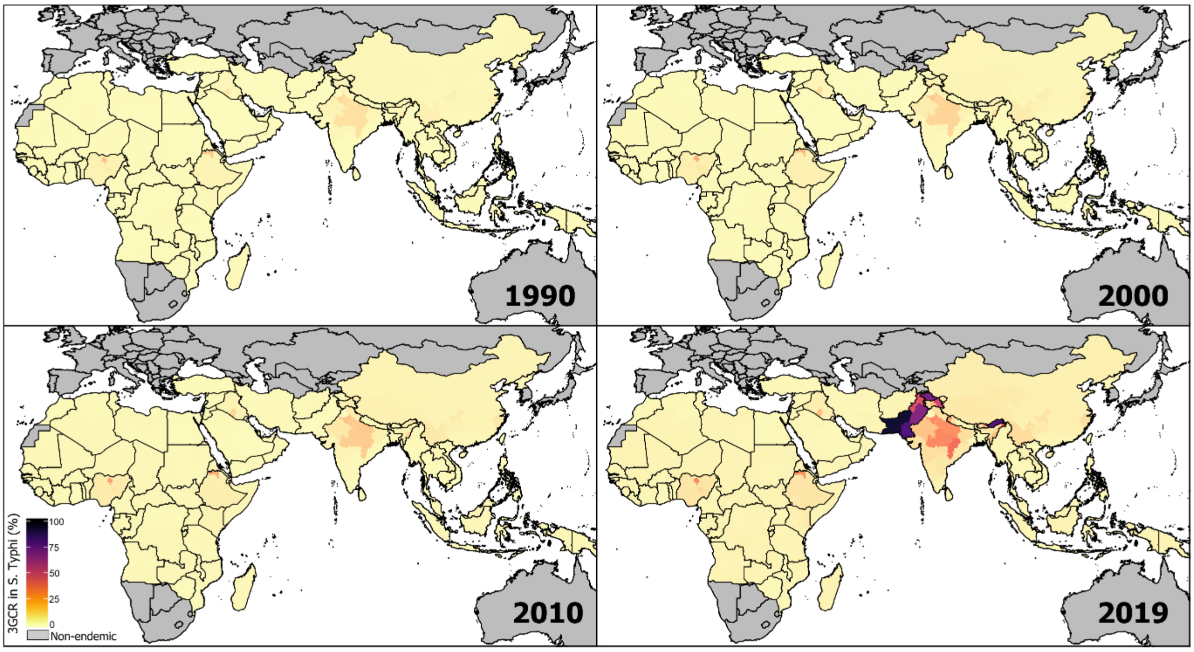

Figure S7. Model estimates for the prevalence of 3GCR in *S.* Typhi in all endemic countries for 1990, 2000, 2010 and 2019 at the administrative division level 1 resolution. Results represent the mean of 1000 draws of the stacked ensemble plus ST-CAR model. Estimates were not produced for countries deemed non-endemic (shown in grey).

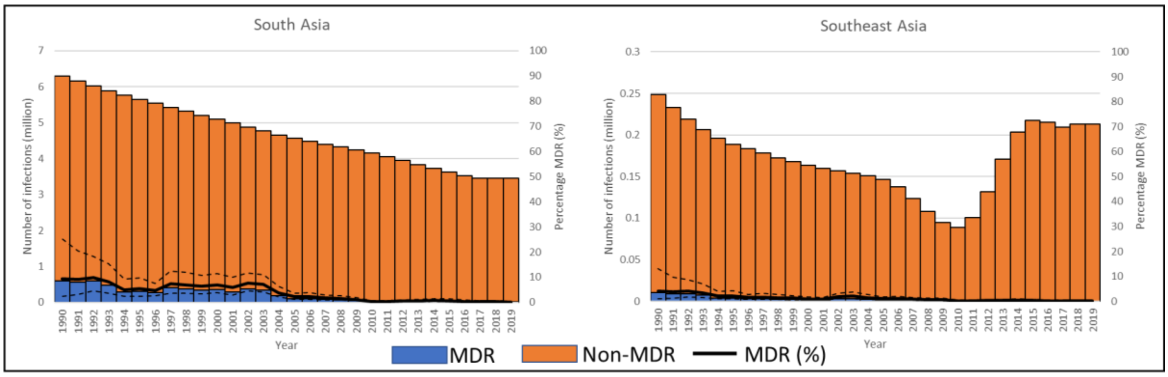

Figure S8. Model estimates of the number of MDR and non-MDR *S.* Paratyphi A infections, and the prevalence of MDR by year and GBD super-region. Stacked bar plots show the number of *S.* Paratyphi A infections susceptible to first-line antibiotics (non-MDR, orange) and the number of MDR *S.* Paratyphi A infections (blue) for each year, 1990-2019. The prevalence of MDR *S.* Paratyphi A infections is shown by the solid black line, with uncertainty intervals shown by the black dashed line. Estimates are shown for modelled endemic countries by each GBD super-region.

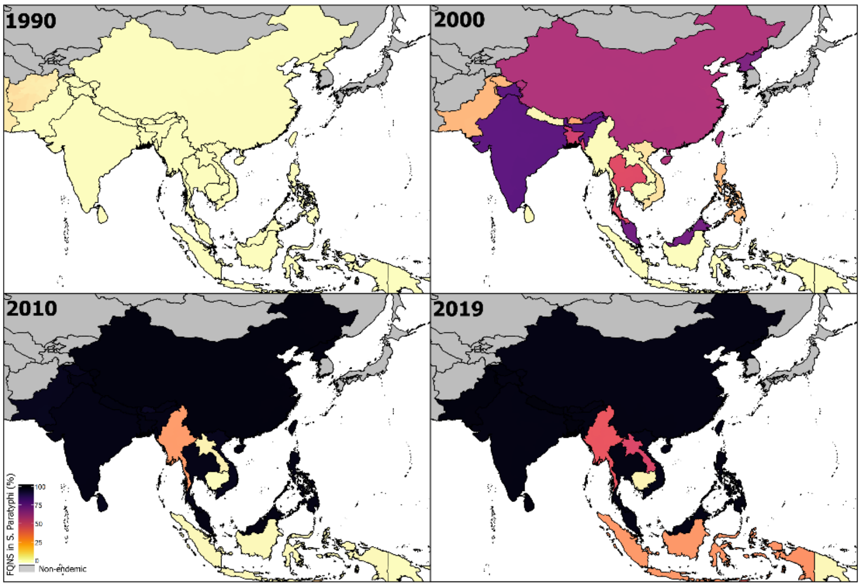

Figure S9. Model estimates for the prevalence (percentage) of fluoroquinolone non-susceptibility in *S.* Paratyphi A in all endemic countries for 1990, 2000, 2010 and 2019 at the administrative division level 1 resolution. Results represent the mean of 1000 draws of the stacked ensemble plus ST-CAR model. Estimates are presented for 18 endemic countries and were not produced for countries deemed non-endemic (shown in grey). Fluoroquinolone non-susceptibility was defined as either a MIC for ciprofloxacin of ≥0·125µg/mL or nalidixic acid resistance.

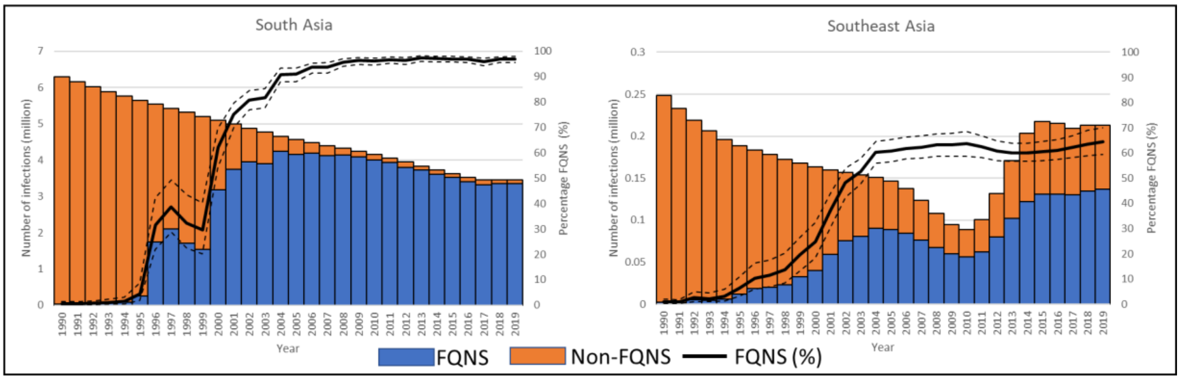

Figure S10. Model estimates of the number of FQNS and FQ-susceptible *S.* Paratyphi A infections, and the prevalence of FQNS by year and GBD super-region. Stacked bar plots show the number of *S.* Paratyphi A infections susceptible to fluoroquinolones (non-FQNS, orange) and the number of FQNS *S.* Paratyphi A infections (blue) for each year, 1990-2019. The prevalence of FQNS *S.* Paratyphi A infections is shown by the solid black line, with uncertainty intervals shown by the black dashed line. Estimates are shown for modelled endemic countries by each GBD super-region.

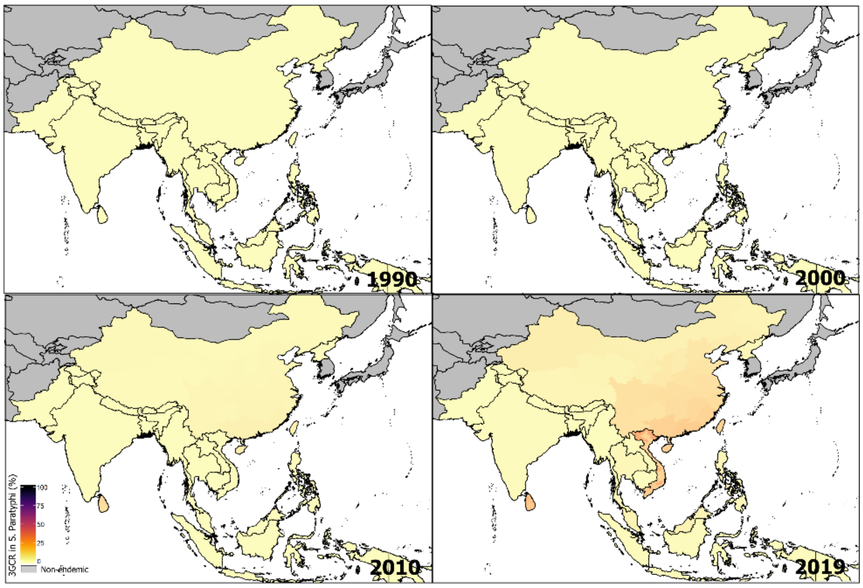

Figure S11. Model estimates for the prevalence of 3GCR in *S.* Paratyphi A in all endemic countries for 1990, 2000, 2010 and 2019 at the administrative division level 1 resolution. Results represent the mean of 1000 draws of the stacked ensemble plus ST-CAR model. Estimates were not produced for countries deemed non-endemic (shown in grey).

Supplementary Results - model uncertainty

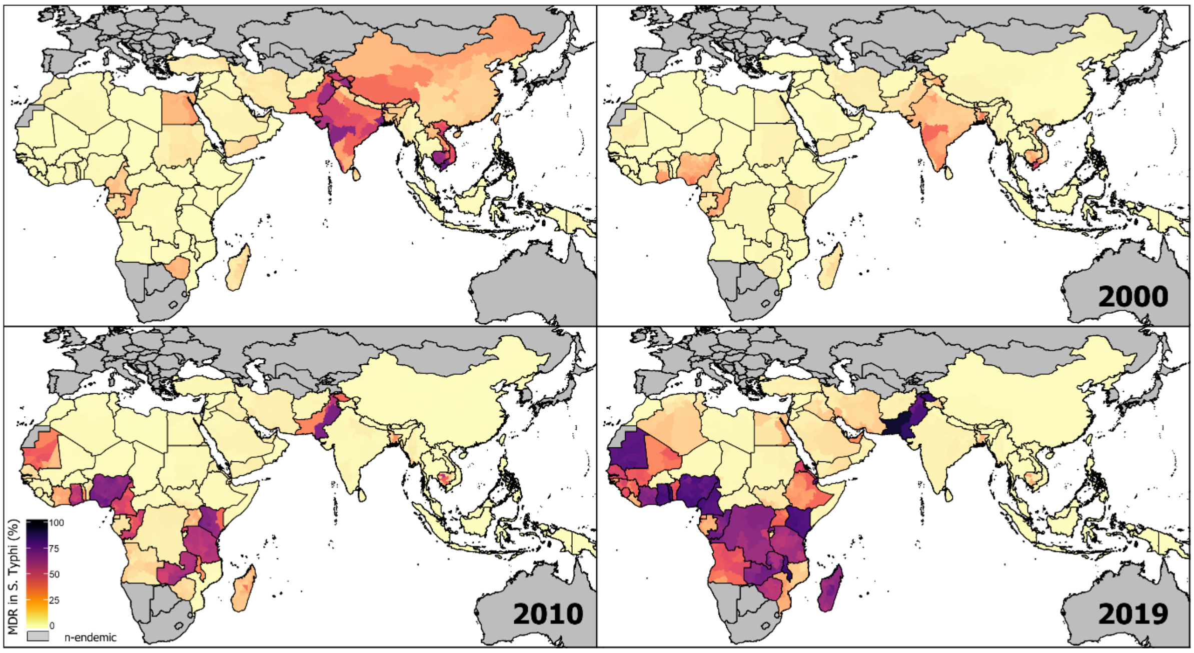

Figure S12. Prevalence of MDR in *S.* Typhi, model uncertainty – lower estimate. Modelled estimates of the prevalence of MDR in *S.* Typhi from the stacked ensemble plus ST-CAR model. The lower, 2.5th percentile, estimates are shown for the years 1990, 2000, 2010, 2019. Non-endemic countries are shown in grey.

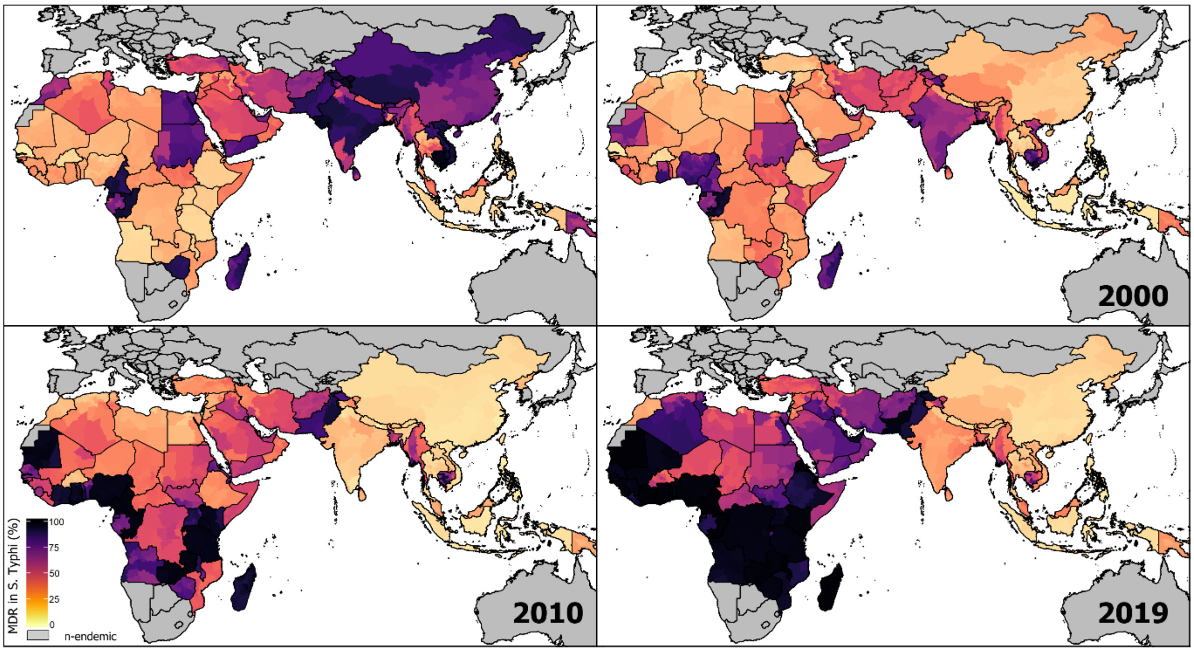

Figure S13. Prevalence of MDR in *S.* Typhi, model uncertainty – upper estimate. Modelled estimates of the prevalence of MDR in *S.* Typhi from the stacked ensemble plus ST-CAR model. The upper, 97.5th percentile, estimates are shown for the years 1990, 2000, 2010, 2019. Non-endemic countries are shown in grey.

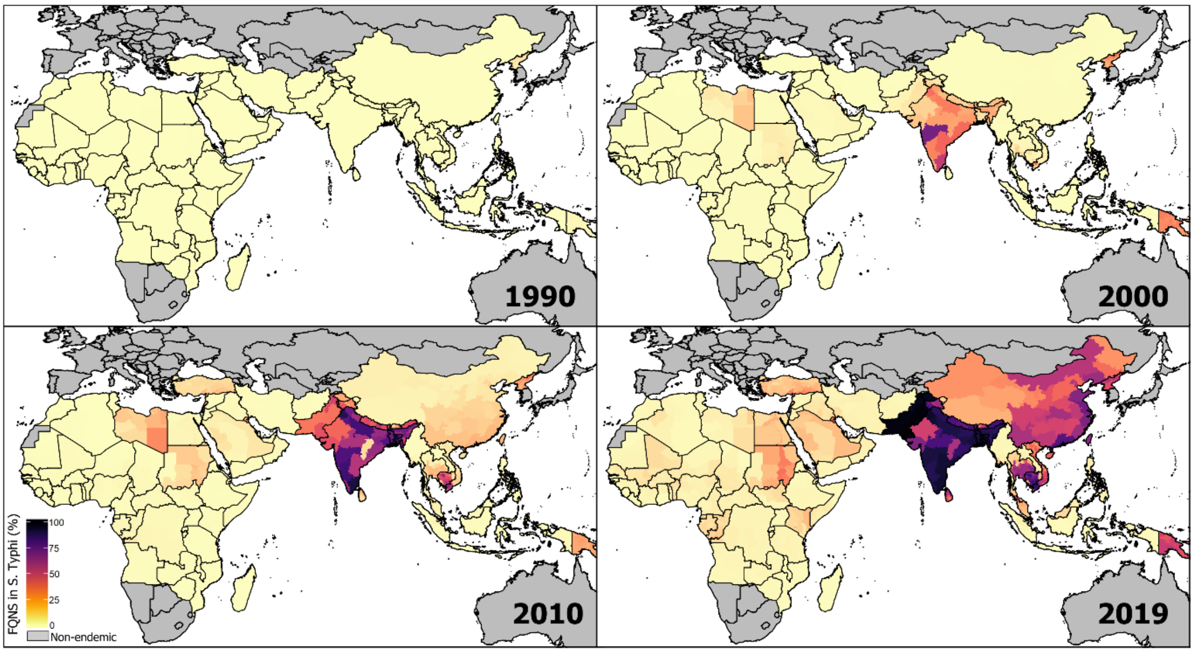

Figure S14. Prevalence of FQNS in *S.* Typhi, model uncertainty – lower estimate. Modelled estimates of the prevalence of FQNS in *S.* Typhi from the stacked ensemble plus ST-CAR model. The lower, 2.5th percentile, estimates are shown for the years 1990, 2000, 2010, 2019. Non-endemic countries are shown in grey.

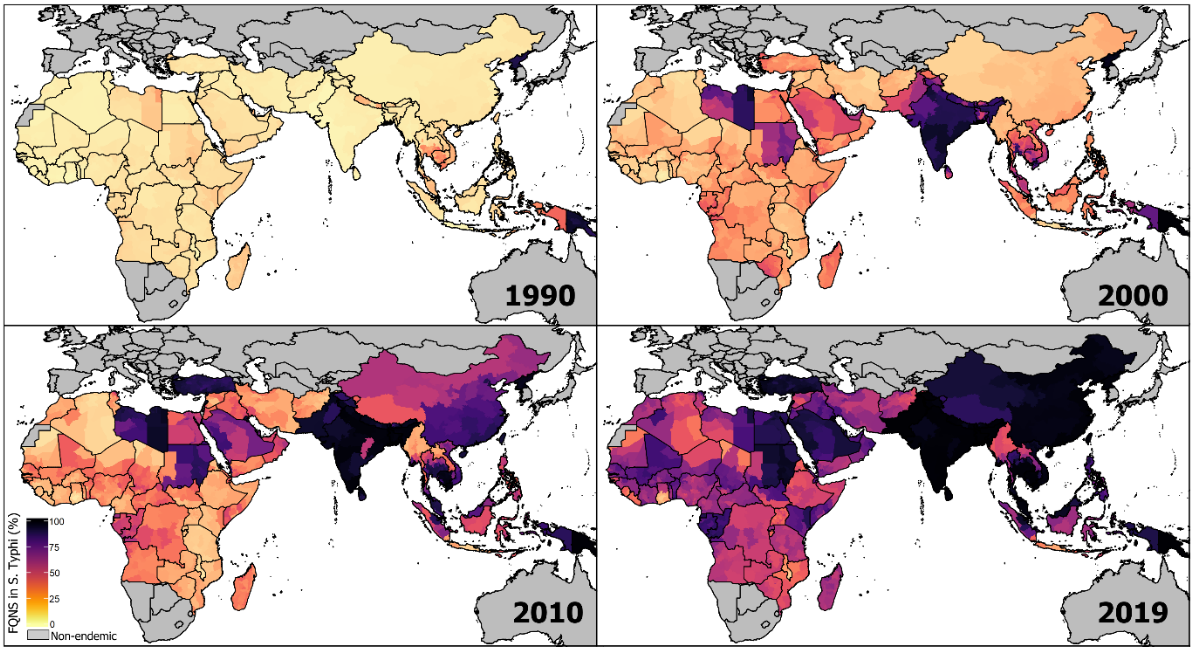

Figure S15. Prevalence of FQNS in *S.* Typhi, model uncertainty – upper estimate. Modelled estimates of the prevalence of FQNS in *S.* Typhi from the stacked ensemble plus ST-CAR model. The upper, 97.5th percentile, estimates are shown for the years 1990, 2000, 2010, 2019. Non-endemic countries are shown in grey.

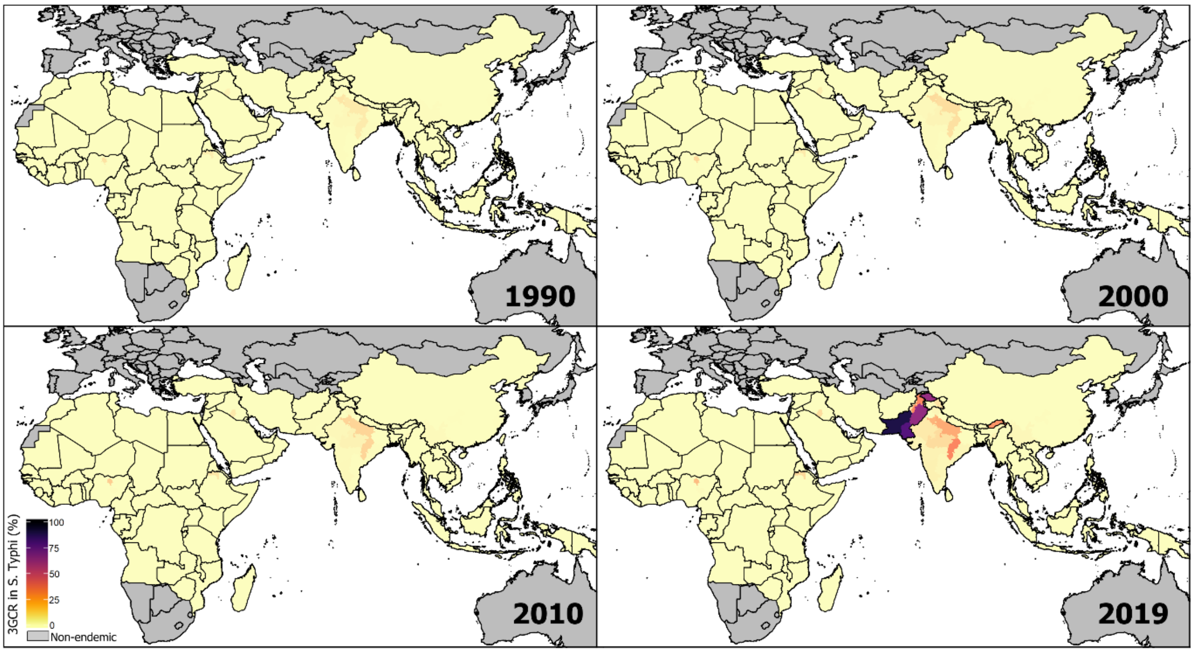

Figure S16. Prevalence of 3GCR in *S.* Typhi, model uncertainty – lower estimate. Modelled estimates of the prevalence of 3GCR in *S.* Typhi from the stacked ensemble plus ST-CAR model. The lower, 2.5th percentile, estimates are shown for the years 1990, 2000, 2010, 2019. Non-endemic countries are shown in grey.

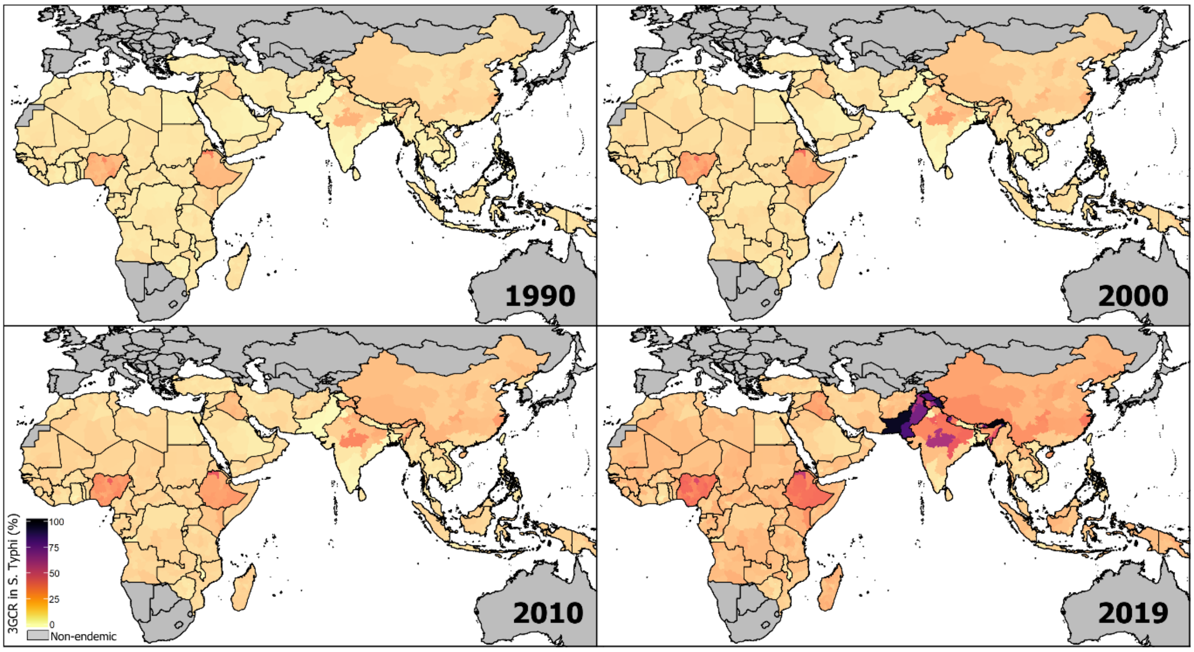

Figure S17. Prevalence of 3GCR in *S.* Typhi, model uncertainty – upper estimate. Modelled estimates of the prevalence of 3GCR in *S.* Typhi from the stacked ensemble plus ST-CAR model. The upper, 97.5th percentile, estimates are shown for the years 1990, 2000, 2010, 2019. Non-endemic countries are shown in grey.

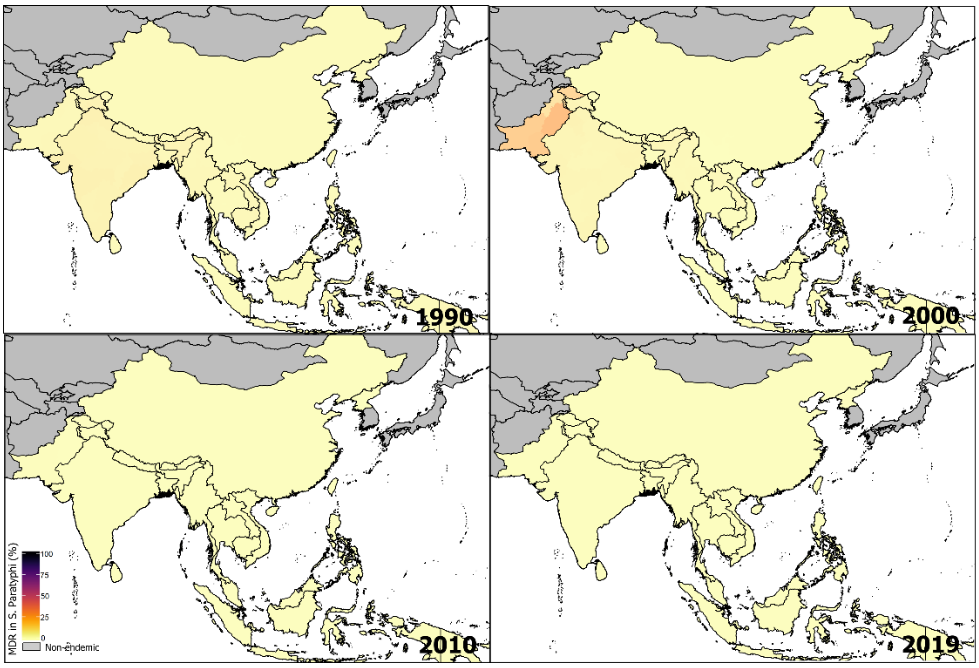

Figure S18. Prevalence of MDR in *S.* Paratyphi A, model uncertainty – lower estimate. Modelled estimates of the prevalence of MDR in *S.* Paratyphi A from the stacked ensemble plus ST-CAR model. The lower, 2.5th percentile, estimates are shown for the years 1990, 2000, 2010, 2019. Non-endemic countries are shown in grey.

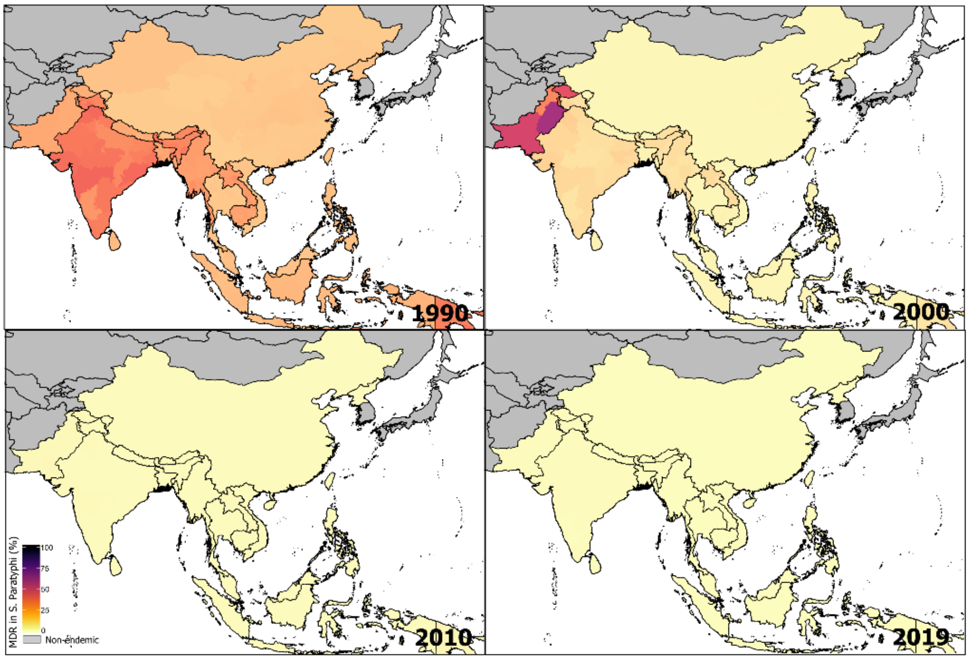

Figure S19. Prevalence of MDR in *S.* Paratyphi A, model uncertainty – upper estimate. Modelled estimates of the prevalence of MDR in *S.* Paratyphi A from the stacked ensemble plus ST-CAR model. The upper, 97.5th percentile, estimates are shown for the years 1990, 2000, 2010, 2019. Non-endemic countries are shown in grey.

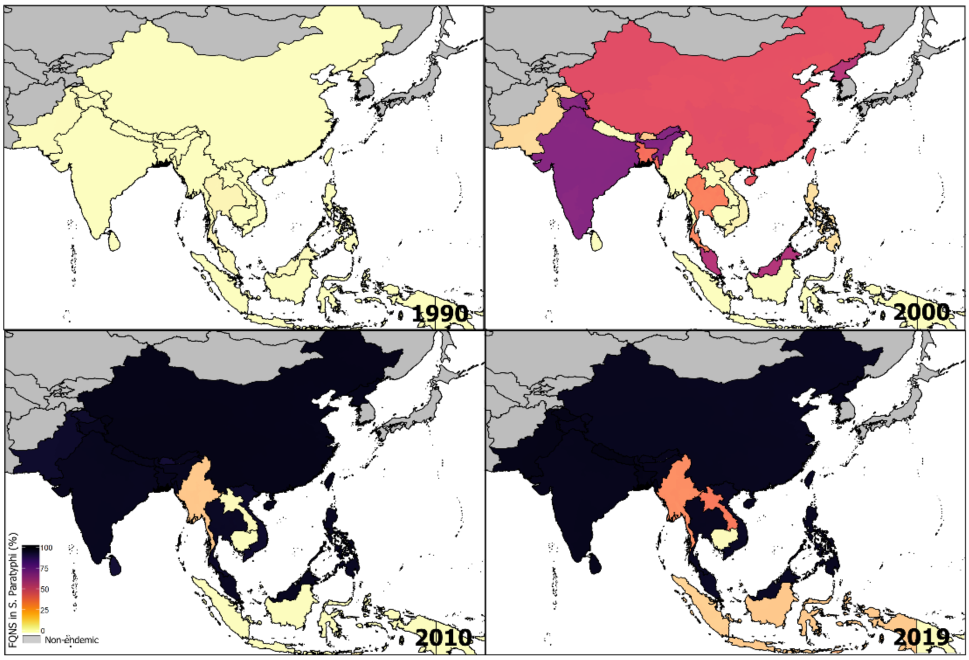

Figure S20. Prevalence of FQNS in *S.* Paratyphi A, model uncertainty – lower estimate. Modelled estimates of the prevalence of FQNS in *S.* Paratyphi A from the stacked ensemble plus ST-CAR model. The lower, 2.5th percentile, estimates are shown for the years 1990, 2000, 2010, 2019. Non-endemic countries are shown in grey.

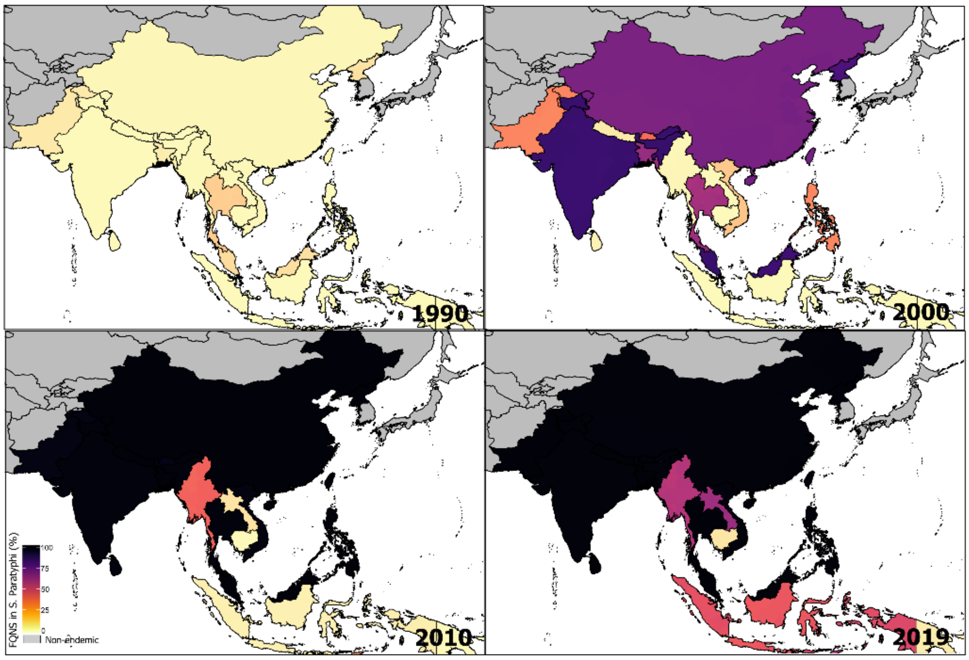

Figure S21. Prevalence of FQNS in *S.* Paratyphi A, model uncertainty – upper estimate. Modelled estimates of the prevalence of FQNS in *S.* Paratyphi A from the stacked ensemble plus ST-CAR model. The upper, 97.5th percentile, estimates are shown for the years 1990, 2000, 2010, 2019. Non-endemic countries are shown in grey.

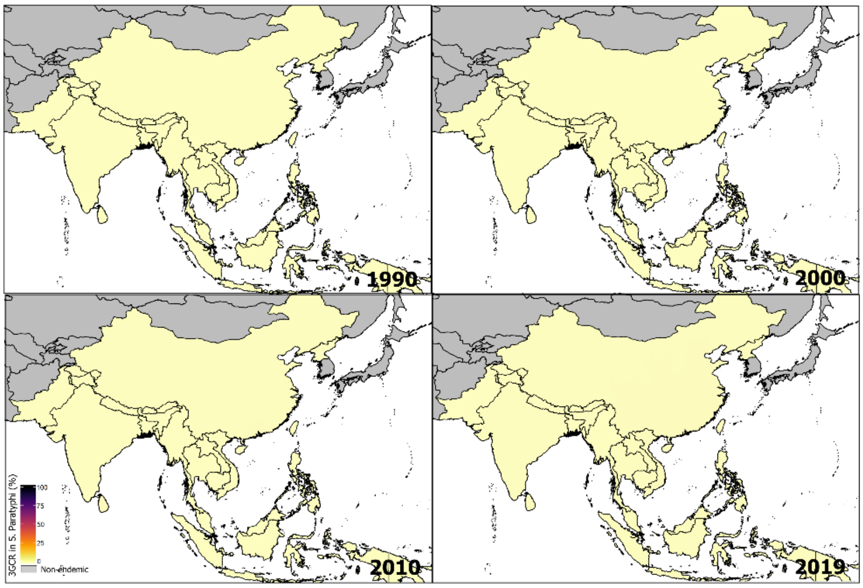

Figure S22. Prevalence of 3GCR in *S.* Paratyphi A, model uncertainty – lower estimate. Modelled estimates of the prevalence of 3GCR in *S.* Paratyphi from the stacked ensemble plus ST-CAR model. The lower, 2.5th percentile, estimates are shown for the years 1990, 2000, 2010, 2019. Non-endemic countries are shown in grey.

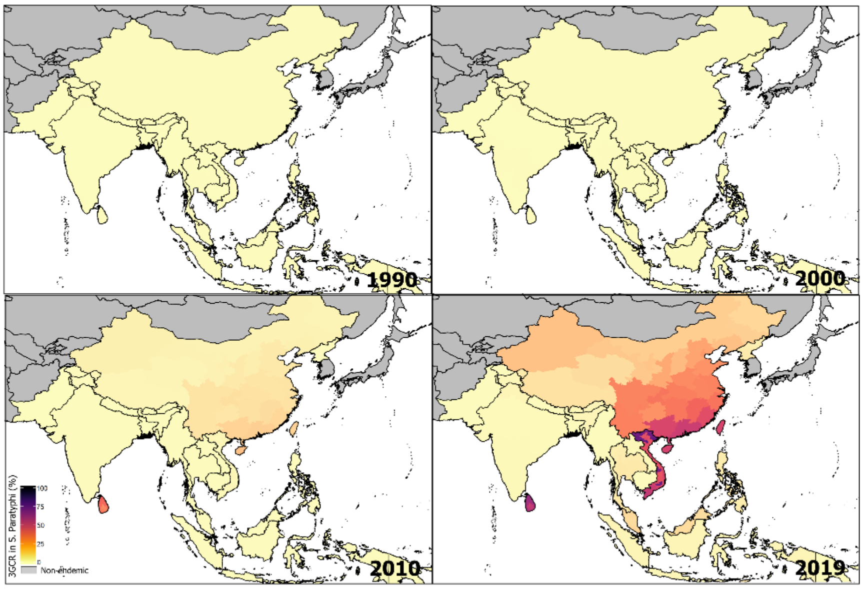

Figure S23. Prevalence of 3GCR in *S.* Paratyphi A, model uncertainty – upper estimate. Modelled estimates of the prevalence of 3GCR in *S.* Paratyphi from the stacked ensemble plus ST-CAR model. The upper, 97.5th percentile, estimates are shown for the years 1990, 2000, 2010, 2019. Non-endemic countries are shown in grey.

Drug-Pathogen	In Sample			Out of Sample (5-fold)		
	R^2	RMSE	Coverage (%)	R^2	RMSE	Coverage (%)
MDR Typhi	0·86	0·11	58	0·69	0·17	66
MDR Paratyphi	0·53	0·06	13	0·17	0·08	20
FQNS Typhi	0·84	0·16	55	0·78	0·18	64
FQNS Paratyphi	0·77	0·13	11	0·62	0·19	32
3GCR Typhi	0·79	0·04	5	0·55	0·06	7
3GCR Paratyphi	0·02	0·03	3	0·12	0·03	11

Table S1. Model validation statistics. In- and out-of-sample validation metrics of RMSE and R^2 for each of the six models.

Included data

Citation	Source	MDR Typhi	FQNS Typhi	MDR Paratyphi	FQNS Paratyphi	3GCR Typhi	3GCR Paratyphi
Aatekah O, Shazia S, Umber Z, Arjumand R, Zaidi AKM. Incidence of typhoid bacteremia in infants and young children in southern coastal Pakistan. Pediatr Infect Dis J 2010; 29(11): 1035-9.	Initial systematic review	✓				✓	
Abbas EHE, Albawab IM, Abdallah MZ, Al-Hassan R, Sahool EM, Bener A. Are new antibiotics essential in the treatment of *Salmonella* Typhi enteric fever? A comparison between enteric fever due to sensitive and resistant *Salmonella* in Ras Al-Kaimah, United Arab Emirates. Saudi Med J 1997; 18(2): 161-5.	Extended systematic review	✓					
Abdel Wahab MF, el-Gindy IM, Sultan Y, el-Naby HM. Comparative study on different recent diagnostic and therapeutic regimens in acute typhoid fever. J Egypt Public Health Assoc 1999; 74(1-2): 193-205.	Initial systematic review	✓					
Abdul H, Fizza K, Arshad MU. Detection of extended spectrum beta lactamases in typhoidal *Salmonella*e by phenotypic methods. African Journal of Microbiology Research 2014; 8(3): 313-5.	Extended systematic review					✓	✓
Abdullah FE, Faryal H, Kanwal F, Saboohi I, Iqbal MS. Enteric fever in Karachi: current antibiotic susceptibility of *Salmonella*e isolates. J Coll Physicians Surg Pak 2012; 22(3): 147-50.	Initial systematic review	✓		✓		✓	✓
Abdullah MA, Adnan Z, Sattar NY. Susceptibility of *Salmonella* enterica serotype Typhi, to the usual line of antimicrobial treatment in Rawalpindi. Pakistan Journal of Public Health 2013; 3(2): 14-8.	Initial systematic review	✓				✓	
Abdullahi B, Abdulfatai K, Wartu JR, Mzungu I, Muhammad HID, Abdulsalam AO. Antibiotics susceptibility patterns and characterization of clinical *Salmonella* serotypes in Katsina State, Nigeria. African Journal of Microbiology Research 2014; 8(9): 915-21.	Extended systematic review	✓	✓	✓	✓		
Abdullahi B, Olonitola OS, Jatau ED, Usman AD. Serological characterization and antimicrobial susceptibility patterns of clinical isolates of *Salmonella* from patients attending General Hospital, Funtua, Nigeria. Bayero Journal of Pure and Applied Sciences 2012; 5(1): 72-7.	Extended systematic review	✓					

Abdullahi M, Olonitola SO, Umoh VJ, Inabo IH. Antibacterial resistance profile and PCR detection of antibiotic resistance genes in *Salmonella* serovars isolated from blood samples of hospitalized subjects in Kano, North-West, Nigeria. Br Microbiol Res J 2015; 5(3): 245-56.	Initial systematic review	✓	✓	✓	✓		
Abdullahi M. Incidence and antimicrobial susceptibility pattern of *Salmonella* species in children attending some hospitals in Kano Metropolis, Kano State-Nigeria. Bayero Journal of Pure and Applied Sciences 2010; 3(1): 202-6.	Extended systematic review	✓	✓	✓	✓		
Abucejo PE, Capeding MR, Lupisan SP, et al. Blood culture confirmed typhoid fever in a provincial hospital in the Philippines. Southeast Asian J Trop Med Public Health 2001; 32(3): 531-6.	Initial systematic review	✓				✓	
Acharya D, Bhatta DR, Malla S, Dumre SP, Adhikari N, Kandel BP. *Salmonella* enterica serovar Paratyphi A: an emerging cause of febrile illness in Nepal. Nepal Med Coll J 2011; 13(2): 69-73.	Initial systematic review	✓	✓	✓	✓	✓	✓
Acharya D, Trakulsomboon S, Madhup SK, Korbsrisate S. Antibiotic susceptibility pattern and the indicator of decreased ciprofloxacin susceptibility of *Salmonella* enterica serovar Typhi isolated from Dhulikhel Hospital, Nepal. Jpn J Infect Dis 2012; 65(3): 264-7.	Initial systematic review	✓	✓			✓	
Acharya G, Butler T, Ho M, et al. Treatment of typhoid fever: randomized trial of a three-day course of ceftriaxone versus a fourteen-day course of chloramphenicol. Am J Trop Med Hyg 1995; 52(2): 162-5.	Initial systematic review					✓	✓
Acharya G, Davis T, Ho M, et al. Factors affecting the pharmacokinetics of parenteral chloramphenicol in enteric fever. The Journal of antimicrobial chemotherapy, 1997. http://onlinelibrary.wiley.com/o/cochrane/clcentral/articles/216/CN-00142216/frame.html (accessed.	Extended systematic review	✓					
Achla P, Grover SS, Bhatia R, Khare S. Sensitivity index of antimicrobial agents as a simple solution for multidrug resistance in *Salmonella* Typhi. Indian J Med Res 2005; 121(3): 185-93.	Initial systematic review	✓	✓				
Acquah SE, Quaye L, Sagoe K, Ziem JB, Bromberger PI, Amponsem AA. Susceptibility of bacterial etiological agents to commonly-used antimicrobial agents in children with sepsis at the Tamale Teaching Hospital. BMC Infect Dis 2013; 13: 89.	Extended systematic review					✓	
Adeshina GO, Osuagwu NO, Okeke CLE, Ehinmidu JO, Bolaji RO. Prevalence and susceptibility of *Salmonella* Typhi and *Salmonella* Paratyphi in Zaria, Nigeria. International Journal of Health Research 2009; 2(4): 355-60.	Initial systematic review	✓	✓	✓	✓		

Adhikari D, Acharya D, Shrestha P, Amatya R. Ciprofloxacin susceptibility of *Salmonella* enteric serovar Typhi and Paratyphi A from blood samples of suspected enteric fever patients. International Journal of Infection and Microbiology 2012; 1(1): 9-13.	Initial systematic review	✓	✓	✓	✓	✓	✓
Afia Z, Ibrahim NG, Tanwir A, Zohair A, Zaidi A, Rumina H. Nalidixic acid screening test in detection of decreased fluoroquinolone susceptibility in *Salmonella* Typhi isolated from blood. J Coll Physicians Surg Pak 2005; 15(7): 413-7.	Initial systematic review		✓				
Afifi S, Earhart K, Azab MA, et al. Hospital-based surveillance for acute febrile illness in Egypt: a focus on community-acquired bloodstream infections. Am J Trop Med Hyg 2005; 73(2): 392-9.	Initial systematic review	✓				✓	
Afroze SR, Rahim MA, Hasan MM, et al. Pattern of antibiotic sensitivity in enteric fever: A tertiary care hospital experience. Journal of Medicine (Bangladesh) 2014; 15(2): 122-4.	Initial systematic review	✓	✓	✓	✓	✓	✓
Afzal A, Sarwar Y, Ali A, et al. Molecular evaluation of drug resistance in clinical isolates of *Salmonella* enterica serovar Typhi from Pakistan. J Infect Dev Ctries 2013; 7(12): 929-40.	Initial systematic review	✓	✓			✓	
Afzal A, Sarwar Y, Ali A, Haque A. Current status of fluoroquinolone and cephalosporin resistance in *Salmonella* enterica serovar Typhi isolates from Faisalabad, Pakistan. Pak J Med Sci 2012; 28(4).	Extended systematic review		✓			✓	
Agada GOA, Abdullahi IO, Aminu M, et al. Prevalence and antibiotic resistance profile of *Salmonella* isolates from commercial poultry and poultry farm-handlers in Jos, Plateau State, Nigeria. Br Microbiol Res J 2014; 4(4): 462-79.	Extended systematic review	✓				✓	
Agarwal S, Madhu SV, Guleria JS, Talwar V. The problem of emerging chloramphenicol resistance in typhoid fever - A preliminary report. J Assoc Physicians India 1991; 39(6): 443-4.	Initial systematic review	✓					
Agarwal V, Jalgaonkar PD, Pathak AA, Saoji AM. An outbreak of multidrug resistant typhoid fever in Nagpur. J Assoc Physicians India 1992; 40(6): 416.	Extended systematic review	✓					
Aggarwal A, Ghosh A, Gomber S, Mitra M, Parikh AO. Efficacy and safety of azithromycin for uncomplicated typhoid fever: an open label non-comparative study. Indian Pediatr 2011; 48(7): 553-6.	Initial systematic review	✓	✓			✓	

Aggarwal A, Vij AS, Oberoi A. A three-year retrospective study on the prevalence, drug susceptibility pattern, and phage types of *Salmonella* enterica subspecies Typhi and Paratyphi in Christian Medical College and Hospital, Ludhiana, Punjab. J Indian Acad Clin Med 2007; 8(1): 32-5.	Initial systematic review	✓		✓		✓	✓
Agrawal P, Kapila K, Kumar S, Ghosh AN, Maurya AK. Novel restriction enzyme SSiI for the detection of mutation in GyrA gene of *Salmonella* enterica serovar Typhi. Indian J Pathol Microbiol 2010; 53(3): 509-12.	Extended systematic review	✓	✓			✓	
Agrawal V, Thakar AK, Kurhade AK, Pathak AA, Saoji AM. Plasmid mediated resistance to chloramphenicol, ampicillin, sulphame thoxazole and trimethoprim in *Salmonella* Typhi. Indian J Pathol Microbiol 1995; 38(2): 223.	Extended systematic review	✓					
Agyekum A. Prevalence of bacteria causing bacteraemia in children under five years in Agogo, Asante-Akyem and their antimicrobial susceptibility patterns: Kwame Nkrumha University of Science & Technology, Kumasi; 2010.	Initial systematic review	✓				✓	
Ahmed D, Nahid MA, Sami AB, et al. Bacterial etiology of bloodstream infections and antimicrobial resistance in Dhaka, Bangladesh, 2005-2014. Antimicrob Resist Infect Control 2017; 6.	Initial systematic review		✓	✓	✓	✓	✓
Akan O, Kanra G, Secmeer G, Ceyhan M, Ecevit Z, Berkman E. Antibiotic susceptibilities of *Salmonella* serogroups isolated from Turkish children. Turk J Pediatr 1997; 39(1): 7-11.	Extended systematic review	✓				✓	
Akinyemi KO, Bamiro BS, Coker AO. Salmonellosis in Lagos, Nigeria: incidence of Plasmodium falciparum-associated co-infection, patterns of antimicrobial resistance, and emergence of reduced susceptibility to fluoroquinolones. J Health Popul Nutr 2007; 25(3): 351-8.	Extended systematic review	✓	✓	✓	✓		
Akinyemi KO, Coker AO, Olukoya DK, Oyefolu AO, Amorighoye EP, Omonigbehin EO. Prevalence of multi-drug resistant *Salmonella* Typhi among clinically diagnosed typhoid fever patients in Lagos, Nigeria. Z Naturforsch [C] 2000; 55(5-6): 489-93.	Initial systematic review	✓	✓	✓	✓		
Akinyemi KO, Oyefolu AOB, Mutiu WB, et al. Typhoid fever: tracking the trend in Nigeria. (Special Issue: Tackling typhoid - what do global and country trends teach us?).American Journal of Tropical Medicine and Hygiene 2018;99(3 Suppl):41-47	Initial systematic review	✓	✓		✓	✓	
Akinyemi KO, Smith SI, Bola Oyefolu AO, Coker AO. Multidrug resistance in *Salmonella* enterica serovar Typhi isolated from patients with typhoid fever complications in Lagos, Nigeria. Public Health 2005; 119(4): 321-7.	Initial systematic review	✓	✓				

Akinyemi KO, Smith SI, Oyefolu AO, Fasure KA, Coker AO. Trends of multiple drug resistance in *Salmonella* enterica serovar Typhi in Lagos, Nigeria. East and Central African Journal of Surgery 2007; 12(1): 83-8.	Extended systematic review	✓	✓				
Al-Abbasy AJ. Molecular study of antibiotic resistance gene in *Salmonella* enterica serovar Typhi isolates.International Journal of Pharmaceutical Research 2018;10(3):378-384	Initial systematic review		✓			✓	
Alam A. Pola resistensi *Salmonella* enterica Serotipe Typhi, departemen Ilmu Kesehatan anak RSHS, Tahun 2006–2010. Sari Pediatri 2016; 12(5): 296-301.	Extended systematic review	✓				✓	
Alam MS, Pillai PK, Kapur P, Pillai KK. Resistant patterns of bacteria isolated from bloodstream infections at a university hospital in Delhi. J Pharm Bioallied Sci 2011; 3(4): 525-30.	Initial systematic review	✓					
Alaullah S, Bhuiyan MS, Farhana K, et al. *Salmonella* enterica serovar Typhi-specific immunoglobulin A antibody responses in plasma and antibody in lymphocyte supernatant specimens in Bangladeshi patients with suspected typhoid fever. Clin Vaccine Immunol 2009; 16(11): 1587-94.	Initial systematic review		✓			✓	
Albert MJ, Haider K, Nahar S, Kibriya AKMG, Hossain MA. Multiresistant *Salmonella* Typhi in Bangladesh [4]. J Antimicrob Chemother 1991; 27(4): 554-5.	Extended systematic review	✓					
Ale Z, Naqvi BS, Bushra R, Ali SQ. Comparative study on resistance pattern of different pathogens against cefixime and cefepime. Jordan Journal of Pharmaceutical Sciences 2010; 3(2): 145-56.	Extended systematic review					✓	
Ali A, Ali HA, Shah FH, Zahid A, Aslam H, Javed B. Pattern of antimicrobial drug resistance of *Salmonella* Typhi and Paratyphi a in a teaching hospital in Islamabad. J Pak Med Assoc 2017; 67(3): 375-9.	Initial systematic review	✓	✓	✓	✓	✓	✓
Ali MK, Sultana S. Antimicrobial sensitivity patterns of *Salmonella* Typhi in children. Bangladesh J Med Sci 2016; 15(3): 416-8.	Initial systematic review	✓	✓			✓	
Aliya N, Ram PK, Brooks WA, et al. Burden of typhoid and paratyphoid fever in a densely populated urban community, Dhaka, Bangladesh. Int J Infect Dis 2010; 14(Suppl. 3): e93-e9.	Initial systematic review	✓	✓	✓		✓	✓

Aljanaby AAJ, Medhat AR. Prevalence of some antimicrobials resistance associated-genes in *Salmonella* Typhi isolated from patients infected with typhoid fever. J Bio Sci 2017; 17(4): 171-84.	Initial systematic review	✓	✓			✓	
Al-Sanouri TM, Paglietti B, Haddadin A, et al. Typhoid fever in Jordan. Clin Microbiol Infect 2010; 16: S320.	Extended systematic review	✓	✓				
Al-Tawfiq JA. Antimicrobial susceptibility of *Salmonella* Typhi and non-Typhi in a hospital in eastern Saudi Arabia. J Chemother 2007; 19(1): 62-5.	Extended systematic review					✓	
Amatya NM, Shrestha B, Lekhak B. Etiological agents of bacteraemia and antibiotic susceptibility pattern in Kathmandu Model Hospital. JNMA J Nepal Med Assoc 2007; 46(167): 112-8.	Initial systematic review	✓	✓	✓	✓	✓	✓
Amdani SK. Antibiotic resistance pattern of pediatric typhoid fever patients at Harapan Kita Children and Maternity Hospital Jakarta, 1996. Med J Indones 1998; 7: 253-6.	Initial systematic review	✓		✓		✓	✓
Ammini P, Jasna V, Greeshma M, Preethi C. Comparative virulence genotyping and antimicrobial susceptibility profiling of environmental and clinical *Salmonella* enterica from Cochin, India. Current Microbiology 2011; 62(1): 21-6.	Extended systematic review	✓					
Anand AC. The anatomy of an epidemic (the final report on an epidemic of multidrug resistant enteric fever in eastern India). Trop Gastroenterol 1993; 14(1): 21-7.	Initial systematic review	✓					
Andrews JR, Qamar FN, Charles RC and Ryan ET. Extensively Drug-Resistant Typhoid - Are Conjugate Vaccines Arriving Just in Time?New England Journal of Medicine 2018;379(16):1493-1495	Initial systematic review	✓		✓		✓	✓
Anees A, Indu S, Fatima K, Anjum P. Multi-drug resistant *Salmonella* enterica subspecies enterica serotype Typhi: A diagnostic and therapeutic challenge. Int J Curr Microbiol Appl Sci 2015; 4(Special Issue 1): 19-25.	Initial systematic review	✓	✓			✓	
Anila B, Yashwant K, Anshu S, Mani KR. Re-emergence of chloramphenicol sensitive isolates of *Salmonella* enterica serovar Typhi isolates in India during 2013-14. Int J Curr Microbiol Appl Sci 2015; 4(6): 1081-6.	Extended systematic review	✓					

Anjum P, Qureshi AH, Rafi S. Fluroquinolone resistance in typhoidal *Salmonella* and its detection by nalidixic acid disc diffusion. J Pak Med Assoc 2004; 54(6): 295-301.	Initial systematic review				✓		
Ansari I, Adhikari N, Pandey R, Dangal MM, Karanjit R, Acharya A. Enteric fever: is ciprofloxacin failing in Nepal? Trop Doct 2005; 35(1): 57-8.	Extended systematic review	✓					
Anup P, Subrata G, Sanjucta D, Dipika S, Bhattacharya MK, Bhattacharya SK. Increasing prevalence of *Salmonella* enterica serotype Paratyphi-A in patients with enteric fever in a periurban slum setting of Kolkata, India. International Journal of Environmental Health Research 2006; 16(6): 455-9.	Extended systematic review				✓		✓
Araj GF, Uwaydah MM, Alami SY. Antimicrobial susceptibility patterns of bacterial isolates at the American University Medical Center in Lebanon. Diagn Microbiol Infect Dis 1994; 20(3): 151-8.	Extended systematic review	✓					
Arjyal A, Basnyat B, Koirala S, et al. Gatifloxacin versus chloramphenicol for uncomplicated enteric fever: an open-label, randomised, controlled trial. Lancet Infect Dis 2011; 11(6): 445-54.	Initial systematic review	✓	✓	✓	✓	✓	✓
Arjyal A, Basnyat B, Nhan HT, et al. Gatifloxacin versus ceftriaxone for uncomplicated enteric fever in Nepal: an open-label, two-centre, randomised controlled trial. Lancet Infect Dis 2016; 16(5): 535-45.	Initial systematic review		✓		✓	✓	✓
Arora D, Seetha KS, Kumar R. The changing scenario of the *Salmonella* serotype and its drug resistance pattern. J Clin Diagn Res 2009; 3(5): 1754-9.	Extended systematic review	✓		✓			
Arora D, Singh R, Kaur M, Ahi RS. A changing pattern in antimicrobial susceptibility of *Salmonella* enterica serotype isolated in North India. African Journal of Microbiology Research 2010; 4(3): 197-203.	Extended systematic review	✓		✓		✓	✓
Arora RK, Gupta A, Joshi NM, Kataria VK, Lall P, Anand AC. Multidrug resistant typhoid fever: Study of an outbreak in Calcutta. Indian Pediatr 1992; 29(1): 61-6.	Extended systematic review	✓					
ARSP Working Group. A multi centre laboratory study of Gram negative bacterial blood stream infections in Sri Lanka. Ceylon Med J 2013; 58(2): 56-61.	Initial systematic review	✓		✓		✓	✓

Arya SC, Agarwal N. Regarding "Typhoid fever in travelers: time for re-assessment". European Journal of Internal Medicine 2008; 19(2): 148-9.	Extended systematic review		✓			✓	
Aseffa A, Gedlu E, Asmelash T. Antibiotic resistance of prevalent *Salmonella* and shigella strains in northwest Ethiopia. East Afr Med J 1997; 74(11): 708-13.	Extended systematic review	✓					
Ashok R, Peter K, Joselyne N, Emma N. Antimicrobial susceptibility patterns of *Salmonella* Typhi from Kigali, Rwanda. Shiraz E Medical Journal 2010; 11(3): 117-21.	Extended systematic review	✓	✓			✓	
Asna SMZH, Ashraful Haq J. Decrease of antibiotic resistance in *Salmonella* Typhi isolated from patients attending hospitals of Dhaka City over a 3 year period. Int J Antimicrob Agents 2000; 16(3): 249-51.	Initial systematic review	✓				✓	
Awal BK, Koirala P, Jareju R, Dhungana GR, Wosti BP. Predictive value of NA resistance for increased minimum inhibitory concentration (MIC) of fluoroquinolone in clinical isolates of *Salmonella* enterica. Int J Antimicrob Agents 2015; 45: S80-S1.	Extended systematic review					✓	✓
Bahrmand AR, Velayati AA. Antimicrobial resistance pattern and plasmid profile of *Salmonella* Typhi isolated from an outbreak in Tehran province. Scandinavian Journal of Infectious Diseases 1997; 29(3): 265-9.	Extended systematic review	✓					
Bajracharya BL, Baral MR, Shakya S, Tuladhar P, Paudel M, Acharya B. Clinical profile and antibiotics response in typhoid fever. Kathmandu Univ Med J (KUMJ) 2006; 4(1): 25-9.	Initial systematic review					✓	
Bakthavatchalam YD, Kumar DT, Tayubi IA, et al. In vitro efficacy and in silico analysis of cefixime-ofloxacin combination for *Salmonella* Typhi from bloodstream infection. J Appl Microbiol 2017; 123(3): 615-24.	Extended systematic review	✓	✓			✓	
Baliga S, Shenoy S, Vidyalaxmi, Pereira P. Ciprofloxacin-resistant *Salmonella* Typhi. Natl Med J India 1999; 12(3): 138.	Initial systematic review	✓	✓				
Bandyopadhyay R, Balaji V, Yadav B, Jasmine S, Sathyendra S, Rupali P. Effectiveness of treatment regimens for Typhoid fever in the nalidixic acid-resistant S. Typhi (NARST) era in South India. Trop Doct 2018; 48(3): 182-8.	Extended systematic review	✓	✓	✓	✓	✓	✓

Bavdekar A, Chaudhari M, Bhave S, Pandit A. Ciprofloxacin in typhoid fever. Indian J Pediatr 1991; 58(3): 335-9.	Initial systematic review	✓					
Bayram Y, Guducuoglu H, Otlu B, et al. Epidemiological characteristics and molecular typing of *Salmonella* enterica serovar Typhi during a waterborne outbreak in Eastern Anatolia. Ann Trop Med Parasitol 2011; 105(5): 359-65.	Extended systematic review	✓	✓			✓	
Behl P, Gupta V, Sachdev A, Guglani V, Chander J. Patterns in antimicrobial susceptibility of *Salmonella*e isolated at a tertiary care hospital in northern India. Indian J Med Res 2017; 145(1): 124-8.	Extended systematic review	✓	✓	✓	✓	✓	✓
Beig FK, Ahmad F, Abqari S. Changing antibiotic sensitivity pattern and scope of chloramphenicol in the management of hospitalised patients of typhoid fever. J Indian Med Assoc 2009; 107(12): 862-5.	Initial systematic review	✓	✓			✓	
Bekur R, Vandana KE, Shivashankara KN, Valsalan R, Sathyanarayanan V. Paratyphoid fever-Emerging problem in South India. Asian Pac J Trop Med 2010; 3(10): 815-7.	Initial systematic review			✓			✓
Bello N, Kudu ATD, Adetokun AB, et al. Characterization and Antimicrobial Susceptibility Profile of Bacteraemia Causing Pathogens Isolated from Febrile Children with and without Sickle Cell Disease in Kano, Nigeria.Mediterranean Journal of Hematology & Infectious Diseases 2018;10(1):e2018016	Initial systematic review					✓	
Bhadoria DP, Agarwal MP, Dwivedi S. Multidrug resistant *Salmonella* Typhi in India. Indian J Pathol Microbiol 1993; 36(2): 190-1.	Extended systematic review	✓					
Bhaswati S, Manjira B, Niyogi SK. In vitro activity of cefpodoxime, an expanded-spectrum cephalosporin, against *Salmonella* enterica serotype Typhi. Antimicrob Agents Chemother 2008; 52(2): 802-3.	Extended systematic review					✓	
Bhaswati S, Shanta D, Dipika S, et al. Phage typing, biotyping & antimicrobial resistance profile of *Salmonella* enterica serotype Typhi from Kolkata. Indian J Med Res 2007; 125(5): 685-8.	Initial systematic review	✓	✓			✓	
Bhat KG, Andrade AT, Karadesai SG, Hemashettar BM, Patil CS. Antimicrobial susceptibility of *Salmonella* Typhi to quinolones & cephalosporins. Indian J Med Res 1998; 107: 247-51.	Initial systematic review		✓				

Bhat KG, Tripathy A, Rajagopal R, Ramachandran S. A simple broth-disk method to determine the minimum inhibitory concentration of ceftriaxone on *Salmonella* enterica serovar Typhi and Paratyphi. Indian J Pathol Microbiol 2009; 52(2): 189-90.	Initial systematic review					✓	
Bhatia JK, Mathur AD, Arora MM. Reemergence of chloramphenicol sensitivity in enteric fever. Med J Armed Forces India 2007; 63(3): 212-4.	Initial systematic review	✓	✓	✓	✓	✓	✓
Bhattacharya SK, Dutta D, Bhattacharya MK, et al. Multi-resistant typhoid fever. Natl Med J India 1992; 5(1): 41.	Initial systematic review	✓	✓				
Bhattacharya SS, Das U, Choudhury BK. Occurrence & antibiogram of *Salmonella* Typhi & S. Paratyphi A isolated from Rourkela, Orissa. Indian J Med Res 2011; 133: 431-3.	Initial systematic review	✓		✓		✓	✓
Bhattacharya SS, Das U. A steady decrease in occurrence of *Salmonella* Typhi infection in Rourkela, Orissa. Indian J Pathol Microbiol 2003; 46(3): 498-500.	Initial systematic review	✓				✓	
Bhetwal A, Maharjan A, Khanal PR and Parajuli NP. Enteric Fever Caused by *Salmonella* enterica Serovars with Reduced Susceptibility of Fluoroquinolones at a Community Based Teaching Hospital of Nepal.International Journal of Microbiology 2017;2017(2869458	Initial systematic review	✓	✓	✓	✓	✓	✓
Bhutta ZA. Impact of age and drug resistance on mortality in typhoid fever. Arch Dis Child 1996; 75(3): 214-7.	Extended systematic review	✓					
Bigogo G, Verani JR, Munywoki P, Audi A. KEMRI CDC Gram_project_pbids30July2020. Nairobi, Kenya; 2020	Dataset	✓	✓				
Blacksell SD, Sharma NP, Phumratanaprapin W, et al. Serological and blood culture investigations of Nepalese fever patients. Trans R Soc Trop Med Hyg 2007; 101(7): 686-90.	Initial systematic review		✓		✓		
Bouzenoune F, Debbih KK, Boudersa F, Kouhil S, Nezzar N. Antibiotic susceptibility of *Salmonella* enterica serovar Typhi isolated from blood cultures at the Ain M'lila hospital (Algeria), between 2005 and 2008. [French]. Med Mal Infect 2011; 41(4): 181-5.	Initial systematic review	✓	✓				
Breiman RF, Cosmas L, Njuguna H, et al. Population-based incidence of typhoid fever in an urban informal settlement and a rural area in Kenya: implications for typhoid vaccine use in Africa. PLoS ONE 2012; 7(1): e29119.	Initial systematic review	✓	✓			✓	

Britto CD, Dyson ZA, Duchene S, et al. Laboratory and molecular surveillance of paediatric typhoidal *Salmonella* in Nepal: Antimicrobial resistance and implications for vaccine policy.PLoS Neglected Tropical Diseases [electronic resource] 2018;12(4):e0006408	Initial systematic review	✓	✓	✓	✓	✓	
Brooks JT, Ochieng JB, Kumar L, et al. Surveillance for bacterial diarrhea and antimicrobial resistance in rural western Kenya, 1997-2003. Clin Infect Dis 2006; 43(4): 393-401.	Extended systematic review	✓	✓			✓	
Brooks WA, Hossain A, Goswami D, et al. Bacteremic typhoid fever in children in an urban slum, Bangladesh. Emerg Infect Dis 2005; 11(2): 326-9.	Initial systematic review	✓				✓	
Brown JC, Shanahan PM, Jesudason MV, Thomson CJ, Amyes SG. Mutations responsible for reduced susceptibility to 4-quinolones in clinical isolates of multi-resistant *Salmonella* Typhi in India. J Antimicrob Chemother 1996; 37(5): 891-900.	Extended systematic review		✓				
Buch NA, Hassan MU, Kakroo DK. Enteric fever--a changing sensitivity pattern, clinical profile and outcome. Indian Pediatr 1994; 31(8): 981-5.	Initial systematic review	✓					
Bushra R, Sial AA, Rizvi M, Shafiq Y, Aslam N, Bano N. Sensitivity pattern of ceftriaxone against different clinical isolates. Pak 2016; 29(1): 249-53.	Extended systematic review					✓	
Butt T, Ahmad RN, Salman M, Kazmi SY. Changing trends in drug resistance among typhoid *Salmonellae* in Rawalpindi, Pakistan. East Mediterr Health J 2005; 11(5-6): 1038-44.	Initial systematic review	✓		✓		✓	✓
Butt T, Khan MY, Ahmad RN, Salman M, Afzal RK. Validity of nalidixic acid screening in fluoroquinolone-resistant typhoid *Salmonellae*. J Coll Physicians Surg Pak 2006; 16(1): 31-4.	Initial systematic review		✓		✓		
Capoor MR, Deepthi N, Jitendra P, et al. Minimum inhibitory concentration of carbapenems and tigecycline against *Salmonella* spp. J Med Microbiol 2009; 58(3): 337-41.	Initial systematic review	✓	✓	✓	✓	✓	✓
Capoor MR, Nair D, Deb M, Aggarwal P. Enteric fever perspective in India: emergence of high-level ciprofloxacin resistance and rising MIC to cephalosporins. J Med Microbiol 2007; 56(Pt 8): 1131-2.	Extended systematic review	✓	✓	✓	✓	✓	✓

Capoor MR, Nair D, Hasan AS, Aggarwal P, Gupta B. Typhoid fever: narrowing therapeutic options in India. Southeast Asian J Trop Med Public Health 2006; 37(6): 1170-4.	Initial systematic review					✓	
Capoor MR, Nair D, Walia NS, et al. Molecular analysis of high-level ciprofloxacin resistance in *Salmonella* enterica serovar Typhi and S. Paratyphi A: need to expand the QRDR region. Epidemiol Infect 2009; 137(6): 871-8.	Extended systematic review		✓		✓		
Cesur S, Albayrak F, Birengel S, Sozen TH. Antibiotic susceptibilities of *Salmonella* and Shigella strains isolated from stool samples in the 1997-1999 period against ampicillin, cotrimoxazole, chloramphenicol and ciprofloxacin. [Turkish]. Infeksiyon Dergisi = Turkish Journal of Infection 2004; 18(1): 65-8.	Extended systematic review	✓					
Chand HJ, Rijal KR, Neupane B, Sharma VK, Jha B. Re-emergence of susceptibility to conventional first line drugs in *Salmonella* isolates from enteric fever patients in Nepal. J Infect Dev Ctries 2014; 8(11): 1483-7.	Initial systematic review	✓	✓	✓	✓		
Chandane P, Gandhi A, Bowalekar S. Study of antibiotic susceptibility pattern of *Salmonella* Typhi in children suffering from enteric fever. Ann Trop Med Public Health 2017; 10(2): 440-3.	Initial systematic review		✓			✓	
Chande C, Shrikhande S, Kapale S, Agrawal S, Fule RP. Change in antimicrobial resistance pattern of *Salmonella* Typhi in central India. Indian J Med Res 2002; 115: 248-50.	Initial systematic review	✓					
Chande CA, Chopdekar KA, Pradnya V, et al. Current trend of antibiotic sensitivity of *Salmonella* Typhi and other *Salmonella*e in Mumbai: A 5 years study. Indian J Med Microbiol 2016; 34(1): 115-6.	Initial systematic review	✓	✓				
Chandel DS, Chaudhry R, Dhawan B, Pandey A, Dey AB. Drug-resistant *Salmonella* enterica serotype Paratyphi A in India. Emerg Infect Dis 2000; 6(4): 420-1.	Extended systematic review			✓	✓		
Chandel DS, Nisar N, Thong KL, Pang T, Chaudhry R. Role of molecular typing in an outbreak of *Salmonella* Paratyphi A. Trop Gastroenterol 2000; 21(3): 121-3.	Extended systematic review			✓			✓
Chandra R, Srinivasan S, Nalini P, Rao RS. Multidrug resistant enteric fever. J Trop Med Hyg 1992; 95(4): 284-7.	Extended systematic review	✓					

Chatterjee S, Eshwara VK, Tellapragada C, Mukhopadhyay C. Azithromycin susceptibility among clinical isolates of *Salmonella*: Interfacing guidelines with routine practices. Indian J Med Microbiol 2016; 34(3): 397-8.	Extended systematic review	✓	✓	✓	✓	✓	✓
Chaudhary R, Sijapati K, Singh SK. A study on nalidixic acid resistant *Salmonella* among the patients attending Shree Birendra Hospital. Med J Shree Birendra Hosp 2011; 10(2): 1-3.	Initial systematic review		✓		✓		
Chen M, Wu J, Jiang J. Clinical and laboratory analysis of 79 cases of typhoid fever and paratyphoid fever. [Chinese]. Journal of Tropical Medicine 2006; 6(5): 570-2.	Extended systematic review	✓		✓			
Chheng K, Carter MJ, Emary K, et al. A prospective study of the causes of febrile illness requiring hospitalization in children in Cambodia. PLoS ONE 2013; 8(4): e60634.	Initial systematic review	✓	✓				
Chinh NT, Parry CM, Ly NT, et al. A randomized controlled comparison of azithromycin and ofloxacin for treatment of multidrug-resistant or nalidixic acid-resistant enteric fever. Antimicrob Agents Chemother 2000; 44(7): 1855-9.	Initial systematic review	✓					
Chinh NT, Solomon T, Thong MX, et al. Short courses of ofloxacin for the treatment of enteric fever. Trans R Soc Trop Med Hyg 1997; 91(3): 347-9.	Initial systematic review	✓					
Chiou CS, Lauderdale TL, Phung DC, et al. Antimicrobial resistance in *Salmonella* enterica serovar Typhi isolates from Bangladesh, Indonesia, Taiwan, and Vietnam. Antimicrob Agents Chemother 2014; 58(11): 6501-7.	Extended systematic review	✓	✓				
Chisti MJ, Bardhan PK, Huq S, et al. High-dose intravenous dexamethasone in the management of diarrheal patients with enteric fever and encephalopathy. Southeast Asian J Trop Med Public Health 2009; 40(5): 1065-73.	Extended systematic review	✓		✓			
Chitnis S, Chitnis V, Hemvani N, Chitnis DS. Ciprofloxacin therapy for typhoid fever needs reconsideration. J Infect Chemother 2006; 12(6): 402-4.	Extended systematic review	✓	✓				
Chopra GS, Basu SK, Bhattacharya SR. Present phage types and antibiotic susceptibility of *Salmonella*e. Indian J Pathol Microbiol 1992; 35(4): 345-50.	Extended systematic review	✓	✓	✓	✓		

Choudhary A, Gopalakrishnan R, Nambi PS, Ramasubramanian V, Ghafur KA, Thirunarayan MA. Antimicrobial susceptibility of *Salmonella* enterica serovars in a tertiary care hospital in southern India. Indian J Med Res 2013; 137(4): 800-2.	Extended systematic review	✓		✓	
Chowta MN, Chowta NK. Study of clinical profile and antibiotic response in typhoid fever. Indian J Med Microbiol 2005; 23(2): 125-7.	Initial systematic review	✓			✓
Chuang CH, Su LH, Perera J, et al. Surveillance of antimicrobial resistance of *Salmonella* enterica serotype Typhi in seven Asian countries. Epidemiol Infect 2009; 137(2): 266-9.	Extended systematic review	✓	✓		✓
Ciraj AM, Mohammed M, Bhat KG, Shivananda PG. Copper resistance and its correlation to multiple drug resistance in *Salmonella* Typhi isolates from south Karnataka. Indian J Med Res 2000; 110(DEC.): 181-2.	Initial systematic review	✓			✓
Ciraj AM, Seema DS, Bhat GK, Shivananda PG. Nalidixic acid screening test for the detection of decreased susceptibility to ciprofloxacin in *Salmonella* Typhi. Indian J Pathol Microbiol 2001; 44(4): 407-8.	Initial systematic review		✓		
Ciraj AM, Seetha KS, Gopalkrishna BK, Shivananda PG. Drug resistance pattern and phage types of *Salmonella* Typhi isolates in Manipal, South Karnataka. Indian J Med Sci 1999; 53(11): 486-9.	Initial systematic review	✓			✓
Clarke KR, Kanyanga MK, Musenga E, et al. Outbreak of multi-drug resistant *Salmonella* Typhi, lusaka, zambia 2011-2012. American Journal of Tropical Medicine and Hygiene 2012; 1: 318.	Extended systematic review		✓		
Crump JA, Ramadhani HO, Morrissey AB, et al. Invasive bacterial and fungal infections among hospitalized HIV-infected and HIV-uninfected adults and adolescents in northern Tanzania. Clin Infect Dis 2011; 52(3): 341-8.	Initial systematic review				✓
Crump JA, Ramadhani HO, Morrissey AB, et al. Invasive bacterial and fungal infections among hospitalized HIV-infected and HIV-uninfected children and infants in northern Tanzania. Trop Med Int Health 2011; 16(7): 830-7.	Extended systematic review				✓
Dagnra AY, Akolly K, Gbadoe A, Aho K, David M. Emergence of multidrug resistant *Salmonella* strains in Lome (Togo). [French]. Med Mal Infect 2007; 37(5): 266-9.	Extended systematic review	✓	✓		✓

Dahesihdewi A, Sugianli AK, Parwati I. Data surveilans mikroba dan kepekaannya terhadap antibiotik berdasarkan tipe rumah sakit di Indonesia tahun 2017. Jakarta Selatan, Indonesia, 2018.	Surveillance report					✓	✓
Dahesihdewi A, Sugianli AK, Parwati I. Surveilans mikroba dan kepejaannya terhadap antibiotik berdasarkan tipe rumah sakit di Indonesia tahun 2018. Jakarta Selatan, Indonesia, 2019.	Surveillance report	✓				✓	✓
Dahiya S, Sharma P, Kumari B, et al. Characterisation of antimicrobial resistance in *Salmonella*e during 2014-2015 from four centres across India: An ICMR antimicrobial resistance surveillance network report. Indian J Med Microbiol 2017; 35(1): 61-8.	Extended systematic review	✓	✓	✓	✓	✓	✓
Dar L, Gupta BL, Rattan A, Bhujwala RA, Shriniwas. Multidrug resistant *Salmonella* Typhi in Delhi. Indian J Pediatr 1992; 59(2): 221-4.	Initial systematic review	✓					
Das S, Ray U, Dutta S. Revisit of fluoroquinolone and azithromycin susceptibility breakpoints for *Salmonella* enterica serovar Typhi. J Med Microbiol 2016; 65(7): 632-40.	Extended systematic review		✓				
Das S, Samajpati S, Ray U, Roy I, Dutta S. Antimicrobial resistance and molecular subtypes of *Salmonella* enterica serovar Typhi isolates from Kolkata, India over a 15 years period 1998-2012. Int J Med Microbiol 2016; 25: 25.	Initial systematic review	✓	✓				
Das S, Samajpati S, Roy I, et al. Molecular Subtyping of *Salmonella* enterica Serovar Typhi by Pulsed-Field Gel Electrophoresis and Multiple-Locus Variable-Number Tandem-Repeat Analysis in India: Their Association with Antimicrobial Resistance Profiles. Jpn J Infect Dis 2017; 70(5): 536-43.	Initial systematic review	✓	✓			✓	
Das SK, Chisti MJ, Afrad MH, et al. Gastroenteritis due to typhoidal *Salmonella*: a decade of observation at an urban and a rural diarrheal disease hospital in Bangladesh. BMC Infect Dis 2014; 14: 435.	Extended systematic review	✓	✓	✓	✓	✓	✓
Das U, Bhattacharya SS. Antibiogram, phage typing and biotyping of *Salmonella* Typhi and *Salmonella* Paratyphi A from Rourkela, Orissa. Indian J Med Res 2006; 124(1): 109-11.	Initial systematic review	✓		✓		✓	✓
Das U, Bhattacharya SS. Multidrug resistant *Salmonella* Typhi in Rourkela, Orissa. Indian J Pathol Microbiol 2000; 43(2): 135-8.	Initial systematic review	✓				✓	

Dashti AA, Jadaon MM, Habeeb F, West PW, Panigrahi D, Amyes SG. *Salmonella* enterica Serotype Typhi in Kuwait and its reduced susceptibility to ciprofloxacin. J Chemother 2008; 20(3): 297-302.	Initial systematic review	✓				
Department of Health (Philippines) - Antimicrobial Resistance Suerveillance Program. Antimicrobial resistance surveillance program; 2014 data summary report. Alabang, Muntinlupa City, Philippines, 2014.	Surveillance report	✓	✓			
Department of Health (Philippines) - Antimicrobial Resistance Suerveillance Program. Antimicrobial resistance surveillance program; 2015 data summary report. Alabang, Muntinlupa City, Philippines, 2015.	Surveillance report	✓	✓			✓
Department of Health (Philippines) - Antimicrobial Resistance Suerveillance Program. Antimicrobial resistance surveillance program; 2016	Surveillance report	✓	✓			✓
Department of Health (Philippines) - Antimicrobial Resistance Suerveillance Program. Antimicrobial resistance surveillance program; 2017 data summary report. Alabang, Muntinlupa City, Philippines, 2017.	Surveillance report	✓	✓			
Dhanashree B. Antibiotic susceptibility profile of *Salmonella* enterica serovars: trend over three years showing re-emergence of chloramphenicol sensitivity and rare serovars. Indian J Med Sci 2007; 61(10): 576-9.	Initial systematic review	✓	✓	✓	✓	
Dhar KL, Thomas MS, Abraham G. Changing trends in the treatment of *Salmonella* Typhi infections in north-western India. J Assoc Physicians India 1993; 41(9): 616-7.	Initial systematic review	✓				
Dheer G, Kundra S, Goel A, Singh T, Berry V. Changing spectrum of antibiotic sensitivity in enteric fever- a six year retrospective study in north india. Res J Pharm Biol Chem Sci 2013; 4(4): 492-8.	Initial systematic review		✓			
Dilruba A, D'Costa LT, Khorshed A, Nair GB, Hossain MA. Multidrug-resistant *Salmonella* enterica serovar Typhi isolates with high-level resistance to ciprofloxacin in Dhaka, Bangladesh. Antimicrob Agents Chemother 2006; 50(10): 3516-7.	Initial systematic review	✓	✓			
Dimitrov T, Dashti AA, Albaksami O, Jadaon MM. Detection of mutations in the gyrA gene in fluoroquinolone resistance *Salmonella* enterica serotypes Typhi and Paratyphi A isolated from the Infectious Diseases Hospital, Kuwait. J Clin Pathol 2010; 63(1): 83-7.	Initial systematic review	✓	✓	✓	✓	
Dimitrov TS, Panigrahi D, Emara M, Al-Nakkas A, Awni F, Passadilla R. Incidence of bloodstream infections in a speciality hospital in Kuwait: 8-Year experience. Med Princ Pract 2005; 14(6): 417-21.	Initial systematic review	✓				✓

Dolecek C, Tran TP, Nguyen NR, et al. A multi-center randomised controlled trial of gatifloxacin versus azithromycin for the treatment of uncomplicated typhoid fever in children and adults in Vietnam. PLoS ONE, 2008. http://onlinelibrary.wiley.com/o/cochrane/clcentral/articles/763/CN-00639763/frame.html (accessed.	Extended systematic review	✓	✓	✓	✓	✓	✓
Dong BQ, Yang J, Wang XY, et al. Trends and disease burden of enteric fever in Guangxi province, China, 1994-2004. Bull World Health Organ 2010; 88(9): 689-96.	Extended systematic review	✓	✓	✓	✓		
Dongol S, Thompson CN, Clare S, et al. The microbiological and clinical characteristics of invasive *Salmonella* in gallbladders from cholecystectomy patients in kathmandu, Nepal. PLoS ONE 2012; 7(10): e47342.	Extended systematic review	✓	✓	✓	✓		
Doughari JH, Elmahmood AM, Nggada HP. Retrospective study on the antibiotic resistant pattern of *Salmonella* Typhi from some clinical samples. African Journal of Microbiology Research 2007; 1(3): 33-6.	Extended systematic review	✓					
Dougle M, Hendriks E, Sanders E, Dorigo-Zetsma JW. Laboratory investigations in the diagnosis of septicaemia and malaria. East Afr Med J 1997; 74(6): 353-6.	Initial systematic review	✓					
Dromigny JA, Perrier-Gros-Claude JD. Antimicrobial resistance of *Salmonella* enterica serotype Typhi in Dakar, Senegal. Clin Infect Dis 2003; 37(3): 465-6.	Extended systematic review	✓	✓				
Duggal S, Banerjee P, Chugh TD. Review of fifty culture proven *Salmonella* cases. Indian J Med Sci 2016; 68(1): 67-72.	Initial systematic review	✓	✓	✓	✓	✓	✓
Duggal S, Rongpharpi SR, Gur R, Nayar R, Arora VM. Etiology and susceptibility of blood stream infections in a Referral Hospital in north Delhi: A one year study. Res J Pharm Biol Chem Sci 2014; 5(2): 1859-64.	Initial systematic review					✓	
Dutta P, Mitra U, Datta S, et al. Ciprofloxacin susceptible *Salmonella* Typhi with treatment failure. J Trop Pediatr 2001b; 47(4): 252-3.	Initial systematic review	✓	✓			✓	
Dutta P, Mitra U, Dutta S, De A, Chatterjee MK, Bhattacharya SK. Ceftriaxone therapy in ciprofloxacin treatment failure typhoid fever in children. Indian J Med Res 2001a; 113: 210-3.	Initial systematic review					✓	

Dutta S, Das S, Mitra U, et al. Antimicrobial resistance, virulence profiles and molecular subtypes of *Salmonella* enterica serovars Typhi and Paratyphi A blood isolates from Kolkata, India during 2009-2013. PLoS ONE 2014; 9(8): e101347.	Initial systematic review	✓	✓	✓	✓	✓	✓
Dutta S, Sur D, Manna B, Bhattacharya SK, Deen JL, Clemens JD. Rollback of *Salmonella* enterica serotype Typhi resistance to chloramphenicol and other antimicrobials in Kolkata, India. Antimicrob Agents Chemother 2005; 49(4): 1662-3.	Initial systematic review	✓	✓			✓	
Dutta TK, Beeresha A, Ghotekar LH. Atypical manifestations of typhoid fever. J Postgrad Med 2001; 47(4): 248-51.	Extended systematic review	✓					
Easow JM, Joseph NM, Dhungel BA, Chapagain B, Shivananda PG. Blood stream infections among febrile patients attending a teaching hospital in Western Region of Nepal. Australasian Medical Journal 2010; 3(10): 633-7.	Extended systematic review			✓		✓	✓
Eibacha D, Al-Emrana HM, Dekker DM, et al. The emergence of reduced ciprofloxacin susceptibility in *Salmonella* enterica causing bloodstream infections in rural Ghana. Clin Infect Dis 2016; 62(Suppl. 1): S32-S6.	Initial systematic review	✓	✓				
Elumalai S, Muthu G, Selvam RE, Ramesh S. Detection of TEM-, SHV- and CTX-M-type beta-lactamase production among clinical isolates of *Salmonella* species. J Med Microbiol 2014; 63(Pt 7): 962-7.	Initial systematic review	✓	✓	✓	✓	✓	✓
Enabulele IO, Yah SC, Yusuf EO, Eghafona NO. Emerging quinolones resistant transfer genes among gram-negative bacteria, isolated from faeces of HIV/AIDS patients attending some Clinics and Hospitals in the City of Benin, Edo State, Nigeria. Online Journal of Health and Allied Sciences 2006; 5(3).	Extended systematic review		✓				
Erdem B, Ercis S, Hascelik G, et al. Antimicrobial resistance patterns and serotype distribution among *Salmonella* enterica strains in Turkey, 2000-2002. Eur J Clin Microbiol Infect Dis 2005; 24(3): 220-5.	Extended systematic review	✓	✓				
Farhana K, Sayeed MA, Feroza Kaneez C, et al. Typhoid fever in young children in Bangladesh: clinical findings, antibiotic susceptibility pattern and immune responses. PLoS Negl Trop Dis 2015; 9(4).	Initial systematic review	✓	✓			✓	
Farheen Z, Shahnaz U, Sakina F, Muhammad M-u-H. Comparative study of antibiotics for their antimicrobial susceptibility in clinical isolates. Int J Pharm Sci Res 2015; 6(2): 630-5.	Extended systematic review					✓	

Fashae K, Ogunsola F, Aarestrup FM, Hendriksen RS. Antimicrobial susceptibility and serovars of *Salmonella* from chickens and humans in Ibadan, Nigeria. J Infect Dev Ctries 2010; 4(8): 484-94.	Extended systematic review	✓	✓				
Francisco M, Costa SS, Belas A, et al. First report on antimicrobial resistance and molecular characterisation of *Salmonella* enterica serotype Typhi isolated from human specimens in Luanda, Angola. Journal of Global Antimicrobial Resistance 2018; 13: 246-9.	Extended systematic review	✓	✓				
Frenck Jr RW, Mansour A, Nakhla I, et al. Short-course azithromycin for the treatment of uncomplicated typhoid fever in children and adolescents. Clin Infect Dis 2004; 38(7): 951-7.	Extended systematic review	✓				✓	
Frenck R, Nakhla I, Sultan Y, et al. Azithromycin versus ceftriaxone for the treatment of uncomplicated typhoid fever in children. Clinical infectious diseases : an official publication of the Infectious Diseases Society of America, 2000. http://onlinelibrary.wiley.com/o/cochrane/clcentral/articles/607/CN-00328607/frame.html	Extended systematic review	✓				✓	
Gandhi R, Banker DD. Multidrug resistant *Salmonella*. Indian J Med Sci 1999; 53(6): 259-66.	Extended systematic review	✓		✓			
Ganesh R, Janakiraman L, Vasanthi T, Sathiyasekeran M. Profile of typhoid fever in children from a tertiary care hospital in Chennai-South India. Indian J Pediatr 2010; 77(10): 1089-92.	Initial systematic review		✓				
Garg A, Anupurba S, Garg J, Goyal RK, Sen MR. Bacteriological profile and antimicrobial resistance of blood culture isolates from a university hospital. J Indian Acad Clin Med 2007; 8(2): 139-43.	Initial systematic review	✓				✓	
Garg K, Mangal N, Mathur HC. Clinical profile of multi drug resistant typhoid fever in Jaipur City. Indian Pediatr 1994; 31(2): 191-3.	Initial systematic review	✓					
Gasem MH, Keuter M, Dolmans WM, Van Der Ven-Jongekrijg J, Djokomoeljanto R, Van Der Meer JW. Persistence of *Salmonella*e in blood and bone marrow: randomized controlled trial comparing ciprofloxacin and chloramphenicol treatments against enteric fever. Antimicrob Agents Chemother 2003; 47(5): 1727-31.	Extended systematic review	✓		✓			
Gautam V, Gupta NK, Chaudhary U, Arora DR. Sensitivity pattern of *Salmonella* serotypes in Northern India. Braz J Infect Dis 2002; 6(6): 281-7.	Initial systematic review	✓	✓	✓		✓	✓

Geetha VK, Yugendran T, Srinivasan R, Harish BN. Plasmid-mediated quinolone resistance in typhoidal *Salmonella*e: a preliminary report from South India. Indian J Med Microbiol 2014; 32(1): 31-4.	Initial systematic review	✓	✓	✓	✓	✓	✓
Ghatole M, Manthalkar P, Kandle S, Yemul V, Jahagirdar V. Correlation of extended spectrum beta lactamases production with cephalosporin resistance in gram negative bacilli. Indian J Pathol Microbiol 2004; 47(1): 82-4.	Extended systematic review					✓	
Girgis NI, Butler T, Frenck RW, et al. Azithromycin versus ciprofloxacin for treatment of uncomplicated typhoid fever in a randomized trial in Egypt that included patients with multidrug resistance. Antimicrob Agents Chemother 1999; 43(6): 1441-4.	Extended systematic review	✓					
Girgis NI, Kilpatrick ME, Farid Z, Sultan Y, Podgore JK. Cefixime in the treatment of enteric fever in children. Drugs Exp Clin Res 1993; 19(1): 47-9.	Extended systematic review	✓				✓	
Girgis NI, Sultan Y, Hammad O, Farid Z. Comparison of the efficacy, safety and cost of cefixime, ceftriaxone and aztreonam in the treatment of multidrug-resistant *Salmonella* Typhi septicemia in children. Pediatr Infect Dis J 1995; 14(7): 603-5.	Initial systematic review	✓				✓	
Gopal M, Arumugam S, Gnadesikan S, Ramesh S. Studies on antimicrobial susceptibility pattern of *Salmonella* isolates from Chennai, India. Int J Pharma Bio Sci 2011; 2(2).	Extended systematic review	✓	✓	✓	✓	✓	✓
Gordon MA, Walsh AL, Chaponda M, et al. Bacteraemia and mortality among adult medical admissions in Malawi - Predominance of non-Typhi *Salmonella*e and Streptococcus pneumoniae. J Infect 2001; 42(1): 44-9.	Initial systematic review	✓					
Gross U, Amuzu SK, Ciman Rd, et al. Bacteremia and antimicrobial drug resistance over Time, Ghana. Emerg Infect Dis 2011; 17(10): 1879-82.	Initial systematic review	✓					
Gu W, Yang Z, Chen Y, et al. Molecular characteristics of *Salmonella* enterica Paratyphi A in Yunnan Province, southwest China. Infect Genet Evol 2015; 30: 181-5.	Initial systematic review				✓		
Guha S, Jalan BY, Dey S, Easow JM, Wilson G, Shivananda PG. *Salmonella* bacteraemia in Pokhara: emergence of antibiotic resistance. Nepal Med Coll J 2005; 7(1): 23-5.	Initial systematic review	✓		✓		✓	✓

Gundogdu A, Kilic H, Ulu-Kilic A, Parkan OM, Ture Z. Distribution and Antimicrobial Resistance of *Salmonella* Serovars Isolated in Kayseri Region. Klimik Journal 2017; 30(1): 22-6.	Extended systematic review	✓	✓				
Gupta S, Handa A, Chadha DS, Ganjoo RK, Panda RC. Profile of culture positive enteric fever from Bangalore. Med J Armed Forces India 2009; 65(4): 328-31.	Extended systematic review	✓	✓	✓	✓	✓	✓
Gupta S, Meena HS. Changing profile of enteric fever--in summer-91. J Assoc Physicians India 1992; 40(11): 726-9.	Initial systematic review	✓					
Gupta V, Kaur J, Chander J. An increase in enteric fever cases due to *Salmonella* Paratyphi A in & around Chandigarh. Indian J Med Res 2009a; 129(1): 95-8.	Initial systematic review			✓	✓		
Gupta V, Kaur J, Kaistha N. Re-emerging chloramphenicol sensitivity and emerging low level ciprofloxacin resistance among *Salmonella* enterica serotype Typhi isolates in North India. Trop Doct 2009; 39(1): 28-30.	Initial systematic review	✓	✓				
Gupta V, Singla N, Bansal N, Kaistha N, Chander J. Trends in the antibiotic resistance patterns of enteric fever isolates - a three year report from a tertiary care centre. Malays J Med Sci 2013; 20(4): 71-5.	Initial systematic review	✓	✓	✓	✓	✓	✓
Gurung B, Pandey S, Shah DK, et al. Antibiogram pattern of *Salmonella* in blood samples of enteric fever patients at lalitpur, nepal. Asian Pac J Trop Dis 2017; 7(1): 21-4.	Initial systematic review	✓	✓	✓	✓	✓	✓
Hafeez S, Izhar M, Ahmed A, Zafar A, Naeem M. In-vitro antimicrobial activity of Cefpirome: a new fourth-generation cephalosporin against clinically significant bacteria. J Pak Med Assoc 2000; 50(8): 250-2.	Extended systematic review					✓	
Hafiz S, Habib F, Ahmad N, Haq I, Husain R. Typhoid fevers: treatment with lomefloxacin. J Pak Med Assoc 1998; 48(6): 168-70.	Initial systematic review	✓		✓		✓	✓
Haldar KK, Basak S, Chakraborty AK, Das S. Transferable drug resistance in *Salmonella* Typhi strains isolated from an outbreak at Calcutta in the recent past. J Indian Med Assoc 1995; 93(8): 299-300, 15.	Initial systematic review	✓					

Halder KK, Saha Dalal B, Ghose E, Sanyal S. Chloramphenicol resistant *Salmonella* Typhi: The cause of recent outbreak of enteric fever in Calcutta. Indian J Pathol Microbiol 1992; 35(1): 11-7.	Initial systematic review	✓					
Hamze M, Dabboussi F, Izard D. Enterobacterial susceptibility to antibiotics in northern Lebanon (1998-2001). Cahiers Sante 2003; 13(2): 107-12.	Extended systematic review	✓					
Hannan A. Changing pattern of *Salmonella* Typhi isolates in Pakistan and their unchanged response to ofloxacin. International Journal of Experimental and Clinical Chemotherapy 1991; 4(4): 225-9.	Initial systematic review	✓					
Hardjo Lugito NP, Cucunawangsih. Antimicrobial resistance of *Salmonella* enterica serovars Typhi and Paratyphi isolates from a general hospital in Karawaci, Tangerang, Indonesia: A five-year review. Int J Microbiol 2017; 2017: 6215136.	Initial systematic review		✓		✓	✓	✓
Harichandran D, Dinesh KR. Antimicrobial susceptibility profile, treatment outcome and serotype distribution of clinical isolates of *Salmonella* enterica subspecies enterica: a 2-year study from Kerala, South India. Infect Drug Resist 2017; 10: 97-101.	Initial systematic review	✓	✓	✓	✓	✓	✓
Harish BN, Menezes GA, Sarangapani K, Parija SC. Fluoroquinolone resistance among *Salmonella* enterica serovar Paratyphi A in Pondicherry [2]. Indian J Med Res 2006; 124: 585-7.	Initial systematic review		✓		✓	✓	✓
Harish R, Sharma DB. Cefotaxime in multi drug resistant typhoid fever. Indian Pediatr 1994; 31(2): 193-6.	Extended systematic review	✓					
Harrois D, Breurec S, Seck A, et al. Prevalence and characterization of extended-spectrum beta-lactamase-producing clinical *Salmonella* enterica isolates in Dakar, Senegal, from 1999 to 2009. Clin Microbiol Infect 2014; 20(2): O109-16.	Extended systematic review	✓	✓	✓	✓	✓	
Hasan R, Zafar A, Abbas Z, Mahraj V, Malik F, Zaidi A. Antibiotic resistance among *Salmonella* enterica serovars Typhi and Paratyphi A in Pakistan (2001-2006). J Infect Dev Ctries 2008; 2(4): 289-94.	Initial systematic review	✓		✓			
Hazir T, Qazi SA, Abbas KA, Khan MA. Therapeutic re-appraisal of multiple drug resistant *Salmonella* Typhi (MDRST) in Pakistani children. J Pak Med Assoc 2002; 52(3): 123-7.	Initial systematic review	✓					

Hendriksen RS, Leekitcharoenphon P, Lukjancenko O, et al. Genomic signature of multidrug-resistant *Salmonella* enterica serovar Typhi isolates related to a massive outbreak in Zambia between 2010 and 2012. J Clin Microbiol 2015; 53(1): 262-72.	Extended systematic review	✓	✓				
Hermans PW, Saha SK, van Leeuwen WJ, Verbrugh HA, van Belkum A, Goessens WH. Molecular typing of *Salmonella* Typhi strains from Dhaka (Bangladesh) and development of DNA probes identifying plasmid-encoded multidrug-resistant isolates. J Clin Microbiol 1996; 34(6): 1373-9.	Initial systematic review	✓					
Hien TT, Bethell DB, Hoa NTT, et al. Short course of ofloxacin for treatment of multidrug-resistant typhoid. Clin Infect Dis 1995; 20(4): 917-23.	Initial systematic review	✓		✓		✓	✓
Hien TT, Duong NM, Ha HD, et al. A randomized comparative study of fleroxacin and ceftriaxone in enteric fever. Trans R Soc Trop Med Hyg 1994; 88(4): 464-5.	Initial systematic review	✓				✓	
Hoa NTT, Diep TS, Wain J, et al. Community-acquired septicaemia in southern Viet Nam: The importance of multidrug-resistant *Salmonella* Typhi. Trans R Soc Trop Med Hyg 1998; 92(5): 503-8.	Initial systematic review	✓		✓		✓	✓
Holt KE, Baker S, Dongol S, et al. High-throughput bacterial SNP typing identifies distinct clusters of *Salmonella* Typhi causing typhoid in Nepalese children. BMC Infect Dis 2009; 10(144).	Initial systematic review	✓	✓			✓	
Holt KE, Dutta S, Manna B, et al. High-resolution genotyping of the endemic *Salmonella* Typhi population during a Vi (typhoid) vaccination trial in Kolkata. PLoS Negl Trop Dis 2012; 6(1): e1490.	Initial systematic review	✓	✓				
Hong Le TA, Lejay-Collin M, Grimont PAD, et al. Endemic, epidemic clone of *Salmonella* enterica Serovar Typhi harboring a single multidrug-resistant plasmid in Vietnam between 1995 and 2002. J Clin Microbiol 2004; 42(7): 3094-9.	Extended systematic review	✓					
Hoque SS, Alam AN, Islam MR, Khan MR. Recent advances in the treatment of typhoid: With special emphasis on multidrug resistant *Salmonella* Typhi in Bangladesh. Bangladesh J Child Health 1992; 16(1-2): 15-9.	Extended systematic review	✓					
Hosoglu S, Loeb M, Geyik MF, Ucmak H, Jayaratne P. Molecular epidemiology of invasive *Salmonella* Typhi in southeast Turkey. Clin Microbiol Infect 2003; 9(7): 727-30.	Initial systematic review	✓					

Huenger F, Agyekum A, Nkrumah B, et al. Massive emergence of multidrug-resistant Enterobacteriaceae in blood culture isolates of children in Ghana. Clin Microbiol Infect 2010; 16: S88.	Initial systematic review	✓				✓	
Ikram S, Hussain S, Aslam A, Khan MD, Ahmed I. Evaluation of the current trends in the antimicrobial susceptibility patterns of typhoid *Salmonella*e. Pak J Med Health Sci 2015; 9(4): 1247-50.	Initial systematic review	✓	✓	✓	✓	✓	✓
Imran M, Dost S and Saleem M. Pattern of antibiotic resistance among patients with enteric fever.Indo American Journal of Pharmaceutical Sciences 2018;5(8):7797-7801	Initial systematic review	✓					
International Centre for Diarrhoeal Disease Research Bangladesh. Surveillance updates. Health and Science Bulletin 2006; 4(3).	Surveillance report	✓				✓	
International Centre for Diarrhoeal Disease Research Bangladesh. Surveillance updates. Health and Science Bulletin 2006; 4(4).	Surveillance report	✓				✓	
International Centre for Diarrhoeal Disease Research Bangladesh. Surveillance updates. Health and Science Bulletin 2007; 5(1).	Surveillance report	✓				✓	
International Centre for Diarrhoeal Disease Research Bangladesh. Surveillance updates. Health and Science Bulletin 2007; 5(2).	Surveillance report	✓				✓	
International Centre for Diarrhoeal Disease Research Bangladesh. Surveillance updates. Health and Science Bulletin 2007; 5(3).	Surveillance report	✓				✓	
International Centre for Diarrhoeal Disease Research Bangladesh. Surveillance updates. Health and Science Bulletin 2007; 5(4).	Surveillance report	✓				✓	
International Centre for Diarrhoeal Disease Research Bangladesh. Surveillance updates. Health and Science Bulletin 2008; 6(1).	Surveillance report	✓	✓			✓	
International Centre for Diarrhoeal Disease Research Bangladesh. Surveillance updates. Health and Science Bulletin 2008; 6(2).	Surveillance report	✓	✓			✓	
International Centre for Diarrhoeal Disease Research Bangladesh. Surveillance updates. Health and Science Bulletin 2008; 6(3).	Surveillance report	✓	✓			✓	
International Centre for Diarrhoeal Disease Research Bangladesh. Surveillance updates. Health and Science Bulletin 2008; 6(4).	Surveillance report	✓	✓			✓	
International Centre for Diarrhoeal Disease Research Bangladesh. Surveillance updates. Health and Science Bulletin 2009; 7(1).	Surveillance report	✓	✓			✓	

International Centre for Diarrhoeal Disease Research Bangladesh. Surveillance updates. Health and Science Bulletin 2009; 7(2).	Surveillance report	✓	✓			✓	
International Centre for Diarrhoeal Disease Research Bangladesh. Surveillance updates. Health and Science Bulletin 2009; 7(3).	Surveillance report	✓	✓			✓	
International Centre for Diarrhoeal Disease Research Bangladesh. Surveillance updates. Health and Science Bulletin 2009; 7(4).	Surveillance report	✓	✓			✓	
International Centre for Diarrhoeal Disease Research Bangladesh. Surveillance updates. Health and Science Bulletin 2010; 8(2).	Surveillance report	✓	✓			✓	
International Centre for Diarrhoeal Disease Research Bangladesh. Surveillance updates. Health and Science Bulletin 2010; 8(3).	Surveillance report	✓	✓			✓	
International Centre for Diarrhoeal Disease Research Bangladesh. Surveillance updates. Health and Science Bulletin 2010; 8(4).	Surveillance report	✓	✓			✓	
International Centre for Diarrhoeal Disease Research Bangladesh. Surveillance updates. Health and Science Bulletin 2011; 2(1).	Surveillance report	✓	✓			✓	
International Centre for Diarrhoeal Disease Research Bangladesh. Surveillance updates. Health and Science Bulletin 2011; 9(1).	Surveillance report	✓	✓			✓	
International Centre for Diarrhoeal Disease Research Bangladesh. Surveillance updates. Health and Science Bulletin 2011; 9(3): 22-5.	Surveillance report	✓	✓			✓	
International Centre for Diarrhoeal Disease Research Bangladesh. Surveillance updates. Health and Science Bulletin 2011; 9(4).	Surveillance report	✓	✓			✓	
International Centre for Diarrhoeal Disease Research Bangladesh. Surveillance updates. Health and Science Bulletin 2012; 10(1).	Surveillance report	✓	✓			✓	
International Centre for Diarrhoeal Disease Research Bangladesh. Surveillance updates. Health and Science Bulletin 2012; 10(2).	Surveillance report	✓	✓			✓	
International Centre for Diarrhoeal Disease Research Bangladesh. Surveillance updates. Health and Science Bulletin 2012; 10(3).	Surveillance report	✓	✓			✓	
International Centre for Diarrhoeal Disease Research Bangladesh. Surveillance updates. Health and Science Bulletin 2012; 10(4).	Surveillance report	✓	✓			✓	
International Centre for Diarrhoeal Disease Research Bangladesh. Surveillance updates. Health and Science Bulletin 2013; 11(1).	Surveillance report	✓	✓			✓	

International Centre for Diarrhoeal Disease Research Bangladesh. Surveillance updates. Health and Science Bulletin 2013; 11(2).	Surveillance report	✓	✓			✓	
International Centre for Diarrhoeal Disease Research Bangladesh. Surveillance updates. Health and Science Bulletin 2013; 11(3).	Surveillance report	✓	✓			✓	
International Centre for Diarrhoeal Disease Research Bangladesh. Surveillance updates. Health and Science Bulletin 2013; 11(4).	Surveillance report	✓	✓			✓	
International Centre for Diarrhoeal Disease Research Bangladesh. Surveillance updates. Health and Science Bulletin 2014; 12(1).	Surveillance report	✓	✓			✓	
International Centre for Diarrhoeal Disease Research Bangladesh. Surveillance updates. Health and Science Bulletin 2014; 12(2).	Surveillance report	✓	✓			✓	
International Centre for Diarrhoeal Disease Research Bangladesh. Surveillance updates. Health and Science Bulletin 2014; 12(3).	Surveillance report	✓	✓			✓	
International Centre for Diarrhoeal Disease Research Bangladesh. Surveillance updates. Health and Science Bulletin 2014; 12(4).	Surveillance report	✓	✓			✓	
International Centre for Diarrhoeal Disease Research Bangladesh. Surveillance updates. Health and Science Bulletin 2016; 14(1).	Surveillance report	✓	✓			✓	
Isbandrio BB, Gasem MH, Dolmans WMV, Hoogkamp-Korstanje JAA. Comparative activities of three quinolones and seven comparison standard drugs against *Salmonella* Typhi from Indonesia [1]. J Antimicrob Chemother 1994; 33(5): 1055-6.	Extended systematic review	✓	✓				
Islam MN, Afroza A, Hasan Z, Majumder B, Hossain A. Recent antibiogram pattern and clinical profile of typhoid fever in children - A study of 36 cases. Bangladesh J Child Health 1993; 17(3): 93-6.	Initial systematic review		✓		✓	✓	✓
Iyer RN, Jangam RR, Jacinth A, Venkatalakshmi A, Nahdi FB. Prevalence and trends in the antimicrobial susceptibility pattern of *Salmonella* enterica serovars Typhi and Paratyphi A among children in a pediatric tertiary care hospital in South India over a period of ten years: a retrospective study. Eur J Clin Microbiol Infect Dis 2017; 07: 07.	Initial systematic review	✓	✓	✓	✓	✓	✓
Jain S, Chugh TD. Antimicrobial resistance among blood culture isolates of *Salmonella* enterica in New Delhi. J Infect Dev Ctries 2013; 7(11): 788-95.	Initial systematic review	✓	✓	✓	✓	✓	✓

Javaid H, Zafar A, Ahmed JM, Ejaz H, Zubair M. Changing patterns of antimicrobial susceptibility of *Salmonella* Typhi at the children's hospital Lahore. Pak J Med Health Sci 2012; 6(1): 201-4.	Initial systematic review		✓			✓	
Jesudasan MV, John TJ. Multiresistant *Salmonella* Typhi in India. Lancet 1990; 336(8709): 252.	Extended systematic review	✓					
Jesudason M, Malathy B, John T. Trend of increasing levels of minimum inhibitory concentration of ciprofloxacin to *Salmonella* Typhi. The Indian journal of medical research 1996; 103: 247-9.	Extended systematic review		✓				
Jesudason MV, John TJ. Plasmid mediated multidrug resistance in *Salmonella* Typhi. Indian J Med Res 1992; 95: 66-7.	Initial systematic review	✓					
Jiang Y, Zuo H, Niu H, et al. Antimicrobial resistance surveillance of *Salmonella* isolates from the First People's Hospital of Yunnan Province, China. J Infect Dev Ctries 2015; 9(4): 333-7.	Extended systematic review	✓	✓	✓	✓	✓	✓
Jog S, Soman R, Singhal T, Rodrigues C, Mehta A, Dastur FD. Enteric fever in Mumbai--clinical profile, sensitivity patterns and response to antimicrobials. J Assoc Physicians India 2008; 56: 237-40.	Initial systematic review	✓	✓	✓	✓		✓
Joshi BG, Keyal K, Pandey R, Shrestha BM. Clinical profile and sensitivity pattern of *Salmonella* serotypes in children: A hospital based study. Journal of Nepal Paediatric Society 2011; 31(3): 180-3.	Initial systematic review	✓	✓	✓	✓	✓	✓
Joshi S, Adhikary R, Beena HB, et al. Trends in antibiotic susceptibility of enteric fever isolates from South India, 2002–2013.Medical Journal Armed Forces India 2019;75(1):81-85	Initial systematic review	✓	✓	✓	✓	✓	✓
Joshi S, Amarnath SK. Fluoroquinolone resistance in *Salmonella* Typhi and S. Paratyphi A in Bangalore, India. Trans R Soc Trop Med Hyg 2007; 101(3): 308-10.	Initial systematic review	✓	✓		✓	✓	✓
Joshi S, Wattal C, Sharma A, Oberoi JK, Prasad KJ. Quinolones - Drug of choice for enteric fever? Indian J Med Microbiol 2004; 22(4): 271-2.	Initial systematic review		✓		✓		

Joshi S. Antibiogram of S. enterica serovar Typhi and S. enterica serovar Paratyphi A: a multi-centre study from India. WHO South-East Asia j 2012; 1(2): 182-8.	Extended systematic review	✓	✓	✓	✓	✓	✓
Kabra SK, Madhulika, Talati A, Soni N, Patel S, Modi RR. Multidrug-resistant typhoid fever. Trop Doct 2000; 30(4): 195-7.	Initial systematic review	✓				✓	
Kadappu KK, Bhat R, Kurian B. Pattern of multiple drug resistance in enteric fever in Manipal, India. Trop Doct 2003; 33(3): 189-91.	Initial systematic review	✓		✓		✓	✓
Kalonji LM, Post A, Phoba MF, et al. Invasive *Salmonella* infections at multiple aurveillance aites in the Democratic Republic of the Congo, 2011-2014. Clin Infect Dis 2015; 61: S346-S53.	Initial systematic review	✓	✓			✓	
Kam KM, Luey KY, Chiu AW, Law CP, Leung SF. Molecular characterization of *Salmonella* enterica Serotype Typhi isolates by pulsed-field gel electrophoresis in Hong Kong, 2000-2004. Foodborne Pathog Dis 2007; 4(1): 41-9.	Extended systematic review	✓					
Kanj SS, Kanafani ZA, Shehab M, et al. Epidemiology, clinical manifestations, and molecular typing of *Salmonella* Typhi isolated from patients with typhoid fever in Lebanon. J Epidemiol Glob Health 2015; 5(2): 159-65.	Extended systematic review		✓			✓	
Kapil A, Renuka, Das B. Nalidixic acid susceptibility test to screen ciprofloxacin resistance in *Salmonella* Typhi. Indian J Med Res 2002; 115(February): 49-54.	Initial systematic review	✓	✓				
Kapil A, Sharma P, Dahiya S, Sood S, Das B. Trends of Increasing Antimicrobial Resistance in Community Acquired infections- a Case Study of Enteric Fever and Health Implications. International Journal of Epidemiology 2015; 44: 99-100.	Extended systematic review		✓		✓		
Kapil A, Sood S, Dash NR, Das BK, Seth P. Ciprofloxacin in typhoid fever.[Erratum appears in Lancet 1999 Sep 4;354(9181):870]. Lancet 1999; 354(9173): 164.	Extended systematic review		✓				
Kapil A, Sood S, Reddaiah VP, Das B, Seth P. Paratyphoid fever due to *Salmonella* enterica serotype Paratyphi A. Emerg Infect Dis 1997; 3(3): 407.	Initial systematic review			✓			✓

Karamat K, Butt T, Hannan A, et al. Problem of multi-drug resistant typhoid fever in Rawalpindi/Islamabad area. Pak Armed Forces Med J 1996; 46(2): 48-54.	Initial systematic review	✓		✓			
Kariuki S, Gilks C, Revathi G, Hart CA. Genotypic analysis of multidrug-resistant *Salmonella* enterica Serovar Typhi, Kenya. Emerg Infect Dis 2000; 6(6): 649-51.	Initial systematic review	✓	✓				
Kariuki S, Revathi G, Kiiru J, et al. Typhoid in Kenya is associated with a dominant multidrug-resistant *Salmonella* enterica serovar Typhi haplotype that is also widespread in Southeast Asia. J Clin Microbiol 2010; 48(6): 2171-6.	Initial systematic review	✓	✓				
Kariuki S, Revathi G, Kingsley R, Holt K, Dougan G. Emerging quinolone resistance in typhoid outbreaks caused by haplotype H58 in Kenya. Clin Microbiol Infect 2011; 17: S109.	Extended systematic review	✓					
Kariuki S, Revathi G, Muyodi J, et al. Characterization of multidrug-resistant typhoid outbreaks in Kenya. J Clin Microbiol 2004; 42(4): 1477-82.	Initial systematic review	✓	✓				
Karki AB, Bhatta DR, Shrestha B, et al. Higher nalidixic acid resistance pattern of *Salmonella* isolates from enteric fever patients in kathmandu model hospital Nepal. Res J Pharm Biol Chem Sci 2013; 4(2): 1687-93.	Initial systematic review	✓	✓	✓	✓	✓	✓
Karmaker S, Biswas D, Shaikh NM, Chatterjee SK, Kataria VK, Kumar R. Role of a large plasmid of *Salmonella* Typhi encoding multiple drug resistance. J Med Microbiol 1991; 34(3): 149-51.	Extended systematic review	✓	✓				
Karunaratne GK, Wickremesinghe RS, Perera KC. *Salmonella* Typhi and chloramphenicol resistance. Ceylon Med J 2000; 45(3): 136-7.	Extended systematic review	✓				✓	
Kasper MR, Sokhal B, Blair PJ, Wierzba TF, Putnam SD. Emergence of multidrug-resistant *Salmonella* enterica serovar Typhi with reduced susceptibility to fluoroquinolones in Cambodia. Diagn Microbiol Infect Dis 2010; 66(2): 207-9.	Initial systematic review	✓	✓			✓	
Kato Y, Fukayama M, Adachi T, et al. Multidrug-resistant typhoid fever outbreak in travelers returning from Bangladesh. Emerg Infect Dis 2007; 13(12): 1954-5.	Extended systematic review	✓	✓				

Kaur A, Singh VA. Bacterial isolates and their antibiotic sensitivity pattern in clinically suspected cases of fever of unknown origin. JK Science 2014; 16(3): 105-9.	Extended systematic review	✓				✓	
Kavai SM, Kangogo M, Muigai AWT, Kariuki S. Analysis of Trends in Resistance to Fluoroquinolones and Extended Spectrum Beta-Lactams among *Salmonella* Typhi Isolates Obtained from Patients at Four Outpatient Clinics in Nairobi County, Kenya. Advances in Microbiology 2018; 8(7): 578-88.	Extended systematic review	✓	✓			✓	
Kavita N, Channappa ST, Gaddad SM. Antimicrobial susceptibility of *Salmonella* Typhi in India. J Infect Dev Ctries 2010; 4(2): 70-3.	Initial systematic review	✓	✓			✓	
Kelly DF, Thorson S, Maskey M, et al. The burden of vaccine-preventable invasive bacterial infections and pneumonia in children admitted to hospital in urban Nepal. Int J Infect Dis 2011; 15(1): e17-23.	Extended systematic review			✓			
Khan FY, Elshafie SS, Almaslamani M, et al. Epidemiology of bacteraemia in Hamad general hospital, Qatar: A one year hospital-based study. Travel Med Infect Dis 2010; 8(6): 377-87.	Initial systematic review					✓	
Khan JA, Shukla I, Malik A. Plasmid mediated multidrug resistance in *Salmonella* Typhi. Biomedicine 1999; 19(2): 107-11.	Extended systematic review	✓	✓				
Khan MI, Soofi SB, Ochiai Leon R, et al. Epidemiology, clinical presentation, and patterns of drug resistance of *Salmonella* Typhi in Karachi, Pakistan. J Infect Dev Ctries 2012; 6(10): 704-14.	Initial systematic review			✓		✓	
Khanal B, Sharma SK, Amatya R, Poudyal N. *Salmonella* Typhi and Paratyphi A in Eastern Nepal-Trends in Antimicrobial Resistance. Int J Infect Dis 2008; 12: E109-E.	Initial systematic review				✓		
Khanal B, Sharma SK, Bhattacharya SK, Bhattarai NR, Deb M, Kanungo R. Antimicrobial susceptibility patterns of *Salmonella* enterica serotype Typhi in eastern Nepal. J Health Popul Nutr 2007; 25(1): 82-7.	Initial systematic review	✓	✓			✓	
Khanal PR, Satyal D, Bhetwal A, et al. Renaissance of conventional first-line antibiotics in *Salmonella* enterica clinical isolates: Assessment of MICs for therapeutic antimicrobials in enteric fever cases from Nepal. Biomed Res Int 2017; 2017: 2868143.	Initial systematic review	✓	✓	✓	✓	✓	✓

Khanam F, Qadri F. Characterization of the induced immune responses, clinical presentation and antibiotic susceptibility pattern in Bangladeshi S. Paratyphi A bacteremic patients. Int J Infect Dis 2014; 21: 343.	Initial systematic review				✓		✓
Khichi QK, Channer MS. Parenteral chloramphenicol versus chloramphenicol plus dexamethasone in the treatment of typhoid fever. Pak Paed J, 2001.	Extended systematic review	✓					
Khosla SN, Saman A, Khosla P, Sabharwal U, Khosla A. Drug resistant typhoid fever. Trop Doct 1998; 28(4): 235-7.	Extended systematic review	✓					
Ki-Zerbo GA, Sawadogo AB, Kyelem N, Zoubga A, Thiombiano R, Durand G. Enterobacteriaceae bacteriemia in human deficiency virus seropositive in patients at Bobo-Dioulasso hospital (Burkina Faso): Study of 26 cases. Med Mal Infect 2000; 30(12): 753-6.	Initial systematic review	✓					
Koirala S, Basnyat B, Arjyal A, et al. Gatifloxacin versus ofloxacin for the treatment of uncomplicated enteric fever in Nepal: an open-label, randomized, controlled trial. PLoS Negl Trop Dis 2013; 7(10): e2523.	Initial systematic review	✓		✓	✓	✓	✓
Komolpis P, Srifuengfung S, Dhiraputra C, Pingwang B. *Salmonella* bacteremia: serotype distribution and antimicrobial susceptibility during 1991-1995. Journal of Infectious Diseases and Antimicrobial Agents 1999; 16(2): 49-52.	Initial systematic review	✓		✓		✓	✓
Kong X, Dong C, Ji H, et al. Antimicrobial resistance and molecular subtypes of *Salmonella* Typhi and *Salmonella* paratyphoid isolates from Jiangsu province, 2012-2015. [Chinese]. Chinese Journal of Endemiology 2017; 38(11): 1546-50.	Extended systematic review		✓	✓	✓		
Koul PB, Murali MV, Sharma PP, Ghai OP, Ramchandran VG, Talwar V. Multi drug resistant *Salmonella* Typhi infection: clinical profile and therapy. Indian Pediatr 1991; 28(4): 357-61.	Initial systematic review	✓					
Kownhar H, Shankar EM, Rajan R, Rao UA. Emergence of nalidixic acid-resistant *Salmonella* enterica serovar Typhi resistant to ciprofloxacin in India. J Med Microbiol 2007; 56(Pt 1): 136-7.	Extended systematic review	✓	✓			✓	
Kuijpers LMF, Phe T, Veng CH, et al. The clinical and microbiological characteristics of enteric fever in Cambodia, 2008-2015. PLoS Negl Trop Dis 2017; 11(9): e0005964.	Initial systematic review	✓	✓	✓	✓	✓	✓

Kulkarni SA, Kulkarni AG. Multi resistant *Salmonella* Typhi from rural southern Maharashtra. J Assoc Physicians India 1992; 40(9): 636-7.	Extended systematic review	✓					
Kumar MS, Kumar GSV, Prashanth HV, Prakash R, Veena K, Chandrashekar SC. Antimicrobial susceptibility of enteric fever *Salmonella*e isolated from blood culture. Res J Pharm Biol Chem Sci 2013b; 4(3): 318-24.	Initial systematic review	✓	✓	✓	✓	✓	✓
Kumar R, Aneja KR, Punia AK, et al. Changing pattern of biotypes, phage types & drug resistance of *Salmonella* Typhi in Ludhiana during 1980-1999. Indian J Med Res 2001; 113: 175-80.	Initial systematic review	✓					
Kumar R, Aneja KR, Roy P, Sharma M, Gupta R, Ram S. Evaluation of minimum inhibitory concentration of quinolones and third generation cephalosporins to *Salmonella* Typhi isolates. Indian J Med Sci 2002; 56(1): 1-8.	Initial systematic review	✓					
Kumar R, Gupta N, Shalini. Multidrug-resistant typhoid fever. Indian J Pediatr 2007; 74(1): 39-42.	Initial systematic review	✓				✓	
Kumar S, Rizvi M, Berry N. Rising prevalence of enteric fever due to multidrug-resistant *Salmonella*: an epidemiological study. J Med Microbiol 2008; 57(Pt 10): 1247-50.	Initial systematic review	✓	✓	✓		✓	✓
Kumar Y, Sharma A, Mani KR. Antibiogram profile of *Salmonella* enterica serovar Typhi in India - A two year study. Trop Life Sci Res 2013a; 24(1): 45-54.	Initial systematic review	✓	✓				
Kumar Y, Sharma A, Mani KR. High level of resistance to nalidixic acid in *Salmonella* enterica serovar Typhi in Central India. J Infect Dev Ctries 2009; 3(6): 467-9.	Initial systematic review		✓				
Kumar Y, Sharma A, Mani KR. Re-emergence of susceptibility to conventionally used drugs among strains of *Salmonella* Typhi in central west India. J Infect Dev Ctries 2011; 5(3): 227-30.	Extended systematic review	✓					
Kuzucu C, Baktr E, Acar N. Antibiotic susceptibilities of *Salmonella* and Shigella strains isolated between 1998-1999. [Turkish]. Turk Hijyen ve Deneysel Biyoloji Dergisi 2001; 58(1): 11-4.	Extended systematic review	✓					

Kwambana-Adams B, Darboe S, Nabwera H, et al. *Salmonella* Infections in The Gambia, 2005-2015. Clin Infect Dis 2015; 61: S354-S62.	Extended systematic review	✓				✓	
Lakshmi V, Ashok R, Susmita J, Shailaja VV. Changing trends in the antibiograms of *Salmonella* isolates at a tertiary care hospital in Hyderabad. Indian J Med Microbiol 2006; 24(1): 45-8.	Initial systematic review	✓	✓	✓	✓	✓	✓
Lari AR, Validi N, Ghaffarzadeh K, Shamshiri AR. In vitro activity of cefixime versus ceftizoxime against *Salmonella* Typhi. Pathologie Biologie 1997; 45(5): 415-9.	Extended systematic review	✓				✓	
Lee C, Su L, Huang Y, Chiu C. First isolation of ciprofloxacin-resistant *Salmonella* enterica serovar Typhi in Taiwan. Journal of Microbiology, Immunology and Infection 2013; 46(6): 469-73.	Extended systematic review	✓				✓	
Lefebvre N, Gning SB, Nabeth P, et al. Clinical and laboratory features of typhoid fever in Senegal. A 70-case study [French]. Med Trop 2005; 65(6): 543-8.	Initial systematic review	✓	✓				
Leung DT, Bogetz J, Itoh M, et al. Factors associated with encephalopathy in patients with *Salmonella* enterica serotype Typhi bacteremia presenting to a diarrheal hospital in Dhaka, Bangladesh. Am J Trop Med Hyg 2012; 86(4): 698-702.	Initial systematic review	✓					
Lewis MD, Serichantalergs O, Pitarangsi C, et al. Typhoid fever: a massive, single-point source, multidrug-resistant outbreak in Nepal. Clin Infect Dis 2005; 40(4): 554-61.	Initial systematic review	✓	✓			✓	
Limpitikul W, Henpraserttae N, Saksawad R, Laoprasopwattana K. Typhoid outbreak in Songkhla, Thailand 2009-2011: Clinical outcomes, susceptibility patterns, and reliability of serology tests. PLoS ONE 2014; 9(11).	Initial systematic review	✓				✓	
Ling JH, Wang Y, Li JJ, Xu YC, Xie XL, Chen MJ. Comparison of antimicrobial susceptability of common gram-negative isolates from blood stream between Hong Kong and Beijing patients. [Chinese]. Chinese Journal of Infection and Chemotherapy 2006; 6(4): 251-4.	Initial systematic review	✓				✓	
Ling JM, Lo NW, Ho YM, et al. Molecular methods for the epidemiological typing of *Salmonella* enterica serotype Typhi from Hong Kong and Vietnam. J Clin Microbiol 2000; 38(1): 292-300.	Extended systematic review	✓					

Lo NWS, Chu MT, Ling JM. Increasing quinolone resistance and multidrug resistant isolates among *Salmonella* enterica in Hong Kong. J Infect 2012; 65(6): 528-40.	Extended systematic review	✓	✓	✓		✓	✓
Lunguya O, Lejon V, Phoba MF, et al. *Salmonella* Typhi in the Democratic Republic of the Congo: Fluoroquinolone decreased susceptibility on the rise. PLoS Negl Trop Dis 2012; 6(11): e1921.	Initial systematic review	✓	✓				
Ly N, Chinh N, Parry C, Diep T, Wain J, White N. Randomised trial of azithromycin versus ofloxacin for the treatment of typhoid fever in adult. Med J Indones 1998; 7: 202-6.	Initial systematic review	✓	✓				
Mache A, Mengistu Y, Cowley S. *Salmonella* serogroups identified from adult diarrhoeal out-patients in Addis Ababa, Ethiopia: Antibiotic resistance and plasmid profile analysis. East Afr Med J 1997; 74(3): 183-6.	Extended systematic review	✓					
Madhulika U, Harish BN, Parija SC. Current pattern in antimicrobial susceptibility of *Salmonella* Typhi isolates in Pondicherry. Indian J Med Res 2004; 120(2): 111-4.	Initial systematic review	✓	✓			✓	
Mahanta J. Drug sensitivity of *Salmonella* Paratyphi A isolated from a suspected outbreak of enteric fever in Duliajan. J Indian Med Assoc 1994; 92(2): 49-50.	Extended systematic review			✓			
Mahapatra A, Patro S, Choudhury S, Padhee A, Das R. Emerging enteric fever due to switching biotype of *Salmonella* (Paratyphi A) in Eastern Odisha. Indian J Pathol Microbiol 2016; 59(3): 327-9.	Initial systematic review			✓	✓		
Mahende C, Ngasala B, Lusingu J, et al. Bloodstream bacterial infection among outpatient children with acute febrile illness in north-eastern Tanzania. BMC Res Notes 2015; 8: 289.	Initial systematic review	✓	✓			✓	
Maheshwari VD, Agarwal SK. Present status of drug resistance in cases of enteric fever in Rajasthan. J Assoc Physicians India 1996; 44(9): 618-9.	Initial systematic review	✓				✓	
Mahmud ASM, Hakim MA. Prevalence and antimicrobial resistance of *Salmonella* strains isolated from blood cultures in Dhaka Division, Bangladesh. J Pure Appl Microbiol 2014; 8(3): 2315-23.	Initial systematic review		✓				

Makkar A, Gupta S, Khan ID, et al. Epidemiological Profile and Antimicrobial Resistance Pattern of Enteric Fever in a Tertiary Care Hospital of North India - a Seven Year Ambispective Study.Acta Medica (Hradec Kralove) 2018;61(4):125-130	Initial systematic review	✓	✓	✓	✓	✓	✓
Malik N, Ahmed M. In vitro effect of new antibiotics against clinical isolates of *Salmonella* Typhi. J Coll Physicians Surg Pak 2016; 26(4): 288-92.	Extended systematic review	✓	✓			✓	
Malla S, Kansakar P, Serichantalergs, Rahman M, Basnet S. Epidemiology of typhoid and paratyphoid fever in Kathmandu: two years study and trends of antimicrobial resistance. JNMA J Nepal Med Assoc 2005; 44(157): 18-22.	Initial systematic review	✓		✓			
Malla T, Malla KK, Thapalial A, Shaw C. Enteric fever: a retrospective 6-year analysis of 82 paediatric cases in a teaching hospital. Kathmandu Univ Med J (KUMJ) 2007; 5(2): 181-7.	Extended systematic review	✓				✓	
Maltha J, Guiraud I, Kabore B, et al. Frequency of severe malaria and invasive bacterial infections among children admitted to a rural hospital in Burkina Faso. PLoS ONE 2014; 9(2).	Initial systematic review	✓	✓				
Mamun KZ, Tabassum S, Ashna SM, Hart CA. Molecular analysis of multi-drug resistant *Salmonella* Typhi from urban paediatric population of Bangladesh. Bangladesh Med Res Counc Bull 2004; 30(3): 81-6.	Initial systematic review	✓	✓				
Manchanda V, Bhalla P, Sethi M, Sharma VK. Treatment of enteric fever in children on the basis of current trends of antimicrobial susceptibility of *Salmonella* enterica serovar Typhi and Paratyphi A. Indian J Med Microbiol 2006; 24(2): 101-6.	Initial systematic review	✓	✓	✓		✓	
Mandal S, Debmandal M, Pal NK. Antibiotic resistance of *Salmonella* enterica serovar Typhi in Kolkata, India, and in vitro experiments on effect of combined chemotherapy. ScientificWorldJournal 2012; 2012(454059).	Initial systematic review		✓			✓	
Mandal S, Mandal M, Pal N. Antibiotic resistance of *Salmonella* enterica serovar Paratyphi A in India: Emerging and reemerging problem. J Postgrad Med 2006; 52(3): 163-6.	Initial systematic review			✓	✓		✓
Mandal S, Mandal MD, Pal NK. Antimicrobial resistance pattern of *Salmonella* Typhi isolates in Kolkata, India during 1991-2001: a retrospective study. Jpn J Infect Dis 2002; 55(2): 58-9.	Initial systematic review	✓					

Mandal S, Mandal MD, Pal NK. Reduced minimum inhibitory concentration of chloramphenicol for *Salmonella* enterica serovar Typhi. Indian J Med Sci 2004; 58(1): 16-23.	Initial systematic review	✓	✓			✓	
Mandeep W, Rajni G, Premila P, Rajesh M, Pushpa A, Mani K. Age-related clinical and microbiological characteristics of enteric fever in India. Trans R Soc Trop Med Hyg 2006; 100(10): 942-8.	Initial systematic review	✓	✓	✓	✓		
Marks F, Adu-Sarkodie Y, Hunger F, et al. Typhoid fever among children, ghana. Emerg Infect Dis 2010; 16(11): 1796-7.	Initial systematic review	✓				✓	
Marks F, Kalckreuth V, Aaby P, et al. Incidence of invasive *Salmonella* disease in sub-Saharan Africa: A multicentre population-based surveillance study. Lancet Glob Health 2017; 5(3): e310-e23.	Initial systematic review	✓	✓			✓	
Mashe T, Mugabe M, Stray-Pedersen B, et al. Antibiotic resistance trends of *Salmonella* enterica serotype Typhi in Zimbabwe (2012-2017). Antimicrobial Resistance and Infection Control Conference: International Conference on Prevention and Infection Control, ICPIC 2017; 6(Supplement 3).	Extended systematic review	✓				✓	
Maskey AP, Basnyat B, Thwaites GE, Campbell JI, Farrar JJ, Zimmerman MD. Emerging trends in enteric fever in Nepal: 9124 cases confirmed by blood culture 1993-2003. Trans R Soc Trop Med Hyg 2008; 102(1): 91-5.	Initial systematic review			✓			
Maskey AP, Day JN, Phung QT, et al. *Salmonella* enterica serovar Paratyphi A and S. enterica serovar Typhi cause indistinguishable clinical syndromes in Kathmandu, Nepal. Clin Infect Dis 2006; 42(9): 1247-53.	Initial systematic review	✓	✓	✓	✓	✓	✓
Mathew R, Jobin SR. Current trends of antimicrobial susceptibility of *Salmonella* enterica serovar Typhi and Paratyphi a from blood cultures in a tertiary care center of Chennai, Tamil Nadu, India. Res J Pharm Biol Chem Sci 2014; 5(2): 1167-74.	Initial systematic review	✓	✓	✓	✓	✓	✓
Mathur YC, Mathur NC, Lal HM. Clinical efficacy of Cefuroxime axetil in S. Typhi. Indian Pediatr 1996; 33(12): 1033-7.	Initial systematic review	✓					
Mathura KC, Chaudhary D, Simkhada R, Pradhan M, Shrestha P, Gurubacharya DL. Study of clinical profile and antibiotic sensitivity pattern in culture positive typhoid fever cases. Kathmandu Univ Med J (KUMJ) 2005; 3(4): 376-9.	Initial systematic review	✓				✓	

Mayxay M, Castonguay-Vanier J, Chansamouth V, et al. Causes of non-malarial fever in Laos: a prospective study. Lancet Glob Health 2013; 1(1): e46-54.	Initial systematic review	✓	✓			✓	
Mehta A, Rodriques C, Joshi VR. Multiresistant *Salmonella* organisms in India. JAMA 1992; 267(12): 1614.	Initial systematic review	✓					
Mehta M, Joshi RM. Antibiogram pattern of *Salmonella* species causing bacteraemia/septicaemia in a teaching hospital. J Infect 2002; 45(2): 133.	Initial systematic review	✓		✓		✓	✓
Meiring J, Shakya M, Khanam F, et al. Burden of enteric fever at three urban sites in Africa and Asia: a multicentre population-based study. Lancet Glob Health 2021; 9(12): e1688 - e1696	Dataset	✓	✓	✓	✓	✓	✓
Menezes GA, Harish BN, Khan MA, Goessens W, Hays JP. Antimicrobial resistance trends in blood culture positive *Salmonella* Paratyphi A isolates from Pondicherry, India. Indian J Med Microbiol 2016; 34(2): 222-7.	Initial systematic review			✓	✓		✓
Menezes GA, Harish BN, Khan MA, Goessens WHF, Hays JP. Antimicrobial resistance trends in blood culture positive *Salmonella* Typhi isolates from Pondicherry, India, 2005-2009. Clin Microbiol Infect 2012; 18(3): 239-45.	Initial systematic review	✓	✓			✓	
Mengo DM, Kariuki S, Muigai A, Revathi G. Trends in *Salmonella* enteric serovar Typhi in Nairobi, Kenya from 2004 to 2006. J Infect Dev Ctries 2010; 4(6): 393-6.	Initial systematic review	✓	✓			✓	
Mills-Robertson F, Addy ME, Mensah P, Crupper SS. Molecular characterization of antibiotic resistance in clinical *Salmonella* Typhi isolated in Ghana. FEMS Microbiology Letters 2002; 215(2): 249-53.	Extended systematic review	✓					
Mills-Robertson F, Crupper SS, Addy ME, Mensah P. Antibiotic resistance and genotyping of clinical group B *Salmonella* isolated in Accra, Ghana. J Appl Microbiol 2003; 94(2): 289-94.	Extended systematic review	✓					
Ministry of Health Malaysia. National antibiotic resistance surveillance report 2007, 2007.	Surveillance report					✓	
Ministry of Health Malaysia. National antibiotic resistance surveillance report 2012, 2012.	Surveillance report	✓				✓	
Ministry of Health Malaysia. National antibiotic resistance surveillance report 2016, 2016.	Surveillance report	✓				✓	

Mirza SH, Beeching NJ, Hart CA. The prevalence and clinical features of multi-drug resistant *Salmonella* Typhi infections in Baluchistan, Pakistan. Ann Trop Med Parasitol 1995; 89(5): 515-9.	Initial systematic review	✓		✓			
Mirza SH, Khan MA. Low-level quinolone-resistance in multi-drug resistant typhoid. J Coll Physicians Surg Pak 2008; 18(1): 13-6.	Initial systematic review	✓	✓				
Mishra OP, Gupta BL, Nath G, Prakash J. Treatment of multidrug-resistant typhoid fever. J Trop Pediatr 1996; 42(5): 310-1.	Initial systematic review	✓				✓	
Mishra S, Patwari AK, Anand VK, et al. Multidrug resistant typhoid fever: therapeutic considerations. Indian Pediatr 1992; 29(4): 443-8.	Initial systematic review	✓				✓	
Misra R, Thakare R, Amrin N, Prasad KN, Chopra S, Dhole TN. Antimicrobial susceptibility pattern and sequence analysis of DNA gyrase and DNA topoisomerase IV in *Salmonella* enterica serovars Typhi and Paratyphi A isolates with decreased susceptibility to ciprofloxacin. Trans R Soc Trop Med Hyg 2016; 110(8): 472-9.	Initial systematic review	✓	✓	✓	✓	✓	✓
Misra RN, Bawa KS, Magu SK, Bhandari S, Nagendra A, Menon PK. Outbreak of multi-drug resistant *Salmonella* Typhi enteric fever in Mumbai garrison. Med J Armed Forces India 2005; 61(2): 148-50.	Initial systematic review	✓	✓			✓	
Moehario LH, Tjoa E, Kiranasari A, Ningsih I, Rosana Y, Karuniawati A. Trends in antimicrobial susceptibility of gram-negative bacteria isolated from blood in Jakarta from 2002 to 2008. J Infect Dev Ctries 2009; 3(11): 843-8.	Initial systematic review					✓	
Mohanty S, Renuka K, Sood S, Das BK, Kapil A. Antibiogram pattern and seasonality of *Salmonella* serotypes in a North Indian tertiary care hospital. Epidemiol Infect 2006; 134(5): 961-6.	Initial systematic review	✓	✓	✓	✓	✓	✓
Mourad AS, Metwally M, el Deen AN, et al. Multiple-drug-resistant *Salmonella* Typhi. Clin Infect Dis 1993; 17(1): 135-6.	Extended systematic review	✓				✓	
Muhammad R, Ali Z, Mehmood K, Ziauddin, Afridi AR, Bari F. Antibiotic sensitivity pattern of *Salmonella* serotypes in patients with enteric fever in a teaching hospital. Journal of Postgraduate Medical Institute 2013; 27(4): 397-402.	Initial systematic review	✓		✓		✓	✓

Mukherjee P, Mukherjee S, Dalal BK, Haldar KK, Ghosh E, Pal TK. Some prospective observations on recent outbreak of typhoid fever in West Bengal.[Erratum appears in J Assoc Physicians India. 1992 Apr;40(4):285-6; PMID: 1452554]. J Assoc Physicians India 1991; 39(6): 445-8.	Extended systematic review	✓					
Mushtaq MA. What after ciprofloxacin and ceftriaxone in treatment of *Salmonella* Typhi. Pak J Med Sci 2006; 22(1): 51-4.	Initial systematic review	✓				✓	
Musicha P, Feasey N. BC_data_2015_16.2. Blantrye, Malawi; 2019.	Dataset	✓					
Musicha P, Feasey N. MLWBC1998_2014. Blantyre, Malawi; 2019.	Dataset	✓				✓	
Mutai WC, Muigai AWT, Waiyaki P and Kariuki S. Multi-drug resistant *Salmonella* enterica serovar Typhi isolates with reduced susceptibility to ciprofloxacin in Kenya.BMC Microbiology 2018;18(1):187	Initial systematic review	✓	✓			✓	
Muthu G, Suresh A, Elumalaisathishkumar, et al. Molecular analysis of gyramutations in *Salmonella* Paratyphi ABY PCR-RFLP and sequencing method. International Journal of Pharmacy and Pharmaceutical Sciences 2014; 6(4): 658-60.	Extended systematic review			✓	✓		✓
Muyembe-Tamfum JJ, Veyi J, Kaswa M, Lunguya O, Verhaegen J, Boelaert M. An outbreak of peritonitis caused by multidrug-resistant *Salmonella* Typhi in Kinshasa, Democratic Republic of Congo. Travel Med Infect Dis 2009; 7(1): 40-3.	Initial systematic review	✓	✓				
Narain U, Gupta R. Emergence of resistance in community-acquired enteric fever. Indian Pediatr 2015; 52(8): 709-.	Initial systematic review	✓				✓	
Narasanna R, Chavadi M and Chandrakanth K. Prevalence of multidrug-resistant *Salmonella* Typhi in typhoid patients and detection of blaCTX-M2 and blaCTX-M9 genes in cefetoxime-mediated extended spectrum beta-lactamase-producing *Salmonella* Typhi isolates.Biomedical Research (India) 2018;29(14):3015-3021	Initial systematic review	✓	✓			✓	
Nath G, Maurya P. Drug resistance patterns in *Salmonella* enterica subspecies enterica serotype Typhi strains isolated over a period of two decades, with special reference to ciprofloxacin and ceftriaxone. Int J Antimicrob Agents 2010; 35(5): 482-5.	Extended systematic review	✓	✓			✓	
Nath G, Tikoo A, Manocha H, Tripathi AK, Gulati AK. Drug resistance in *Salmonella* Typhi in north India with special reference to ciprofloxacin. J Antimicrob Chemother 2000; 46(1): 149-50.	Extended systematic review					✓	

Neil KP, Sodha SV, Lukwago L, et al. A large outbreak of typhoid fever associated with a high rate of intestinal perforation in Kasese District, Uganda, 2008-2009. Clin Infect Dis 2012; 54(8): 1091-9.	Extended systematic review	✓	✓				
Neopane A, Singh SB, Bhatta R, Dhital B, Karki DB. Changing spectrum of antibiotic sensitivity in enteric fever. Kathmandu Univ Med J (KUMJ) 2008; 6(1): 12-5.	Initial systematic review	✓	✓			✓	
Newman MJ, Frimpong E, Donkor ES, Opintan JA, Asamoah-Adu A. Resistance to antimicrobial drugs in Ghana. Infect Drug Resist 2011; 4(1): 215-20.	Extended systematic review	✓	✓				
Nga TVT, Parry CM, Le T, et al. The decline of typhoid and the rise of non-typhoid *Salmonella*e and fungal infections in a changing HIV landscape: Bloodstream infection trends over 15 years in southern Vietnam. Trans R Soc Trop Med Hyg 2012; 106(1): 26-34.	Initial systematic review	✓	✓				
Ngoun C, Emary K, Khun PA, et al. Enteric fever in Cambodian children is dominated by multidrug resistant H58 *Salmonella* enterica serovar Typhi with decreased susceptibility to ciprofloxacin. Int J Infect Dis 2012; 16: e427.	Initial systematic review	✓	✓			✓	
Nguyen Dac T, Suthisarnsuntorn U, Kalambaheti T, Wonglumsom W, Tunyong W. Antimicrobial susceptibility patterns and phage types of *Salmonella* Typhi from Vietnam. Southeast Asian J Trop Med Public Health 2007; 38(3): 487-92.	Extended systematic review	✓	✓			✓	
Nguyen TA, Ha Ba K, Nguyen TD. Typhoid fever in South Vietnam, 1990-1993. [French]. Bulletin de la Societe de pathologie exotique (1990) 1993; 86(5 Pt 2): 476-8.	Extended systematic review			✓		✓	✓
Nobthai P, Serichantalergs O, Wongstitwilairoong B, et al. Emergence and properties of fluoroquinolone resistant *Salmonella* enterica serovar Typhi strains isolated from Nepal in 2002 and 2003. Southeast Asian J Trop Med Public Health 2010; 41(6): 1416-22.	Extended systematic review	✓	✓				
Obaro S, Lawson L, Essen U, et al. Community acquired bacteremia in young children from central Nigeria - a pilot study. BMC Infect Dis 2011; 11(137).	Initial systematic review	✓				✓	
Ochiai RL, Acosta CJ, Danovaro-Holliday MC, et al. A study of typhoid fever in five Asian countries: Disease burden and implications for controls. Bull World Health Organ 2008; 86(4): 260-8.	Initial systematic review	✓	✓			✓	

Okanda T, Haque A, Ehara T, et al. Characteristics of resistance mechanisms and molecular epidemiology of fluoroquinolone-nonsusceptible *Salmonella* enterica serovar Typhi and Paratyphi a isolates from a tertiary hospital in Dhaka, Bangladesh.Microbial Drug Resistance 2018;24(10):1460-1465	Initial systematic review	✓	✓	✓	✓	✓	✓
Okome-Nkoumou M, Ayo Nkana E, Bekale J, Kombila M. [Typhoid and paratyphoid fever in adults in the Internal Medicine Department at Libreville (Gabon)]. Sante 2000; 10(3): 205-9.	Extended systematic review	✓		✓			
Okwori EE, Nwadioha SI, Nwokedi EOP, Odimayo M, Jombo G. Bacterial pathogens and their antimicrobial susceptibility in Otukpo Benue state of Nigeria. Asian Pacific Journal of Tropical Biomedicine 2011; 2(12).	Extended systematic review	✓		✓		✓	
Olasupo NA, Alabi SA, Akinyemi KA, Omonigbehin EA. The antimicrobial susceptibility pattern of bacterial agents isolated from patients with diarrhoea. Biomedical Letters 1999; 60(235): 77-82.	Extended systematic review		✓				
Olsen SJ, Pruckler J, Bibb W, et al. Evaluation of rapid diagnostic tests for typhoid fever. J Clin Microbiol 2004; 42(5): 1885-9.	Initial systematic review	✓	✓				
Onken A, Said AK, Jorstad M, Jenum PA, Blomberg B. Prevalence and Antimicrobial Resistance of Microbes Causing Bloodstream Infections in Unguja, Zanzibar. PLoS ONE 2015; 10(12).	Extended systematic review	✓					
Onyango D, Machioni F, Kakai R, Waindi EN. Multidrug resistance of *Salmonella* enterica serovars Typhi and Typhimurium isolated from clinical samples at two rural hospitals in Western Kenya. J Infect Dev Ctries 2008; 2(2): 106-11.	Extended systematic review	✓					
Oyofo BA, Lesmana M, Subekti D, et al. Surveillance of bacterial pathogens of diarrhea disease in Indonesia. Diagn Microbiol Infect Dis 2002; 44(3): 227-34.	Extended systematic review	✓	✓			✓	
Pais N, Ravindranath S, Kesavamurthy. Drug resistant typhoid in Bangalore. J Assoc Physicians India 1991; 39(8): 654.	Initial systematic review	✓					
Pakistan Antimicrobial Resistance Network. Antimicrobial data. 2018. https://parn.org.pk/antimicrobial-data/.	Surveillance report	✓		✓		✓	✓

Pal N, Ayyagari A, Panigrahi D. Outbreak of multidrug resistant S. Typhi infection. Bull Postgrad Inst Med Educ Res Chandigarh 1991; 25(4): 186-90.	Initial systematic review	✓			
Pallab R, Jyoti S, Marak RSK, Garg RK. Predictive efficacy of nalidixic acid resistance as a marker of fluoroquinolone resistance in *Salmonella* enterica var Typhi. Indian J Med Res 2006; 124(1): 105-8.	Initial systematic review	✓	✓		
Pandit V, Kumar A, Kulkarni MM, Pattanshetty SM, Samarasinghe C, Kamath S. Study of clinical profile and antibiotic sensitivity in paratyphoid fever cases admitted at teaching hospital in South India. J Family Med Prim Care 2012; 1(2): 118-21.	Initial systematic review			✓	
Panhotra BR, Saxena AK, Al-Ghamdi AM. Typhoid fever due to multiresistant *Salmonella* enterica serovar Typhi having reduced susceptibility to ciprofloxacin and nalidixic acid resistance. Saudi Med J 2004; 25(10): 1509-11.	Initial systematic review	✓	✓		✓
Panigrahi D, Roy P, Sehgal R. Ciprofloxacin for typhoid fever? [28]. Lancet 1991; 338(8782-8783): 1601.	Extended systematic review	✓			
Parry C, Wain J, Chinh NT, Vinh H, Farrar JJ. Quinolone-resistant *Salmonella* Typhi in Vietnam. Lancet 1998; 351(9111): 1289.	Initial systematic review		✓		
Parry CM, Ho VA, Phuong le T, et al. Randomized controlled comparison of ofloxacin, azithromycin, and an ofloxacin-azithromycin combination for treatment of multidrug-resistant and nalidixic acid-resistant typhoid fever. Antimicrob Agents Chemother 2007; 51(3): 819-25.	Extended systematic review	✓	✓		✓
Parwati I, Samaun E. Multidrug resistance *Salmonella* in dr. Hasan Sadikin general hospital, Bandung. Med J Indones 1998; 7: 194.	Extended systematic review	✓		✓	
Pathak A, Marothi Y, Kekre V, Mahadik K, Macaden R, Lundborg CS. High prevalence of extended-spectrum beta-lactamase-producing pathogens: results of a surveillance study in two hospitals in Ujjain, India. Infect Drug Resist 2012; 5: 65-73.	Initial systematic review	✓	✓		✓
Paul K, De PK. Transferable chloramphenicol resistance in *Salmonella* Typhi isolated during an outbreak of enteric fever in Calcutta, West Bengal. Indian J Med Microbiol 1991; 9(1): 10-2.	Extended systematic review	✓			

Petersiel N, Shresta S, Tamrakar R, et al. The epidemiology of typhoid fever in the Dhulikhel area, Nepal: A prospective cohort study.PLoS ONE 2018;13 (9) (no pagination)(e0204479):	Initial systematic review	✓	✓	✓	✓	✓	✓
Phoba MF, De Boeck H, Ifeka BB, et al. Epidemic increase in *Salmonella* bloodstream infection in children, Bwamanda, the Democratic Republic of Congo. Eur J Clin Microbiol Infect Dis 2014; 33(1): 79-87.	Initial systematic review	✓					
Phoba MF, Lunguya O, Mayimon DV, et al. Multidrug-resistant *Salmonella* enterica, Democratic Republic of the Congo. Emerg Infect Dis 2012; 18(10): 1693-4.	Initial systematic review	✓	✓				
Phuong CXT, Kneen R, Anh NT, Luat TD, White NJ, Parry CM. A comparative study of ofloxacin and cefixime for treatment of typhoid fever in children. Pediatr Infect Dis J 1999; 18(3): 245-8.	Initial systematic review	✓	✓			✓	
Pillai PK, Prakash K. Current status of drug resistance and phage types of *Salmonella* Typhi in India. Indian J Med Res 1993; 97(July): 154-8.	Extended systematic review	✓					
Pourshafie MR, Saifi M, Mousavi SF, Sedaghat M, Nikbakht GH, Rubino S. Clonal diversity of *Salmonella* enterica serotype Typhi isolated from patients with typhoid fever in Tehran. Scandinavian Journal of Infectious Diseases 2008; 40(1): 18-23.	Extended systematic review	✓	✓			✓	
Pradhan R, Shrestha U, Gautam SC, et al. Bloodstream infection among children presenting to a general hospital outpatient clinic in urban Nepal. PLoS ONE 2012; 7(10): e47531.	Initial systematic review		✓		✓		
Prajapati B, Rai GK, Rai SK, et al. Prevalence of *Salmonella* Typhi and Paratyphi infection in children: a hospital based study. Nepal Med Coll J 2008; 10(4): 238-41.	Initial systematic review	✓		✓		✓	✓
Pratap CB, Patel SK, Shukla VK, Tripathi SK, Singh TB, Nath G. Drug resistance in *Salmonella* enterica serotype Typhi isolated from chronic typhoid carriers. Int J Antimicrob Agents 2012; 40(3): 279-80.	Extended systematic review	✓	✓			✓	
Punjabi NH, Agtini MD, Ochiai RL, et al. Enteric fever burden in North Jakarta, Indonesia: a prospective, community-based study. J Infect Dev Ctries 2013; 7(11): 781-7.	Initial systematic review	✓	✓	✓	✓	✓	✓

Punjabi NH, Taylor WR, Murphy GS, et al. Etiology of acute, non-malaria, febrile illnesses in Jayapura, northeastern Papua, Indonesia. Am J Trop Med Hyg 2012; 86(1): 46-51.	Initial systematic review	✓					
Purighalla S, Esakimuthu S, Reddy M, et al. Investigation into a community outbreak of *Salmonella* Typhi in Bengaluru, India.Indian Journal of Medical Research 2017;146(July Supplement):15-22	Initial systematic review	✓	✓			✓	
Qadri SM, Ueno Y, Almodovar E. In vitro activity of sparfloxacin against *Salmonella* Typhi. Chemotherapy 1997; 43(1): 6-9.	Extended systematic review	✓					
Qaiser S, Irfan S, Khan E, Ahsan T, Zafar A. In vitro susceptibility of typhoidal *Salmonella*e against newer antimicrobial agents: a search for alternate treatment options. J Pak Med Assoc 2011; 61(5): 462-5.	Initial systematic review					✓	✓
Qamar FN, Asma A, Kazi AM, Erum K, Zaidi AKM. A three-year review of antimicrobial resistance of *Salmonella* enterica serovars Typhi and Paratyphi A in Pakistan. J Infect Dev Ctries 2014; 8(8): 981-6.	Initial systematic review	✓		✓		✓	✓
Qamar FN, Yousafzai MT, Sultana S, et al. A Retrospective Study of Laboratory-Based Enteric Fever Surveillance, Pakistan, 2012-2014.Journal of Infectious Diseases 2018;218(suppl_4):S201-S205	Initial systematic review	✓		✓		✓	✓
Qureshi AH, Mushahid N, Ijaz A, et al. Changing drug susceptibility pattern of *Salmonella*e Paratyphi A. J Coll Physicians Surg Pak 2001; 11(7): 449-51.	Initial systematic review			✓			✓
Raghu Raman TS, Krishnamurthy L, Menon PK, Singh D, Jayaprakash DG. Clinical profile and therapy in enteric fever. Indian Pediatr 1994; 31(2): 196-9.	Extended systematic review	✓					
Rahman BA, Wasfy MO, Maksoud MA, Hanna N, Dueger E, House B. Multi-drug resistance and reduced susceptibility to ciprofloxacin among *Salmonella* enterica serovar Typhi isolates from the Middle East and Central Asia. New microbes new infect 2014; 2(4): 88-92.	Initial systematic review	✓	✓			✓	
Rahman M, Ahmad A, Shoma S. Decline in epidemic of multidrug resistant *Salmonella* Typhi is not associated with increased incidence of antibiotic-susceptible strain in Bangladesh. Epidemiol Infect 2002; 129(1): 29-34.	Initial systematic review	✓				✓	

Rahman M, Siddique AK, Shoma S, et al. Emergence of multidrug-resistant *Salmonella* enterica serotype Typhi with decreased ciprofloxacin susceptibility in Bangladesh. Epidemiol Infect 2006; 134(2): 433-8.	Initial systematic review	✓	✓			✓	
Rahman MM, Haq JA, Morshed MA, Rahman MA. *Salmonella* enterica serovar Typhi with decreased susceptibility to ciprofloxacin--an emerging problem in Bangladesh. Int J Antimicrob Agents 2005; 25(4): 345-6.	Initial systematic review	✓	✓				
Rai GK, Karki S, Prajapati B. Is antimicrobial resistance pattern of enteric fever changing in Kathmandu valley? Journal of Nepal Paediatric Society 2012a; 32(3): 221-8.	Initial systematic review	✓	✓		✓	✓	✓
Rai S, Jain S, Prasad KN, Ghoshal U, Dhole TN. Rationale of azithromycin prescribing practices for enteric fever in India. Indian J Med Microbiol 2012b; 30(1): 30-3.	Initial systematic review	✓	✓	✓	✓		
Rajajee S, Anandi TB, Subha S, Vatsala BR. Patterns of resistant *Salmonella* Typhi infection in infants. J Trop Pediatr 1995; 41(1): 52-4.	Extended systematic review	✓				✓	
Rajashri P and Amar P. Study of antibiotic sensitivity pattern of *Salmonella* Typhi in tertiary care centre.International Journal of Healthcare & Biomedical Research 2017;6(1):75-80	Initial systematic review	✓	✓			✓	
Ramanan A, Pandit N, Yeshwanth M. Unusual complications in a multidrug resistant *Salmonella* Typhi outbreak. Indian Pediatr 1992; 29(1): 118-20.	Extended systematic review	✓					
Ramesh U, Das S, Balasubramanian A. Re-emergence of chloramphenicol-susceptible *Salmonella* Typhi and Paratyphi A strains in India. Indian J Med Microbiol 2016; 34(2): 262-3.	Initial systematic review	✓		✓		✓	✓
Ranju C, Pais P, Ravindran GD, Singh G. Changing pattern of antibiotic sensitivity of *Salmonella* Typhi. Natl Med J India 1998; 11(6): 266-7.	Initial systematic review	✓					
Rao PS, Rajashekar V, Varghese GK, Shivananda PG. Emergence of multidrug-resistant *Salmonella* Typhi in rural southern India. Am J Trop Med Hyg 1993; 48(1): 108-11.	Initial systematic review	✓		✓			

Rao RS, Amarnath SK, Sujatha S. An outbreak of typhoid due to multidrug resistant *Salmonella* Typhi in Pondicherry. Trans R Soc Trop Med Hyg 1992; 86(2): 204-5.	Extended systematic review	✓					
Rasaily R, Dutta P, Saha MR, Mitra U, Lahiri M, Pal SC. Multi-drug resistant typhoid fever in hospitalised children. Clinical, bacteriological and epidemiological profiles. Eur J Epidemiol 1994; 10(1): 41-6.	Initial systematic review		✓				
Rathish KC, Chandrashekar MR, Nagesha CN. An outbreak of multidrug resistant typhoid fever in Bangalore. Indian J Pediatr 1995; 62(4): 445-8.	Initial systematic review	✓					
Rathish KC, Chandrashekar MR, Nagesha CN. Multidrug resistant *Salmonella* Typhi in Bangalore, south India. Indian J Med Sci 1994; 48(4): 85-8.	Initial systematic review	✓					
Rathore MH, Bux D, Hasan M. Multidrug-resistant *Salmonella* Typhi in Pakistani children: clinical features and treatment. South Med J 1996; 89(2): 235-7.	Initial systematic review	✓				✓	
Raveendran R, Wattal C, Sharma A, Oberoi J, Prasad K, Datta S. High level ciprofloxacin resistance in *Salmonella* enterica isolated from blood. Indian J Med Microbiol 2008; 26(1): 50-3.	Initial systematic review	✓	✓	✓	✓	✓	✓
Raza S, Tamrakar R, Bhatt CP, Joshi SK. Antimicrobial susceptibility patterns of *Salmonella* Typhi and *Salmonella* Paratyphi A in a tertiary care hospital. J Nepal Health Res Counc 2012; 10(22): 214-7.	Initial systematic review	✓		✓		✓	✓
Reddy KR, Rajesh PK, Krishnan M, Sekar U. Antibiotic susceptibility pattern and plasmid profile of multidrug resistant *Salmonella* Typhi. Indian J Med Microbiol 2005; 23(3): 208.	Extended systematic review	✓				✓	
Rehman N, Ahmed SI. Emergence of multi-drug resistance among beta-lactamase producing *Salmonella*. J Pak Med Assoc 1994; 44(11): 253-5.	Extended systematic review	✓		✓		✓	✓
Riyaz C, Jayavardhana A. Current pattern of *Salmonella* Typhi antimicrobial susceptibility in the era of antibiotic abuse. Indian J Basic Appl Med Res 2015; 5(1): 400-4.	Initial systematic review	✓	✓			✓	
Roberts T. LOMWRU blood culture database. Vientiane, Laos; 2019	Dataset	✓	✓	✓	✓	✓	✓

Rodrigues C, Mehta A, Joshi VR. *Salmonella* Typhiin the past decade: learning to live with resistance. Clin Infect Dis 2002; 34(1): 126.	Extended systematic review	✓	✓			
Rodrigues C, Mehta A, Mehtar S, et al. Chloramphenicol resistance in *Salmonella* Typhi. Report from Bombay. J Assoc Physicians India 1992; 40(11): 729-32.	Initial systematic review	✓				
Rodrigues C, Shenai S, Mehta A. Enteric fever in Mumbai, India: the good news and the bad news. Clin Infect Dis 2003; 36(4): 535.	Initial systematic review	✓	✓			
Rosana Y, Kiranasari A, Ningsih I, Tjampakasari C, Kadarsih R, Wahid MH. Patterns of bacterial resistance against Ceftriaxone from 2002 to 2005 in the clinical microbiology laboratory of the faculty of medicine, university of Indonesia. Med J Indones 2007; 16(1): 3-6.	Extended systematic review					✓
Rotimi VO, Jamal W, Pal T, Sonnevend A, Dimitrov TS, Albert MJ. Emergence of multidrug-resistant *Salmonella* spp. and isolates with reduced susceptibility to ciprofloxacin in Kuwait and the United Arab Emirates. Diagn Microbiol Infect Dis 2008; 60(1): 71-7.	Extended systematic review	✓	✓	✓	✓	✓
Rouahi N, Zouhdi M. Antibioresistance of Moroccan strains of *Salmonella* Typhi: a need for updating the standardized treatment of typhoid fever set on 1994. Ann Biol Clin (Paris) 2015; 73(6): 749-50.	Extended systematic review	✓				✓
Roy JS, Lahari S, Mithu M, Dipak T. Epidemiological investigation of an outbreak of typhoid fever in Jorhat town of Assam, India. Indian J Med Res 2016; 144(4): 592-6.	Extended systematic review	✓				✓
Rupali P, Abraham OC, Jesudason MV, et al. Treatment failure in typhoid fever with ciprofloxacin susceptible *Salmonella* enterica serotype Typhi. Diagn Microbiol Infect Dis 2004; 49(1): 1-3.	Initial systematic review	✓	✓			
Sabharwal ER. Ceftriaxone resistance in *Salmonella* Typhi - myth or a reality! Indian J Pathol Microbiol 2010; 53(2): 389.	Initial systematic review	✓				✓
Sabherwal U, Chaudhary U, Saini S. Multidrug-resistant *Salmonella* Typhi in Haryana in 1989-90. Indian J Med Res 1992; 95: 12-3.	Initial systematic review	✓				

Sabiha ST, Nilekar SL. Phage types of multidrug resistant *Salmonella* species in a rural area of Maharashtra, India. Bangladesh J Med Sci 2012; 11(1): 37-9.	Extended systematic review	✓		✓			
Sabine Vaccine Institute. Surveillance for Enteric Fever in Asia Project (SEAP) dataset. 2021	Dataset	✓	✓	✓	✓	✓	✓
Safdar A, Harjit K, Elting L, Rolston KVI. Antimicrobial susceptibility of 128 *Salmonella* enterica serovar Typhi and Paratyphi A isolates from Northern India. Chemotherapy 2004; 50(2): 88-91.	Extended systematic review	✓		✓		✓	✓
Saha MR, Dutta P, Bhattacharya SK, et al. Occurrence of multi-drug resistant *Salmonella* Typhi in Calcutta. Indian J Med Res 1992; 95(JULY): 179-80.	Initial systematic review	✓					
Saha MR, Dutta P, Niyogi SK, et al. Decreasing trend in the occurrence of *Salmonella* enterica serotype Typhi amongst hospitalised children in Kolkata, India during 1990-2000. Indian J Med Res 2002; 115(FEB.): 46-8.	Initial systematic review	✓					
Saha S, Saha S, Das RC, et al. Enteric Fever and Related Contextual Factors in Bangladesh.American Journal of Tropical Medicine & Hygiene 2018;99(3_Suppl):20-25	Initial systematic review	✓		✓		✓	✓
Saha SK, Darmstadt GL, Baqui AH, et al. Rapid identification and antibiotic susceptibility testing of *Salmonella* enterica serovar Typhi isolated from blood: implications for therapy. J Clin Microbiol 2001; 39(10): 3583-5.	Initial systematic review	✓					
Saha SK, Saha S, Ruhulamin M, Hanif M, Islam M. Decreasing trend of multiresistant *Salmonella* Typhi in Bangladesh. J Antimicrob Chemother 1997; 39(4): 554-6.	Initial systematic review	✓					
Saha SK. Antibiotic resistance of *Salmonella* Typhi in Bangladesh. J Antimicrob Chemother 1994; 33(1): 190-1.	Initial systematic review	✓	✓			✓	
Saharan G, Gupta A, Gupta BK, Sharma BP, Kochar DK, Purohit VP. Evaluation of antibiotic sensitivity pattern in cases of enteric fever in north west Rajasthan. J Indian Med Assoc 2008; 106(8): 528-30, 32.	Initial systematic review	✓		✓		✓	✓
Sanghavi SK, Mane MP, Niphadkar KB. *Salmonella* types and antibiotic susceptibility: a six months survey. Indian J Pathol Microbiol 2000; 43(1): 31-4.	Extended systematic review	✓					

Sania KM, Shyamasakhi PD, Krishna Pramodini KD, Sulochana KD. Evaluation of minimum inhibitory concentration of chloramphenicol for *Salmonella* spp. isolated from enteric fever cases in a tertiary hospital in Imphal. Int J Pharm Sci Res 2016; 7(9): 3815-9.	Initial systematic review	✓	✓			✓	
Saqib A, Ahmed A. Culture and sensitivity of *Salmonella* species: analysis of a two year data. J Pak Med Assoc 2000; 50(8): 282-4.	Initial systematic review					✓	✓
Secmeer G, Kanra G, Cemeroglu AP, Ozen H, Ceyhan M, Ecevit Z. *Salmonella* Typhi infections. A 10-year retrospective study. Turk J Pediatr 1995; 37(4): 339-41.	Initial systematic review	✓				✓	
Sekar U, Srikanth P, Kindo AJ, Babu VP, Ramasubramanian V. Increase in minimum inhibitory concentration to quinolones and ceftriaxone in *Salmonella*e causing enteric fever. J Commun Dis 2003; 35(3): 162-9.	Initial systematic review		✓				
Sen S, Goyal RS, Dev R. Ciprofloxacin in the management of multiple drug resistant typhoid fever. Indian Pediatr 1991; 28(4): 417-9.	Initial systematic review	✓	✓				
Senthilkumar B, Prabakaran G. Multidrug resistant *Salmonella* Typhi in asymptomatic typhoid carriers among food handlers in Namakkal district, Tamil Nadu. Indian J Med Microbiol 2005; 23(2): 92-4.	Extended systematic review	✓	✓				
Sethuraman S, Mahamood M, Kareem S. Furazolidone in multi-resistant childhood typhoid fever. Ann Trop Paediatr 1994; 14(4): 321-4.	Initial systematic review	✓					
Setiabudi D, Azhali MS, Garna H, Chairulfatah A. Antibiotic resistance patterns of pediatric typhoid fever at the Department of Child Health, Hasan Sadikin General Hospital, Bandung. Med J Indones 1998; 7: 289.	Initial systematic review	✓					
Seydi M, Soumare M, Sow AI, Diop BM, Sow PS. Current aspects of *Salmonella* bacteremia cases in the Ibrahima Diop Mar infectious diseases clinic, Fann national hospital center (Senegal). Med Mal Infect 2005; 35(1): 23-7.	Initial systematic review					✓	
Shahunja KM, Leung DT, Tahmeed A, et al. Factors associated with non-typhoidal *Salmonella* bacteremia versus typhoidal *Salmonella* bacteremia in patients presenting for care in an urban diarrheal disease hospital in Bangladesh. PLoS Negl Trop Dis 2015; 9(9).	Extended systematic review	✓				✓	
Shakoor S, Malik FR, Qamar FN, Hasan R. Aga Khan University Hospital, Karachi GRAM dataset. Karachi, Pakistan 2021	Dataset	✓	✓	✓	✓	✓	✓

Shanahan PM, Jesudason MV, Thomson CJ, Amyes SG. Molecular analysis of and identification of antibiotic resistance genes in clinical isolates of *Salmonella* Typhi from India. J Clin Microbiol 1998; 36(6): 1595-600.	Initial systematic review	✓					
Sharma NP, Peacock SJ, Phumratanaprapin W, Day N, White N, Pukrittayakamee S. A hospital-based study of bloodstream infections in febrile patients in Dhulikhel Hospital Kathmandu University Teaching Hospital, Nepal. Southeast Asian J Trop Med Public Health 2006; 37(2): 351-6.	Initial systematic review					✓	✓
Sharma P, Dahiya S, Balaji V, et al. Typhoidal *Salmonella*e: Use of multi-locus sequence typing to determine population structure. PLoS ONE 2016; 11(9).	Initial systematic review	✓	✓	✓	✓	✓	✓
Sharma P, Dahiya S, Manral N, et al. Changing trends of culture-positive typhoid fever and antimicrobial susceptibility in a tertiary care North Indian Hospital over the last decade.Indian Journal of Medical Microbiology 2018;36(1):70-76	Initial systematic review	✓	✓	✓	✓	✓	✓
Sharma, Sharma R, Gupta S. Bacteriological analysis of blood culture isolates with their antibiogram from a tertiary care hospital. Int J Pharm Sci Res 2015; 6(11): 4847-51.	Initial systematic review		✓			✓	
Sharvani R, Hemavathi, Dayanand DK, Shenoy P, Sarmah P. Antibiogram of *Salmonella* isolates: time to consider antibiotic salvage. J Clin Diagn Res 2016; 10(5): DC6-DC8.	Initial systematic review	✓	✓	✓	✓	✓	✓
Sheikh A, Charles RC, Rollins SM, et al. Analysis of *Salmonella* enterica serotype Paratyphi A gene expression in the blood of bacteremic patients in Bangladesh. PLoS Negl Trop Dis 2010; 4(12): e908.	Extended systematic review				✓		
Shen M, Wu X, Wang L. Serotypes and drug resistance of *Salmonella* isolated from clinical infection cases in Pinghu, Zhejiang. [Chinese]. Disease Surveillance 2014; 29(7): 560-3.	Extended systematic review					✓	✓
Sheorey HS, Kaundinya DV, Hulyalkar VS, Deshpande AK. Multi drug resistant *Salmonella* Typhi in Bombay. Indian J Pathol Microbiol 1993; 36(1): 8-12.	Extended systematic review	✓					
Sherchan JB, Morita M, Matono T, et al. Molecular and Clinical Epidemiology of *Salmonella* Paratyphi A Isolated from Patients with Bacteremia in Nepal. Am J Trop Med Hyg 2017; 09: 09.	Extended systematic review			✓	✓		✓

Shetty AK, Shetty IN, Furtado ZV, Antony B, Boloor R. Antibiogram of *Salmonella* isolates from blood with an emphasis on nalidixic acid and chloramphenicol susceptibility in a tertiary care hospital in coastal karnataka: a prospective study. J Lab Physicians 2012; 4(2): 74-7.	Initial systematic review	✓	✓	✓	✓	✓	✓
Shirakawa T, Acharya B, Kinoshita S, Kumagai S, Gotoh A, Kawabata M. Decreased susceptibility to fluoroquinolones and gyrA gene mutation in the *Salmonella* enterica serovar Typhi and Paratyphi A isolated in Katmandu, Nepal, in 2003. Diagn Microbiol Infect Dis 2006; 54(4): 299-303.	Initial systematic review	✓	✓	✓	✓	✓	✓
Shrestha KL, Pant ND, Bhandari R, Khatri S, Shrestha B, Lekhak B. Re-emergence of the susceptibility of the *Salmonella* spp. isolated from blood samples to conventional first line antibiotics. Antimicrob Resist Infect Control 2016; 5.	Initial systematic review					✓	✓
Shukun W, Congjia C, Yunbo Y, Biao K, Baowei D. Nalidixic acid resistance and clonal expansion of *Salmonella* enterica serotype Paratyphi A in Yuxi city, China. J Med Med Sci 2010; 17: 320-6.	Initial systematic review			✓	✓		
Shwe TN, Nyein MM, Yi W, Mon A. Blood culture isolates from children admitted to Medical Unit III, Yangon Children's Hospital, 1998. Southeast Asian J Trop Med Public Health 2002; 33(4): 764-71.	Initial systematic review	✓	✓			✓	
Siddiqui FJ, Rabbani F, Hasan R, Nizami SQ, Bhutta ZA. Typhoid fever in children: some epidemiological considerations from Karachi, Pakistan. Int J Infect Dis 2006; 10(3): 215-22.	Initial systematic review	✓		✓			
Singh U, Neopane A, Thapa M, Aryal N, Agrawal K. *Salmonella* Typhi infections and effect of fluroquinolones and third generation cephalosporins in clinical outcome. Journal of Nepal Paediatric Society 2011; 31(3): 216-21.	Initial systematic review	✓	✓			✓	
Singla N, Bansal N, Gupta V, Chander J. Outbreak of *Salmonella* Typhi enteric fever in sub-urban area of North India: a public health perspective. Asian Pac J Trop Med 2013; 6(2): 167-8.	Initial systematic review	✓	✓			✓	
Siourime SN, Isidore BOJ, Oumar T, et al. Serotyping and antimicrobial drug resistance of *Salmonella* isolated from lettuce and human diarrhea samples in Burkina Faso. African Journal of Infectious Diseases 2017; 11(2): 24-30.	Extended systematic review	✓	✓	✓	✓	✓	
Smith MD, Duong NM, Hoa NTT, et al. Comparison of ofloxacin and ceftriaxone for short-course treatment of enteric fever. Antimicrob Agents Chemother 1994; 38(8): 1716-20.	Extended systematic review	✓		✓		✓	✓

Soltan Dallal MM, Rastegar Lari A, Sharifi Yazdi MK. Pattern of serotyping and antibiotic resistance of *Salmonella* in children with diarrhea. [Persian]. Journal of Gorgan University of Medical Sciences 2014; 16(1): e100-Pe4.	Extended systematic review	✓	✓	✓	✓	✓
Somily AM, Sayyed SB, Habib HA, et al. *Salmonella* isolates serotypes and susceptibility to commonly used drugs at a tertiary care hospital in Riyadh, Saudi Arabia. J Infect Dev Ctries 2012; 6(6): 478-82.	Extended systematic review		✓			✓
Song Q, Xu Z, Gao H, Zhang D. Overview of the development of quinolone resistance in *Salmonella* species in China, 2005-2016. Infect Drug Resist 2018; 11: 267-74.	Extended systematic review		✓			
Song Q, Yang Y, Lin W, Yi B, Xu G. Epidemiological characteristics and clinical treatment outcome of typhoid fever in Ningbo, China, 2005-2014: Pulsed-field gel electorophoresis results revealing great proportion of common transmission sources. Jpn J Infect Dis 2017; 70(5): 513-7.	Extended systematic review	✓	✓			
Sood S, Kapil A, Das B, Jain Y, Kabra SK. Re-emergence of chloramphenicol-sensitive *Salmonella* Typhi. Lancet 1999; 353(9160): 1241-2.	Initial systematic review					✓
Sridhar CB, Kulkarni RD. Reassessment of frequency of occurrence of typhoid fever and cost efficacy analysis of antibiotic therapy. J Assoc Physicians India 1995; 43(10): 679-84.	Initial systematic review	✓				
Srikantiah P, Girgis FY, Luby SP, et al. Population-based surveillance of typhoid fever in Egypt. Am J Trop Med Hyg 2006; 74(1): 114-9.	Initial systematic review	✓				✓
Srirangaraj S, Kali A, Charles MV. A study of antibiogram of *Salmonella* enterica serovar Typhi isolates from Pondicherry, India. Australas Med J 2014; 7(4): 185-90.	Initial systematic review	✓	✓			✓
Stoesser N, Moore CE, Pocock JM, et al. Pediatric bloodstream infections in Cambodia, 2007 to 2011. Pediatr Infect Dis J 2013; 32(7): e272-e6.	Initial systematic review	✓				
Su LH, Chiu CH, Kuo AJ, et al. Secular trends in incidence and antimicrobial resistance among clinical isolates of *Salmonella* at a university hospital in Taiwan, 1983-1999. Epidemiol Infect 2001; 127(2): 207-13.	Extended systematic review					✓

Su LH, Wu TL, Chia JH, Chu C, Kuo AJ, Chiu CH. Increasing ceftriaxone resistance in *Salmonella* isolates from a university hospital in Taiwan. J Antimicrob Chemother 2005; 55(6): 846-52.	Extended systematic review	✓				✓	
Sucindar M and Kumaran SS. Profile of culture positive enteric fever in children admitted in a tertiary care hospital.Journal of Evolution of Medical and Dental Sciences-Jemds 2017;6(88):6112-6117	Initial systematic review		✓			✓	
Sudhaharan S, Padmaja K, Solanki R, Lakshmi V, Umabala P, Aparna B. Extra-intestinal salmonellosis in a tertiary care center in South India. J Infect Dev Ctries 2014; 8(7): 831-7.	Extended systematic review	✓				✓	
Sur D, Ochiai RL, Bhattacharya SK, et al. A cluster-randomized effectiveness trial of Vi typhoid vaccine in India. N Engl J Med 2009; 361(4): 335-44.	Initial systematic review	✓	✓				
Sur D, Barkume C, Mukhopadhyay B, et al. A Retrospective Review of Hospital-Based Data on Enteric Fever in India, 2014-2015.Journal of Infectious Diseases 2018;218(suppl_4):S206-S213	Initial systematic review	✓		✓		✓	✓
Suruchi B, Anil K, Ganju SA, Atal S. Antibiotic susceptibility pattern of *Salmonella* enterica serovar Typhi and Paratyphi a from North India: the changing scenario. Int J Pharma Bio Sci 2014; 5(4).	Initial systematic review	✓	✓	✓	✓	✓	✓
Taha RR, Alghalibi SM, Saeed Saleh MG. *Salmonella* spp. in patients suffering from enteric fever and food poisoning in Thamar city, Yemen. East Mediterr Health J 2013; 19(1): 88-93.	Extended systematic review	✓	✓			✓	
Tajbakhsh M, Hendriksen RS, Nochi Z, Zali MR, Aarestrup FM, Garcia-Migura L. Antimicrobial resistance in *Salmonella* spp. recovered from patients admitted to six different hospitals in Tehran, Iran from 2007 to 2008. Folia Microbiol (Praha) 2012; 57(2): 91-7.	Extended systematic review	✓	✓	✓	✓		
Takkar VP, Kumar R, Khurana S, Takkar R. Comparison of ciprofloxacin versus cephelexin and gentamicin in the treatment of multi-drug resistant typhoid fever. Indian Pediatr 1994; 31(2): 200-1.	Initial systematic review	✓					
Takkar VP, Kumar R, Takkar R, Khurana S. Resurgence of chloramphenicol sensitive *Salmonella* Typhi. Indian Pediatr 1995; 32(5): 586-7.	Initial systematic review	✓					

Tamang MD, Oh J, Seol S, et al. Emergence of multidrug-resistant *Salmonella* enterica serovar Typhi associated with a class 1 integron carrying the dfrA7 gene cassette in Nepal. Int J Antimicrob Agents 2007; 30(4): 330-5.	Initial systematic review	✓	✓	✓	✓		
Tambekar DH, Dhanorkar DV, Gulhane SR, Dudhane MN. Prevalence, profile and antibiotic susceptibility pattern of bacterial isolates from blood. Journal of Medical Sciences 2007; 7(3): 439-42.	Extended systematic review					✓	
Tankhiwale SS, Agrawal G, Jalgaonkar SV. A preliminary report on current antibiogram of *Salmonella* enterica serotype Typhi in Nagpur. Indian J Med Microbiol 2003a; 21(4): 292.	Initial systematic review	✓					
Tankhiwale SS, Agrawal G, Jalgaonkar SV. An unusually high occurrence of *Salmonella* enterica serotype Paratyphi A in patients with enteric fever. Indian J Med Res 2003b; 117: 10-2.	Initial systematic review			✓			
Tanmoy AM, Westeel E, Bruyne KD, et al. *Salmonella* enterica serovar Typhi in Bangladesh: exploration of genomic diversity and antimicrobial resistance.mBio 2018;9(6):02112-18	Initial systematic review	✓				✓	
Thamizhmani R, Bhattacharya D, Sayi DS, et al. Emergence of fluoroquinolone resistance in *Salmonella* enterica serovar Typhi in Andaman and Nicobar Islands, India. Indian J Med Res 2012; 136(1): 98-101.	Extended systematic review	✓	✓			✓	
Threlfall EJ, Ward LR, Rowe B, et al. Widespread occurrence of multiple drug-resistant *Salmonella* Typhi in India. Eur J Clin Microbiol Infect Dis 1992; 11(11): 990-3.	Initial systematic review	✓	✓				
Thriemer K, Ley B, Ame S, et al. The burden of invasive bacterial infections in Pemba, Zanzibar. PLoS ONE 2012; 7(2).	Initial systematic review	✓	✓			✓	
Tjaniadi P, Lesmana M, Subekti D, et al. Antimicrobial resistance of bacterial pathogens associated with diarrheal patients in Indonesia. Am J Trop Med Hyg 2003; 68(6): 666-70.	Extended systematic review	✓		✓		✓	✓
Trivedi ND, Trivedi UN. Case report and pathological result based study on typhoid patients. Int J Pharm Sci Rev Res 2010; 2(2): 22-3.	Initial systematic review	✓				✓	

Tunung R, Chai LC, Usha MR, et al. Incidence and characterization of *Salmonella* species in street food and clinical samples. Journal of Food Safety 2007; 27(4): 345-61.	Extended systematic review	✓	✓				
Turner P. GRAM_data_AHC-COMRU_2015. Cambodia; 2019.	Dataset	✓	✓			✓	
Uppal B, Perween N, Aggarwal P, Kumar SK. A comparative study of bacterial and parasitic intestinal infections in India. J Clin Diagn Res 2015; 9(3): DC01-4.	Extended systematic review		✓				
Vala S, Shah U, Ahmad SA, Scolnik D, Glatstein M. Resistance patterns of typhoid fever in children: A longitudinal community-based study. Am J Ther 2016; 23(5): E1151-E4.	Initial systematic review	✓	✓			✓	
Vandenberg O, Nyarukweba DZ, Ndeba PM, et al. Microbiologic and clinical features of *Salmonella* species isolated from bacteremic children in eastern Democratic Republic of Congo. Pediatr Infect Dis J 2010; 29(6): 504-10.	Initial systematic review	✓	✓			✓	
Venkatesh BM, Joshi S, Adhikary R, Bhaskar BH. Antibiogram of *Salmonella* Typhi and *Salmonella* Paratyphi A in a tertiary care hospital in 2012. Indian J Pathol Microbiol 2013; 56(4): 484-5.	Initial systematic review	✓	✓	✓	✓	✓	✓
Verma M, Chhatwal J, Saini V, Singh T. Enteric fever below 2 years of age. Indian Pediatr 1996; 33(3): 229-30.	Initial systematic review	✓					
Verma S, Thakur S, Kanga A, Singh G, Gupta P. Emerging *Salmonella* Paratyphi A enteric fever and changing trends in antimicrobial resistance pattern of *Salmonella* in Shimla. Indian J Med Microbiol 2010; 28(1): 51-3.	Extended systematic review	✓	✓	✓	✓		
Vidyalakshmi K, Yashavanth R, Chakrapani M, et al. Epidemiological shift, seasonal variation and antimicrobial susceptibility patterns among enteric fever pathogens in South India. Trop Doct 2008; 38(2): 89-91.	Initial systematic review	✓	✓	✓	✓	✓	✓
Vinh H, Duong NM, Phuong le T, et al. Comparative trial of short-course ofloxacin for uncomplicated typhoid fever in Vietnamese children. Ann Trop Paediatr 2005; 25(1): 17-22.	Initial systematic review	✓	✓				
Vlieghe ER, Phe T, De Smet B, et al. Azithromycin and ciprofloxacin resistance in *Salmonella* bloodstream infections in Cambodian adults. PLoS Negl Trop Dis 2012; 6(12): e1933.	Initial systematic review	✓	✓				

Vollaard AM, Ali S, van Asten HA, et al. Risk factors for typhoid and paratyphoid fever in Jakarta, Indonesia. JAMA 2004; 291(21): 2607-15.	Initial systematic review	✓		✓			
Wain J, Bay PVB, Vinh H, et al. Quantitation of bacteria in bone marrow from patients with typhoid fever: Relationship between counts and clinical features. J Clin Microbiol 2001; 39(4): 1571-6.	Initial systematic review	✓	✓				
Wain J, Diem Nga LT, Kidgell C, et al. Molecular analysis of incHI1 antimicrobial resistance plasmids from *Salmonella* serovar Typhi strains associated with typhoid fever. Antimicrob Agents Chemother 2003; 47(9): 2732-9.	Extended systematic review	✓					
Wain J, Diep TS, Ho VA, et al. Quantitation of bacteria in blood of typhoid fever patients and relationship between counts and clinical features, transmissibility, and antibiotic resistance. J Clin Microbiol 1998; 36(6): 1683-7.	Initial systematic review	✓					
Walters MS, Routh J, Mikoleit M, et al. Shifts in Geographic Distribution and Antimicrobial Resistance during a Prolonged Typhoid Fever Outbreak - Bundibugyo and Kasese Districts, Uganda, 2009-2011. PLoS Negl Trop Dis 2014; 8(3).	Extended systematic review	✓	✓				
Wan L, Xiao K, Ye X. Clinical analysis of typhoid fever and paratyphoid fever. [Chinese]. China Tropical Medicine 2008; 8(2): 225-6.	Extended systematic review	✓		✓			
Wang H, Qin B, Gao Y. Analysis of clinical characteristics and antimicrobial resistance of 24 cases of paratyphoid A. [Chinese]. Chongqing Medicine 2016; 45(22): 3083-5.	Extended systematic review						✓
Wang H, Yu H, Zheng W, et al. Genomic epidemiology of *Salmonella* Paratyphi A strains isolated in Hangzhou area. [Chinese].Chinese Journal of Microbiology and Immunology (China) 2018;38(2):116-123	Initial systematic review			✓			
Wang JL, Kao JH, Tseng SP, Teng LJ, Ho SW, Hsueh PR. Typhoid fever and typhoid hepatitis in Taiwan. Epidemiol Infect 2005; 133(6): 1073-9.	Extended systematic review		✓			✓	
Wang M, Kan B, Yang J, et al. Epidemiological characteristics of typhoid fever and antibiotic susceptibility testing of *Salmonella* Typhi isolates in Guangxi, 1994-2013 [Chinese]. Chung Hua Liu Hsing Ping Hsueh Tsa Chih 2014; 35(8): 930-4.	Initial systematic review	✓	✓				

Wankhede SV and Apurva. Prevalence and antibiogram of *Salmonella* Typhi and Paratyphi A isolates from a tertiary care hospital.Indian Journal of Basic and Applied Medical Research 2018;7(Diagnostic):31-38	Initial systematic review	✓		✓			
Wasfy MO, Frenck R, Ismail TF, Mansour H, Malone JL, Mahoney FJ. Trends of multiple-drug resistance among *Salmonella* serotype Typhi isolates during a 14-year period in Egypt. Clin Infect Dis 2002; 35(10): 1265-8.	Initial systematic review	✓				✓	
Wasfy MO, Moustafa DA, El-Gendy AM, et al. Prevalence of antibiotic resistance among Egyptian *Salmonella* Typhi strains. J Egypt Public Health Assoc 1996; 71(1-2): 149-60.	Extended systematic review	✓	✓			✓	
Wasihun AG, Wlekidan LN, Gebremariam SA, et al. Bacteriological profile and antimicrobial susceptibility patterns of blood culture isolates among febrile patients in Mekelle Hospital, Northern Ethiopia. Springerplus 2015; 4: 314.	Extended systematic review					✓	
Wasihun AG, Wlekidan LN, Gebremariam SA, et al. Diagnosis and Treatment of Typhoid Fever and Associated Prevailing Drug Resistance in Northern Ethiopia. Int J Infect Dis 2015; 35: 96-102.	Extended systematic review	✓				✓	
Wen-xiang H, Ji-zhao D, Qi-nan W, Xing-pin Z, Chong-zhi L. Plasmid profile analysis and antibiotics susceptibility testing of multiresistant S. Typhi [Chinese]. Chinese Journal of Antibiotics 1993; 18(3): 196-201.	Extended systematic review	✓					
Willke A. Low-level resistance to fluoroquinolones among *Salmonella* and Shigella [1]. Clin Microbiol Infect 2000; 6(12): 687.	Extended systematic review	✓	✓				
Wolday D, Erge W. Antimicrobial sensitivity pattern of *Salmonella*: comparison of isolates from HIV-infected and HIV-uninfected patients. Trop Doct 1998; 28(3): 139-41.	Extended systematic review	✓					
Woods CW, Murdoch DR, Zimmerman MD, et al. Emergence of *Salmonella* enterica serotype Paratyphi A as a major cause of enteric fever in Kathmandu, Nepal. Trans R Soc Trop Med Hyg 2006; 100(11): 1063-7.	Initial systematic review		✓	✓	✓	✓	✓
Wu W, Wang H, Lu J, et al. Genetic diversity of *Salmonella* enteric serovar Typhi and Paratyphi in Shenzhen, China from 2002 through 2007. BMC Microbiol 2010; 10: 32.	Initial systematic review	✓	✓	✓	✓	✓	✓

Yadav VC, Kiran VR, Sharma R. Enteric fever in Bastar tribal region-prevalence and sensitivity patterns. J Evol Med Dent Sci 2016; 5(53): 3526-30.	Initial systematic review	✓	✓	✓	✓	✓	✓
Yanagi D, de Vries GC, Rahardjo D, et al. Emergence of fluoroquinolone-resistant strains of *Salmonella* enterica in Surabaya, Indonesia. Diagn Microbiol Infect Dis 2009; 64(4): 422-6.	Initial systematic review	✓	✓			✓	
Yashavanth R, Vidyalakshmi K. The Re-Emergence of chloramphenicol sensitivity among enteric fever pathogens in Mangalore. J Clin Diagn Res 2010; 4(5): 3016-108.	Initial systematic review	✓	✓	✓	✓	✓	✓
Yousefi-Mashouf R, Moshtaghi A. Frequency of typhoidal and non-typhoidal *Salmonella* species and detection of their drugs resistance patterns. Journal of research in health sciences 2007; 7(1): 49-56.	Extended systematic review	✓		✓			
Yu AT, Amin N, Rahman MW and Luby S. Typhoid fever case fatality rate in patients presenting to a laboratory network in Dhaka, Bangladesh. American Journal of Tropical Medicine and Hygiene 2017;97 (5 Supplement 1):352	Initial systematic review	✓					
Yu F, Fan S, Fan X, et al. Analysis of characteristics of paratyphoid A in 157 Chinese inpatients between 1998 and 2009. Eur J Clin Microbiol Infect Dis 2011; 30(1): 71-5.	Initial systematic review			✓			✓
Yu R, Liang J, Xu H. Clinical analysis of 125 children with typhoid fever from 1993 to 2008 in Chongqing area. [Chinese]. Chinese Journal of Practical Pediatrics 2010; 25(7): 539-42.	Initial systematic review					✓	
Yu ZB, Yao C, Xiao YH, Cai SF. Multiple-antibiotic-resistant active efflux gene acrB and its expression in *Salmonella* Paratyphi A. [Chinese]. Chinese Journal of Antibiotics 2003; 28(12): 737-40+67.	Extended systematic review			✓			
Zehra NM, Irfan F, Mirza IA, et al. Current Trends of Antimicrobial Susceptibility of Typhoidal *Salmonella*e Isolated at Tertiary Care Hospital. Jcpsp, Journal of the College of Physicians & Surgeons - Pakistan 2017;27(11):690-692	Initial systematic review	✓		✓		✓	✓
Zellweger RM, Basnyat B, Shrestha P, et al. A 23-year retrospective investigation of *Salmonella* Typhi and *Salmonella* Paratyphi isolated in a tertiary Kathmandu hospital. PLoS Neglected Tropical Diseases [electronic resource] 2017;11(11):e0006051	Initial systematic review	✓	✓	✓		✓	✓

Zhang ZK, Huang YN, Guo BC, Deng ML, Yuan RZ, Wang QS. Surveillance of the antibiotic resistance and plasmid of *Salmonella* paratyphoid. [Chinese]. Chinese Journal of Antibiotics 2004; 29(10): 610-3.	Extended systematic review		✓	
Zhao JY, Zhang SY, Mu YJ, et al. Drug tolerance and PFGE molecular typing of *Salmonella* Paratyphi A isolated in Dengfeng, Henan province, 2009-2015 [Chinese]. Chung Hua Liu Hsing Ping Hsueh Tsa Chih 2016; 37(5): 714-7.	Initial systematic review		✓	✓
Zhuang L, Zhang YJ, Tang Z, et al. Epidemiologic characteristics of typhoid and paratyphoid fever on related drug resistance and molecular types regarding *Salmonella* Typhi and S. Paratyphi, in Jiangsu province [Chinese]. Chung Hua Liu Hsing Ping Hsueh Tsa Chih 2012; 33(12): 1269-72.	Initial systematic review	✓		✓

Table S2. Input data citations. Citations of all data input into the models and an indication for which model(s) each citation was used.

GATHER Statement

Item #	Checklist item	Reported on page #
**Objectives and funding**		
**1**	Define the indicator(s), populations (including age, sex, and geographic entities), and time period(s) for which estimates were made.	4
**2**	List the funding sources for the work.	5
**Data Inputs**		
For all data inputs from multiple sources that are synthesized as part of the study:		
**3**	Describe how the data were identified and how the data were accessed.	4
**4**	Specify the inclusion and exclusion criteria. Identify all ad-hoc exclusions.	4
**5**	Provide information on all included data sources and their main characteristics. For each data source used, report reference information or contact name/institution, population represented, data collection method, year(s) of data collection, sex and age range, diagnostic criteria or measurement method, and sample size, as relevant.	S5-S47
**6**	Identify and describe any categories of input data that have potentially important biases (e.g., based on characteristics listed in item 5).	9
For data inputs that contribute to the analysis but were not synthesized as part of the study:		
**7**	Describe and give sources for any other data inputs.	4
For all data inputs:		
**8**	Provide all data inputs in a file format from which data can be efficiently extracted (e.g., a spreadsheet rather than a PDF), including all relevant meta-data listed in item 5. For any data inputs that cannot be shared because of ethical or legal reasons, such as third-party ownership, provide a contact name or the name of the institution that retains the right to the data.	9
**Data analysis**		
**9**	Provide a conceptual overview of the data analysis method. A diagram may be helpful.	S3
**10**	Provide a detailed description of all steps of the analysis, including mathematical formulae. This description should cover, as relevant, data cleaning, data pre-processing, data adjustments and weighting of data sources, and mathematical or statistical model(s).	4-5
**11**	Describe how candidate models were evaluated and how the final model(s) were selected.	5
**12**	Provide the results of an evaluation of model performance, if done, as well as the results of any relevant sensitivity analysis.	S17
**13**	Describe methods for calculating uncertainty of the estimates. State which sources of uncertainty were, and were not, accounted for in the uncertainty analysis.	5
**14**	State how analytic or statistical source code used to generate estimates can be accessed.	5
**Results and Discussion**		
**15**	Provide published estimates in a file format from which data can be efficiently extracted.	
**16**	Report a quantitative measure of the uncertainty of the estimates (e.g. uncertainty intervals).	6-7
**17**	Interpret results in light of existing evidence. If updating a previous set of estimates, describe the reasons for changes in estimates.	7-8
**18**	Discuss limitations of the estimates. Include a discussion of any modelling assumptions or data limitations that affect interpretation of the estimates.	8-9

Table 3: GATHER Checklist: A checklist identifying each point of the Guidelines for Accurate and Transparent Health Estimates Reporting and whether they are covered in this study.

References

1. Browne AJ, Kashef Hamadani BH, Kumaran EAP, et al. Drug-resistant enteric fever worldwide, 1990 to 2018: a systematic review and meta-analysis. *BMC Med* 2020; **18**(1): 1.

2. GBD 2016 Risk Factors Collaborators. Global, regional, and national comparative risk assessment of 84 behavioural, environmental and occupational, and metabolic risks or clusters of risks, 1990-2016: a systematic analysis for the Global Burden of Disease Study 2016. *Lancet* 2017; **390**(10100): 1345-422.

3. Rue H, Martino S, Chopin N. Approximate Bayesian inference for latent Gaussian models by using integrated nested Laplace approximations. *Journal of the Royal Statistical Society: Series B (Statistical Methodology)* 2009; **71**(2): 319-92.

4. Stanaway JD, Reiner RC, Blacker BF, et al. The global burden of typhoid and paratyphoid fevers: a systematic analysis for the Global Burden of Disease Study 2017. *Lancet Infect Dis* 2019; **19**(4): 369-81.

5. Murray CJL, Ikuta KS, Sharara F, et al. Global burden of bacterial antimicrobial resistance in 2019: a systematic analysis. *Lancet* 2022; **399**(10325): 629-55.

Affiliations

Centre for Tropical Medicine and Global Health, Nuffield Department of Medicine (A J Browne DPhil, S J Dunachie PhD, F J Fell MSc, B H Kashef Hamadani DPhil, G Robles Aguilar PhD, B McManigal PhD, E A Ashley FRCPath, P N Newton FRCP, S Pokharel MD, B Sartorius PhD, C Dolecek PhD), Big Data Institute, Nuffield Department of Medicine (G Haines-Woodhouse MRes, E A P Kumaran MSc, N J Henry DPhil), Oxford Vaccine Group, Department of Paediatrics and the NIHR Oxford Biomedical Research Centre (A J Pollard FMedSci), University of Oxford, Oxford, UK; Child Health Analytics Programme (A J Browne DPhil), Telethon Kids Institute, Nedlands, Australia; Health and Wellbeing (M G Chipeta PhD), African Institute for Development Policy, Lilongwe, Malawi; Mahidol Oxford Tropical Medicine Research Unit (N P J Day DM), Mahidol University, Bangkok, Thailand; Oxford University Clinical Research Unit-Nepal (B Basnyat FRCPE, A Karkey PhD, M Shakya DPhil), Patan Academy of Health Sciences, Kathmandu, Nepal; Institute for Health Metrics and Evaluation (B Reiner PhD, J Stanaway PhD, M Naghavi PhD, S I Hay DSc; B Sartorius PhD), University of Washington, Seattle, WA, USA; Antimicrobial Resistance Research Group (C E Moore PhD), St George's, University of London, London, UK; Child Health Research Foundation (S K Saha PhD, S Saha PhD), Dhaka, Bangladesh; Applied Epidemiology Team (D O Garrett MD), Sabin Vaccine Institute, Washington, DC, USA; Cambodia Oxford Medical Research Unit (C Ngoun MD, C L Turner FRCPCH, P Turner FRCPath, Angkor Hospital for Children, Siem Reap, Cambodia; Cambridge Institute of Therapeutic Immunology and Infectious Disease (S G Baker PhD), Department of Medicine (G Dougan DSc, F Marks PhD), Wellcome Sanger Institute (Patrick Musicha PhD), University of Cambridge, Cambridge, UK; Center for Global Health (P K Munywoki PhD), Division of Global Health Protection (J R Verani MD), Center for Disease Control and Prevention, Nairobi, Kenya; Department of Clinical Sciences (N A Feasey PhD), Liverpool School of Tropical Medicine, Liverpool, UK; Department of Clinical Infection, Microbiology & Immunology (M A Gordon MD), University of Liverpool, Liverpool, UK; Malawi-Liverpool-Wellcome Trust Clinical Research Program (H C Banda MSc, A Chunga PhD), Queen Elizabeth Central Hospital, Blantyre,

Malawi; Department of Infection, Immunity and Cardiovascular Disease, Division of Clinical Medicine, School of Medicine and Population Health, (J Meiring DPhil), University of Sheffield, Sheffield, UK; Department of Medicine (J R Andrews MD), Division of Infectious Diseases and Geographical Medicine (S P Luby MD), Stanford University, Stanford, CA, USA; Epidemiology, Public Health, Impact (EPIC) Unit (A H Haselbeck Dr rer medic, M Holm PhD, F Marks PhD, HJ Jeon MSc, G Pak MSc), Office of the Director General (J D Clemens MD), International Vaccine Institute, Seoul, Republic of Korea; Madagascar Institute for Vaccine Research (F Marks PhD, HJ Jeon, PhD), University of Antananarivo, Antananarivo, Madagascar; Infectious Disease Division (F Khanam MPhil, F Qadri PhD), International Centre for Diarrhoeal Disease Research, Bangladesh, Dhaka, Bangladesh; Centre for Global Health Research (A Audi MSc, G Bigogo PhD), Kenya Medical Research Institute, Kisumu, Kenya; Lao-Oxford-Mahosot Hospital-Wellcome Trust Research Unit (E A Ashley FRCPath, V Chansamouth MSc, V Davong BL, M Mayxay MD, P N Newton FRCP, K Phommasone PhD, S Rattanavong MD, A J Simpson FRCPath, T Roberts PhD, M Vongsouvath MD), Mahosot Hospital, Vientiane, Laos; Pathology and Laboratory Medicine (R Hasan PhD, F Riaz Malik, S Shakoor MBBS), Department of Paediatrics and Child Health (F N Qamar MBBS), Aga Khan University, Karachi, Pakistan; and, Research Department of Infection, Division of Infection and Immunity (R S Heyderman FMedSci), University College, London, London, UK; School of Public Health (B Sartorius PhD), Faculty of Medicine, The University of Queensland, Brisbane, Queensland, Australia.

Author Contributions

Conceptualisation

Christiane Dolecek, Simon I Hay and Annie J Browne

Data collation and curation

Annie J Browne collated and curated the data with input from Bahar H Kashef Hamadani.

Formal analysis

Benn Sartorius and Annie J Browne designed and ran the models. Annie J Browne undertook the analysis with oversight from Benn Sartorius and Christiane Dolecek.

Writing of the first draft of the manuscript

Annie J Browne, Christiane Dolecek

Development of methods or computational machinery

Annie J Browne, Michael G Chipeta, Frederick J Fell, Georgina Haines-Woodhouse, Allan Audi, Catrin E Moore, Patrick K Munywoki, Simon I Hay, Mohsen Naghavi, Benn Sartorius, and Christiane Dolecek

Providing critical feedback on methods or results

Annie J Browne, Michael G Chipeta, Bahar H Kashef Hamadani, Emmanuelle A P Kumaran, Gisela Robles Aguilar, Barney McManigal, Allan Audi, Stephen Baker, Angeziwa Chunga, Nicholas A Feasey, Andrea H Haselbeck, Nathaniel Henry, Marianne Holm, Farhana Khanam, Stephen P Luby, Catrin E Moore, Patrick K Munywoki, Andrew J Pollard, Firdausi Qadri, Sadia Shakoor, Mila Shakya, Paul Turner, Jennifer R Verani, Simon I Hay, Mohsen Naghavi, Benn Sartorius, and Christiane Dolecek

Providing critical feedback on data sources

Annie J Browne, Michael G Chipeta, Bahar H Kashef Hamadani, Emmanuelle A P Kumaran, Barney McManigal, Allan Audi, Jason R Andrews, Happy C Banda, Godfrey Bigogo, Chanpheaktra Ngoun, Vilada Chansamouth, Susanna J Dunachie, Melita A Gordon, Andrea H Haselbeck, Nathaniel Henry, Farhana Khanam, Hyon Jin Jeon, Stephen P Luby, Faisal Riaz Malik, Florian Marks, Mayfong Mayxay, Catrin E Moore, Patrick Musicha, Gideok Pak, Firdausi Qadri, Farah Naz Qamar, Sayaphet Rattanavong, Bobby Reiner, Samir K Saha, Senjuti Saha, Sadia Shakoor, Andrew J Simpson, Jeff Stanaway, Jennifer R Verani, Manivanh Vongsouvath, Nicholas P J Day, Mohsen Naghavi, Benn Sartorius, and Christiane Dolecek

Providing data

Jason R Andrews, Elizabeth A Ashley, Allan Audi, Stephen Baker, Happy C Banda, Buddha Basnyat, Godfrey Bigogo, Chanpheaktra Ngoun, Vilada Chansamouth, John D Clemens, Viengmon Davong, Gordon Dougan, Susanna J Dunachie, Nicholas A Feasey, Denise Garrett, Melita A Gordon, Rumina Hasan, Andrea H Haselbeck, Marianne Holm, Hyon Jin Jeon, Abhilasha Karkey, Farhana Khanam, Stephen P Luby, Faisal Riaz Malik, Florian Marks, Mayfong Mayxay, Barney McManigal, James E Meiring, Patrick K Munywoki, Patrick Musicha, Paul N Newton, Gideok Pak, Koukeo Phommasone, Andrew J Pollard, Firdausi Qadri, Farah Naz Qamar, Sayaphet Rattanavong, Bobby Reiner, Tamalee Roberts, Samir K Saha, Senjuti Saha, Sadia Shakoor, Mila Shakya, Andrew J Simpson, Jeff Stanaway, Claudia Turner, Paul Turner, Jennifer R Verani, Manivanh Vongsouvath, Simon I Hay, Nicholas P J Day, Mohsen Naghavi, and Christiane Dolecek

Extracting, cleaning or cataloguing data; or designing or coding figures and tables

Annie J Browne, Michael G Chipeta, Frederick J Fell, Georgina Haines-Woodhouse, Bahar H Kashef Hamadani, Emmanuelle A P Kumaran, Allan Audi, Angeziwa Chunga, Catrin E Moore, Gideok Pak, Mohsen Naghavi, Benn Sartorius, and Christiane Dolecek

Reviewing and revising the manuscript for important intellectual content

Annie J Browne, Michael G Chipeta, Georgina Haines-Woodhouse, Bahar H Kashef Hamadani, Emmanuelle A P Kumaran, Gisela Robles Aguilar, Jason R Andrews, Elizabeth A Ashley, Allan Audi, Happy C Banda, Buddha Basnyat, Godfrey Bigogo, Chanpheaktra Ngoun, Vilada Chansamouth, Angeziwa Chunga, Viengmon Davong, Gordon Dougan, Susanna J Dunachie, Nicholas A Feasey, Melita A Gordon, Rumina Hasan, Andrea H Haselbeck, Nathaniel Henry, Robert S Heyderman, Marianne Holm, Hyon Jin Jeon, Abhilasha Karkey, Farhana Khanam, Stephen P Luby, Faisal Riaz Malik, Florian Marks, Mayfong Mayxay, James E Meiring, Catrin E Moore, Patrick K Munywoki, Patrick Musicha, Koukeo Phommasone, Sunil Pokharel, Andrew J Pollard, Firdausi Qadri, Farah Naz Qamar, Sayaphet Rattanavong, Bobby Reiner, Tamalee Roberts, Samir K Saha, Senjuti Saha, Sadia Shakoor, Mila Shakya, Andrew J Simpson, Jeff Stanaway, Claudia Turner, Paul Turner, Jennifer R Verani, Manivanh Vongsouvath, Nicholas P J Day, Mohsen Naghavi, Simon I Hay, Benn Sartorius, and Christiane Dolecek

All authors had full access to all the data in the study, contributed to revisions, and approved the final manuscript and the decision to submit the manuscript for publication. Annie J Browne, Christiane Dolecek and Benn Sartorius had access to and verified the underlying data.

Declaration of interest

SP Luby has received financial support to cover his time working on typhoid research projects from the Bill and Melinda Gates Foundation.

AJ Pollard has received research grants from the Wellcome Trust, MRC and the Bill and Melinda Gates Foundation on typhoid vaccines. Oxford University has entered into a partnership with AstraZeneca on COVID19 vaccines. AJ Pollard is chair of the Department of Health and Social Care's Joint Committee on Vaccination and Immunization and was a member of WHOs Strategic Advisory Group of Experts until 2022.

NJ Henry reports having received grants from the Bill and Melinda Gates Foundation in the past 36 months. AJ Simpson reports having received funding from the Wellcome Trust for the present manuscript.

All other authors report no conflicts.
